# Non‐Uniform Sampling for Quantitative NOESY

**DOI:** 10.1002/mrc.5529

**Published:** 2025-05-16

**Authors:** William T. P. Darling, Sven G. Hyberts, Mate Erdelyi

**Affiliations:** ^1^ Department of Chemistry ‐ BMC Uppsala University Uppsala Sweden; ^2^ Center of Excellence for the Chemical Mechanisms of Life Uppsala University Uppsala Sweden; ^3^ Department of Biological Chemistry and Molecular Pharmacology Harvard Medical School Boston Massachusetts USA

**Keywords:** NOESY, non‐uniform sampling, NUS, quantitative NOE

## Abstract

Non‐uniform sampling (NUS) enables faster acquisition of NMR spectra. Concerns about spectral fidelity, particularly in high‐dynamic‐range experiments like NOESY, have limited its quantitative applications. In this study, we assessed whether optimised Poisson‐gap sampling schemes can generate high‐fidelity spectra suitable for quantitation and evaluated the effectiveness of NUS ranking tools, NUSscore and nus‐tool, in identifying optimal sampling schemes. A total of 25,000 Poisson‐gap sampling schemes were generated and ranked using NUSscore, with a subset of 11 of these spanning the score distribution, alongside 15 random‐shuffle and the highest and lowest scoring Poisson‐gap schemes determined using the signal apex‐to‐artefact ratio were used for comparison, all with 50% sampling coverage. Additionally, hybrid sampling schemes incorporating a long initial uniformly sampled section, termed US‐NUS hybrid schemes, were evaluated. Spectral fidelity was evaluated on interproton distance accuracy, including the proportion of retained interproton distances and their deviation from uniformly sampled reference spectra. NUSscore showed a strong correlation with spectral fidelity. The peak‐to‐sidelobe ratio implemented in nus‐tool showed no correlation, with the relative sensitivity metric showing a weak correlation. Signal‐to‐artefact apex ratio was also not predictive for identifying sampling schedules with maintained interproton distances. All Poisson‐gap sampling schemes however outperformed random‐shuffle. The US‐NUS hybrids demonstrated improved interproton distance conservation than traditional Poisson‐gap sampling schemes with a low seed dependence, making them a promising sampling schedule for quantitative NOESY analysis.

## Graphical abstract

The ability of Poisson‐gap, quantile‐directed and random‐shuffle non‐uniform sampling (NUS) schemes in maintaining the quantitativity of NOESY spectra as well as the effectiveness of the ranking tools NUSscore and nus‐tool in identifying optimal sampling schemes are assessed. A hybrid US‐NUS sampling scheme is shown to be superior for quantitative NOE applications.

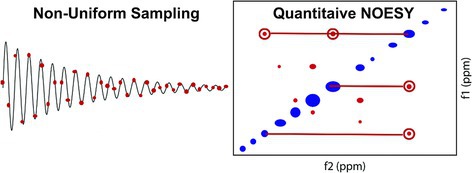

## 1 Introduction

Non‐uniform sampling (NUS) has allowed the acquisition of high‐quality nuclear magnetic resonance (NMR) spectra in a fraction of the time, with increased resolution or with increased sensitivity compared with standard uniform sampling (US) [1]. However, the improvement of one of these often comes at the expense of the other two. Non‐uniform sampling works by collecting a specific subset of datapoints in the time domain, dictated by a sampling scheme. These missing data are then reconstructed using advanced algorithms [2, 3, 4]. The implementation of NUS has facilitated the investigation of biological systems that would otherwise be impossible to probe due to the long acquisition time required for multidimensional NMR experiments during which proteins may decompose [5]. NUS has also offered significant time advantages in the structure determination of small molecules [6].

The influence of non‐uniform sampling on the quantitative accuracy of NOESY spectra however has for long been a matter of debate as spectra with high dynamic range are particularly susceptible to poor reconstruction [7]. Wieske et al. reported significant deviations in the length of interproton distances extracted from NOESY build‐up data between US and NUS datasets with sampling densities as high as 75% [8], particularly distances greater than 3.5 Å. Patel et al. instead found a good agreement for interproton distances between US and NUS spectra when studying a polysaccharide with only 40% coverage [9]; however, the study only included 16 short‐range proton pairs (< 3.36 Å). Similarly, Dass et al. found a good agreement between uniforml sampling and time‐resolved non‐uniform sampling interproton distances in strychnine; however, only short interproton distances (< 2.57 Å) were reported [10]. Nichols et al. assessed the application of non‐uniform sampling for 2D‐NOESY and 3D‐NOESY analysis of large biomolecules [11], where 3D structures calculated using NOE‐derived interproton distances were in good agreement between the uniformly sampled and varying degrees of non‐uniform sampling, as low as 10% total sampling. However, with sampling densities as high as 55%, up to 10% of interproton distances were missing in some cases, with longer distances more likely to be absent at lower sampling densities. Short‐range constraints can accurately describe the local secondary structures that form the foundations of the overall 3D structure; however, inaccuracy of long‐range constraints may pose severe limitations for determining the structure of protein–ligand complexes and solution ensembles of flexible midsize molecules, for instance.

A challenge of non‐uniform sampling is the variability introduced by the choice of random seed used to generate sampling schemes. Research has shown that the choice of seed number can have a large effect on the spectral quality when generating random schedules [12, 13]. Therefore, a number of methods have been developed in order to identify and to evaluate sampling schedules that produce high‐fidelity spectra. Unfortunately, the application of many of these methods is a non‐trivial affair for non‐specialists, or require prior knowledge of the system such as T2 relaxation rates [14]. A popular scoring method of sampling schemes is using the difference or *L* ^2^ norm between synthetic reconstructed NUS and US data [12]. This method is both time consuming and low throughput, requiring the reconstruction of each sampling schedule and the expertise to perform such comparisons. Often synthetic peaks or spectra are used, which may not accurately represent experimental data.

Sampling schedules can be generated from numerous different software. For non‐specialists, sampling schemes are typically generated in TopSpin (Bruker) using a user‐determined random seed value for random‐shuffle (RS) or Poisson‐gap (PG) sampling schemes [12]. These two classes of sampling can be selected by typing ‘nussptype 0’ or ‘nussptype 4’, respectively, into the command line within TopSpin. Online tools for the generation of sampling schemes are also available that are seed dependent [12] or seed independent [14, 15]. However, these easy‐to‐access sampling schedules are not specifically optimised for producing spectra with high‐fidelity.

To address the challenge of selecting optimal sampling schedules, various tools have been established. The software *NUSscore* was developed for high‐throughput generation and scoring of Poisson‐gap sampling schedules [16]. This scoring is based on the forward weighting (enhancing sensitivity) and random selection of points (reducing artefacts), wherein it was found that the best performing sampling schemes only make up a small percentage of the total randomly generated schemes [16]. The software nus‐tool [17], available via NMRbox [18], was also developed to generate sampling schemes using a variety of sampling methods such as exponential, quantile [14], random‐shuffle and Poisson‐gap. *Nus‐tool* can compute several metrics deriving from the point‐spread function (PSF) to help determine candidate sampling schedules for increased sensitivity, resolution and artefact suppression. Although *NUSscore* can evaluate thousands of schemes at once, *nus‐tool* is only capable of analysing sampling schemes individually through its graphical interface.

To increase the accessibility of high‐quality sampling schedules, libraries of ideal seeds have also been developed [19]. These libraries require the user to choose the desired sampling densities and the size of the Nyquist grid to acquire a sampling scheme that produces spectra with higher fidelity. These libraries have the benefit that no additional software is required. The seed values and sampling schemes of this library developed by Hyberts et al. were identified using the signal artefact‐to‐apex ratio (SAAR) [19].

We investigate herein whether there are sampling schedules that can maintain the quantitativity of NOEs and accordingly the derived interproton distances and whether such sampling schemes can be identified without prior knowledge of the system. To achieve this, the scoring metrics from *NUSscore* and *nus‐tool* were implemented to generate and identify high‐quality sampling schemes with a total sampling density of 50%. The resultant NUS spectra were then compared against a uniformly sampled dataset to evaluate the quantitativity of such sampling schemes.

## 2 Experiment

### 2.1 Generation of NUS Datasets

**FIGURE 1:**
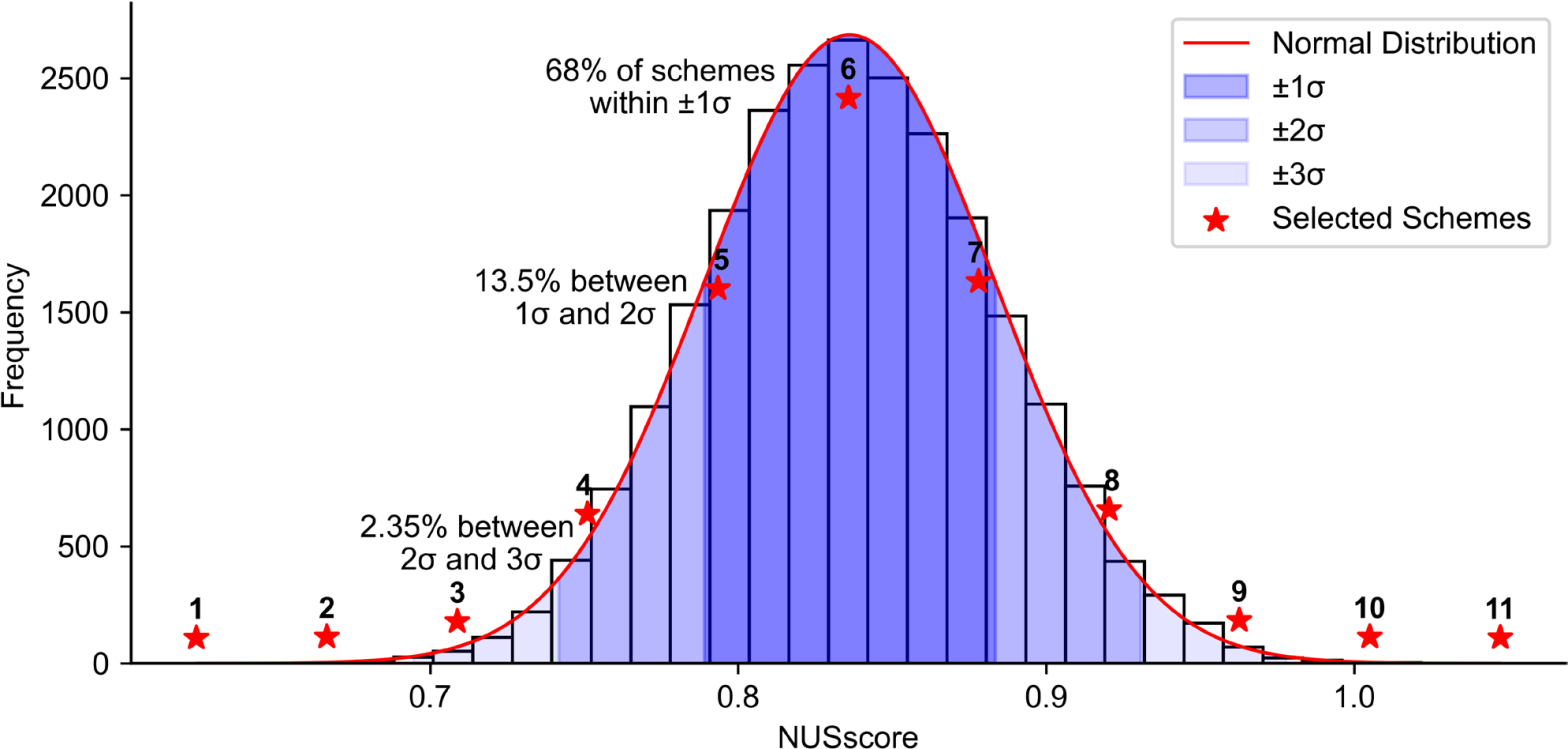
Distribution of NUS scores for 25,000 Poisson‐gap sampling schemes generated using *NUSscore*. The histogram (black) represents the frequency of scores, with a fitted normal distribution (red) overlaid. Shaded regions indicate a ±1σ (68% of schemes), +2σ or −2σ (13.5% of schemes) and +3σ or −3σ (2.35% of schemes). Red stars denote the 11 selected schemes (*NUSscore1*–*NUSscore11*), which span the full range from the best performing (*NUSscore1*) to the worst performing (*NUSscore* *11*) schedules. Lower NUSscore values correspond to higher ranked sampling schemes. A larger sample size than 25,000 is not expected to alter the observed trends.

*NUSscore* was used to generate 25,000 Poisson‐gap sampling schemes. These schemes yielded scores between 0.624 (best) and 1.05 (worst) with a normal distribution (Figure 1) [19]. Eleven evenly distributed schemes from best to worst were selected from this distribution (*NUSscore1*–*NUSscore11*) to assess the quality of an ‘average’ Poisson‐gap scheme, as well as the predicted best and worst performing schemes. This selection also allows us to evaluate whether the top‐scoring scheme truly represents the best performing scheme.

**FIGURE 2:**
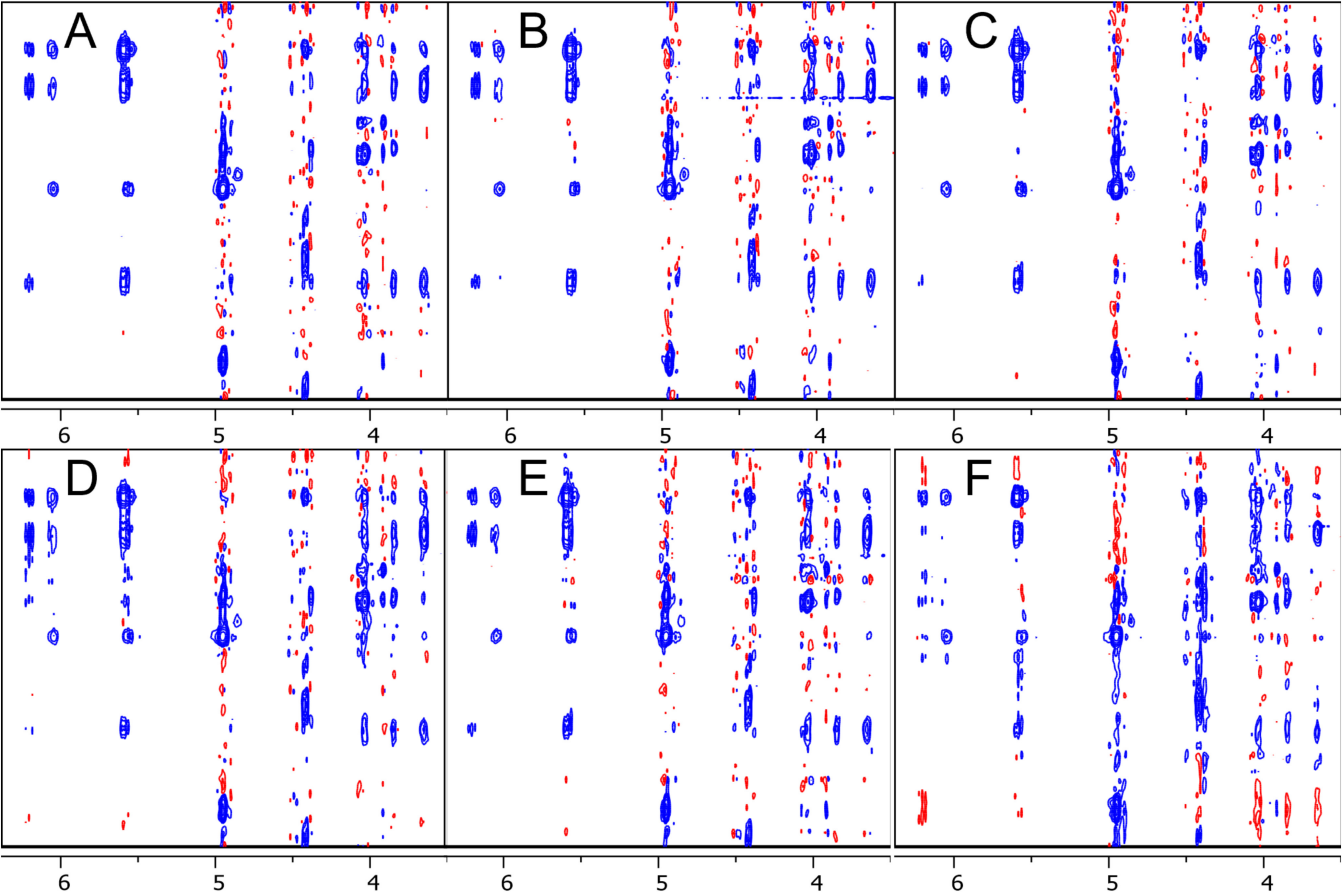
NOESY spectra regions of the US dataset (A), *NUSscore1* (B), *NUSscore6* (C), *NUSscore11* (D), *SAAR‐best* (E) and random‐shuffle (seed = 200, F).

In addition to the *NUSscore* generated schemes, we analysed the best and worst high‐fidelity seeds from the library curated by Hyberts et al. using the signal‐to‐artefact apex ratio (*SAAR‐best* and *SAAR‐worst*, respectively) and 15 random‐shuffle sampling schemes generated in TopSpin (Figure 2) [19]. Uniformly sampled 2D NOESY datasets of spiramycin, a model system that has previously been used to evaluate the influence of NUS on NOESY spectra, were converted to the corresponding NUS spectra using in‐house Python scripts within TopSpin.

## 3 Results

### 3.1 Comparison of NUS Scheme Scoring Metrics

Peak‐to‐sidelobe ratio, as implemented in the *nus‐tool* software, is a metric used to evaluate the likelihood of artefacts. It is derived from the point‐spread function that describes how an ideal single point spreads in the frequency domain due to the sampling schedule. The point‐spread function is first computed and plotted, and the relative intensity of the simulated peak is compared to its sidelobes, providing an estimate of artefact intensity. Similarly, the relative sensitivity metric quantifies the proportion of the signal envelope—which represents the overall ‘shape’ of signal intensity over time—that is captured by the sampling scheme. This metric is used to evaluate how effectively a scheme maximises peak intensity recovery. Relative sensitivity is improved either through a higher sampling density or by biasing the sampled points towards the start of the sampling schedule [20]. Although relative sensitivity is best suited towards comparing sampling schemes with different sampling densities or sampling types such as random‐shuffle, Poisson‐gap, or exponential, we investigated whether the relative sensitivity could also distinguish between good and bad seeds for Poisson‐gap sampling schemes due to differences in biasing of sampled points towards the start of the sampling schedule.

**FIGURE 3:**
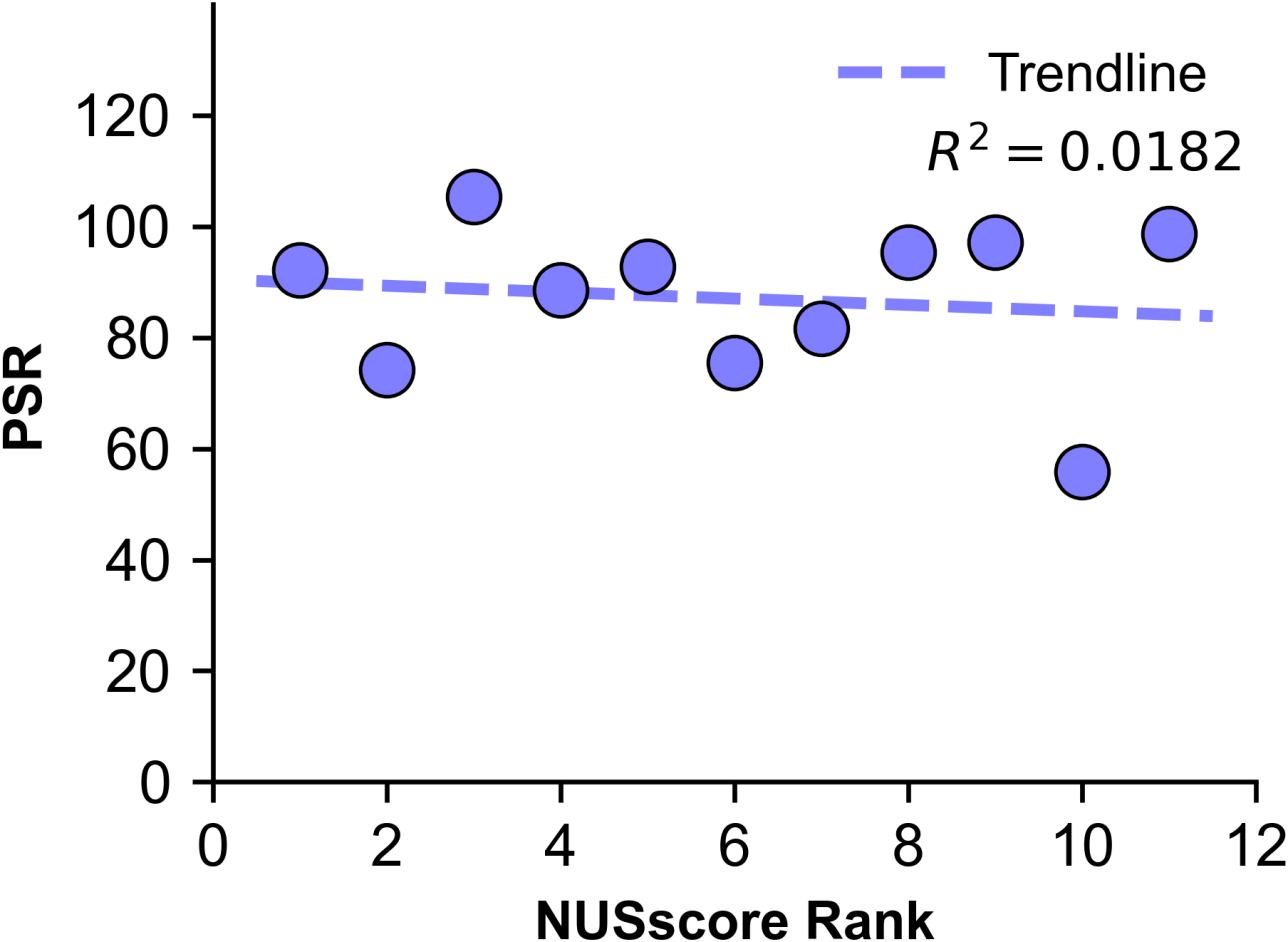
Correlation between *NUSscore* rank and peak‐to‐sidelobe ratio. Each point represents a Poisson‐gap sampling scheme with NUSscore rank ordered from best (1) to worst (11).

No correlation was observed between the *NUSscore* and the peak‐to‐sidelobe ratio (Pearson correlation coefficient *R* ^2^ = 0.018; Figure 3). This finding was slightly unexpected as, although the *NUSscore* is predominantly influenced by the forward weighting of the sampling scheme, the score is also dependent on the randomness of the selection of points and consequently the likelihood of artefacts. The peak‐to‐sidelobe ratio for the *NUSscore* Poisson‐gap schemes ranged from 55.8 to 105.3 (Table 1), with a wider spread than the random‐shuffle sampling schemes, which ranged between 78.0 and 100.5 (mean = 86.9). Notably, the peak‐to‐sidelobe ratio of *SAAR‐best* (59.9) was significantly lower than for *SAAR‐worst* (89.3).

**TABLE 1:** Nus‐tool metrics for each sampling scheme. Best values are highlighted in bold. The average value of all 15 random‐shuffle sampling schemes is given in addition to the range of values, given in parentheses.

Sampling scheme	Peak‐to‐sidelobe ratio	Relative sensitivity
*NUSscore1*	92.2	50.7
*NUSscore2*	74.2	50.5
*NUSscore3*	**105.3**	50.7
*NUSscore4*	88.6	**50.8**
*NUSscore5*	92.8	50.5
*NUSscore6*	75.5	50.5
*NUSscore7*	81.6	50.7
*NUSscore8*	95.4	50.3
*NUSscore9*	97.1	50.5
*NUSscore10*	55.8	50.3
*NUSscore11*	98.7	50.3
*SAAR‐best*	59.9	50.6
*SAAR‐worst*	89.3	50.5
*RS‐avg*	86.9 (65.6–101.0)	50.1 (50.0–50.3)

It is important to note that the peak‐to‐sidelobe ratio is calculated using the point‐spread function and treats missing data as zero‐filled prior to Fourier transform. However, modern NUS processing algorithms reconstruct the missing data instead of zero filling, suggesting that the signal‐to‐artefact apex ratio may be a more reliable metric for reconstructed NUS spectra.

A very weak positive correlation was seen between the relative sensitivity (calculated by *nus‐tool*) and *NUSscore* (*R* ^2^ = 0.50) owing to the biasing of sampled points to the start of the sampling schedule. Expectedly, all relative sensitivity values fell within a narrow range (50.3–50.8) due to the metric being predominantly influenced by the sampling density. As all sampling schemes described in this paper had a fixed sampling density of 50%, variations in relative sensitivity were minimal.

Notably, the top‐rated *NUSscore* dataset did not exhibit the highest peak‐to‐sidelobe ratio or relative sensitivity, suggesting that NUSscore does not directly predict or correlate with the *nus‐tool* metrics. For comparison, the random‐shuffle sampling schemes had relative sensitivities ranging between 50.0 and 50.3 (mean = 50.1), closely matching the sampling density of 50% due to the lack of biasing towards sampling early points. The signal‐to‐artefact apex ratio sampling schemes had similar relative sensitivity values to previous Poisson‐gap schemes of 50.5 for *SAAR‐worst* and 50.6 for *SAAR‐best*. These results are in agreement that the relative sensitivity metric is dictated primarily by the fraction of sampled points rather than the sampling distribution.

### 3.2 Fidelity of Non‐Uniformly Sampled NOESY Spectra

It has been previously shown that non‐optimal sampling schedules are unlikely to produce spectra appropriate for quantitative analysis. Therefore, we explored whether optimal sampling schedules could produce sufficiently high‐fidelity NOESY spectra for quantitative work and whether it was possible to identify these sampling schedules using *nus‐tool* or *NUSscore*. To study this, NOE build‐up rates and interproton distances were calculated for each NUS dataset and compared to a reference uniformly sampled (US) dataset. Build‐up rates calculated from seven different mixing times, as opposed to interproton distances calculated from a single mixing time, were determined in order to reduce the effect of specific noise fluctuations that may occur within a single spectrum. The following criteria were used to assess and compare sampling schedules with respect to their *NUSscore* and *nus‐tool* metrics:

- number of normalised NOEs with a linear build‐up rate
- number of interproton distances that exhibited large deviations from the US data
- average distance deviation from the US data

**FIGURE 4:**
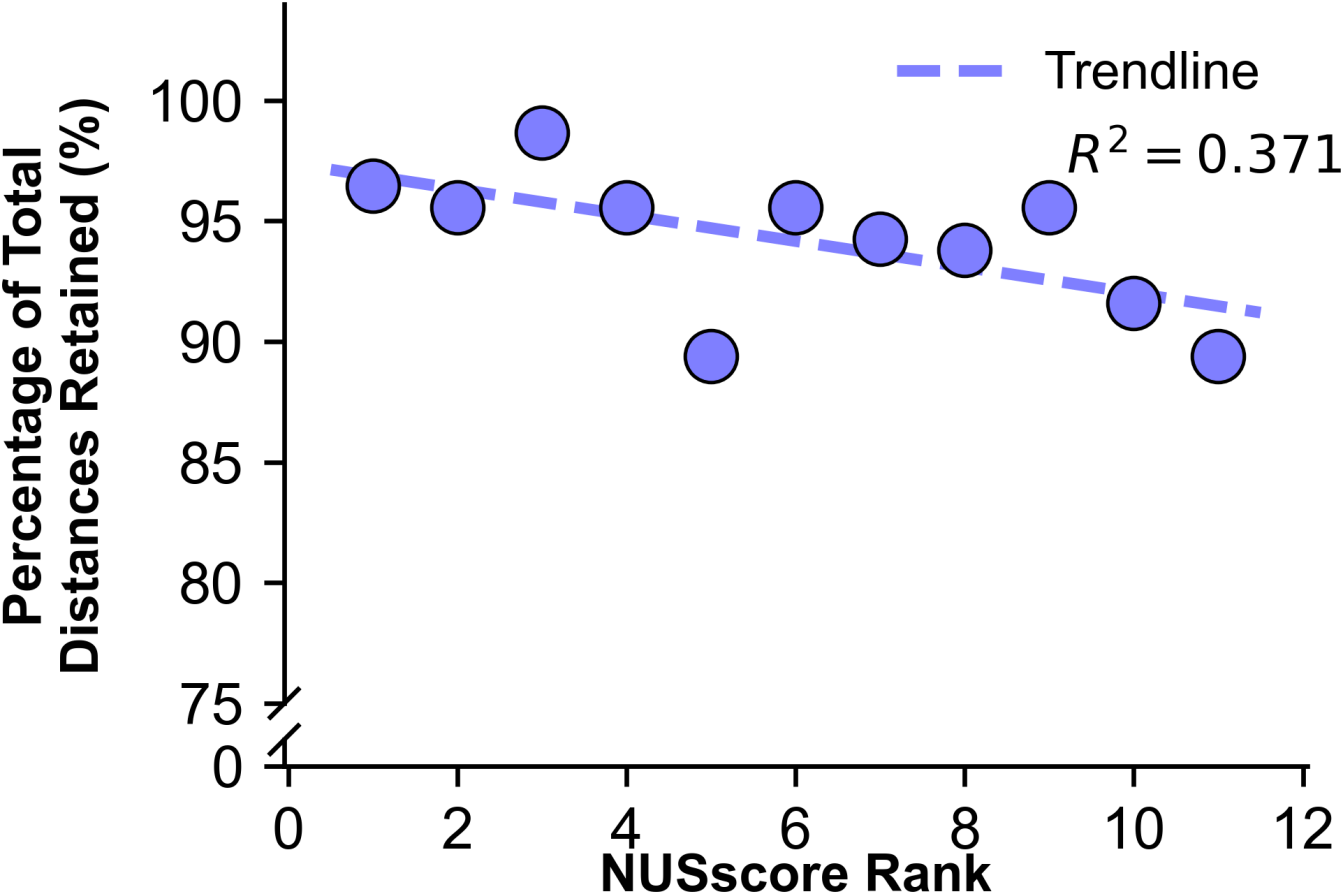
Correlation between NUSscore rank and the percentage of distances present compared to the US reference data. Each point represents a Poisson‐gap sampling scheme with NUSscore rank ordered from best (1) to worst (11).

In the US dataset, 226 valid interproton distances were identified. Within the *NUSscore* Poisson‐gap dataset, the number of valid interproton distances ranged from 202 to 223 (Table 2), with a poor correlation between *NUSscore* and total number of valid distances (*R* ^2^ = 0.37). The best performing sampling schedule was *NUSscore3*, which maintained 223 (98.7%) of the total interproton distances (Figure 4). By contrast, *NUSscore1*, the top ranked scheme, retained 218 (96.5%) of the total interproton distances.

**TABLE 2:** The total number of valid interproton distances and the percentage compared to the uniformly sampled reference data. Best values are highlighted in bold. The average value of all 15 random‐shuffle sampling schemes is given in addition to the range of values, given in parentheses.

	% of total distances	Total distances
*NUSscore1*	96.5%	218
*NUSscore2*	95.6%	216
*NUSscore3*	**98.7%**	**223**
*NUSscore4*	95.6%	216
*NUSscore5*	89.4%	202
*NUSscore6*	95.6%	216
*NUSscore7*	94.2%	213
*NUSscore8*	93.8%	212
*NUSscore9*	95.6%	216
*NUSscore10*	91.6%	207
*NUSscore11*	89.4%	202
*SAAR‐best*	94.7%	214
*SAAR‐worst*	96.9%	219
*RS‐avg*	81.3% (65.6–101)	183.8 (175–200)

*SAAR‐worst* outperformed *SAAR‐best* with regard to the number of interproton distances, identifying 219 versus 214 (96.9% vs 94.7%). In contrast, the random‐shuffle dataset consistently performed worse than even the worst Poisson‐gap sampling scheme, identifying only 175–200 valid interproton distances. Consistent with previous findings [8], the longer interproton distances were more likely to be missed (Figures S1–S3), particularly distances exceeding 3.5 Å. No correlation was observed between peak‐to‐sidelobe ratio and the number of valid distances (*R* ^2^ = 0.03), whereas relative sensitivity showed only a weak correlation (*R* ^2^ = 0.40), similar to *NUSscore*. These findings suggest that the number of linear NOE build‐up rates and therefore interproton distances are hard to predict using either *NUSscore* or *nus‐tool*.

Although a lack of interproton distances may increase the difficulty of NMR‐structure determination, it is unlikely to lead to an incorrect structure. Instead, erroneous distances, moreso than missing ones, pose a greater risk of leading to structural inaccuracies. With this in mind, we examined how many distances deviated substantially between the US and NUS datasets using this as a measure of potential error.

What constitutes a ‘substantial deviation’ depends on the required accuracy of interproton distances. For instance, larger errors may be more acceptable if using distance ranges rather than exact interproton distances. To minimise bias, we applied a range of deviation cut‐offs to classify distance errors as ‘acceptable’ or ‘unacceptable’.

With a strict deviation threshold (1%–5% deviation from the uniformly sampled data), a strong correlation was observed between *NUSscore* and the number of erroneous distances (*R* ^2^ = 0.83–0.90). With more realistic cut‐off values (6%–9%), we observed a moderate‐to‐strong correlation (*R* ^2^ = 0.62–0.76). For the more realistic cut‐off values, *NUSscore1* is also consistently the top performing sampling schedule (Table 3).

**TABLE 3:** Percentage of interproton distances that deviate by more than a given threshold (%) for different sampling schemes. Lower values indicate better agreement with the US reference dataset. NUSscore1–11 are the best‐to‐worst scoring NUS schemes as determined by NUSscore, respectively. Correlation values between NUSscore and the number of unacceptable deviations for each deviation cut‐off are given in the row NUSscore‐*R* ^2^.

	Proportion of distances deviating by more than x% (%)								
Deviation cut‐off	1%	2%	3%	4%	5%	6%	7%	8%	9%
*NUSscore1*	38.3	22.3	13.6	9.2	**5.3**	**2.9**	**1.5**	**0.5**	**0.5**
*NUSscore2*	**36.8**	**20.6**	13.7	9.3	6.9	3.4	2.9	1.5	**0.5**
*NUSscore3*	39.2	24.4	**12.4**	**7.2**	6.2	5.3	3.8	2.4	1.0
*NUSscore4*	42.9	25.9	18.9	13.2	7.1	4.7	2.4	0.9	**0.5**
*NUSscore5*	51.8	34.2	20.1	15.6	9.5	7.0	5.0	3.0	1.5
*NUSscore6*	51.5	31.5	19.5	13.0	8.5	5.0	3.5	2.5	2.5
*NUSscore7*	55.5	34.5	21.0	13.0	8.0	5.0	2.0	2.0	1.0
*NUSscore8*	52.3	33.5	21.3	17.3	11.7	8.1	5.1	3.6	2.5
*NUSscore9*	57.8	39.7	24.5	19.1	14.2	11.3	7.8	6.9	4.4
*NUSscore10*	57.0	37.8	27.5	18.7	15.0	8.3	5.7	4.7	2.1
*NUSscore11*	60.3	38.7	25.8	19.6	16.0	10.3	7.7	7.2	5.2
*SAAR‐best*	43.3	28.4	17.9	10.9	8.0	4.5	3.5	2.0	1.5
*SAAR‐worst*	48.0	28.0	19.0	13.0	8.0	5.5	3.5	3.0	2.0
*RS‐avg*	79.4	62.5	48.5	38.2	29.8	23.0	17.4	14.1	10.5
*NUSScore‐R* ^*2*^	0.90	0.88	0.90	0.83	0.88	0.76	0.62	0.75	0.68

Comparing the Poisson‐gap and random‐shuffle datasets at the realistic cut‐offs of 6%–9%, we observe that the Poisson‐gap datasets consistently exhibit three to four times fewer unacceptable deviations than the random‐shuffle datasets. However, rare cases were still observed in which a random‐shuffle dataset outperformed the worst Poisson‐gap datasets. There was no significant difference between the two signal‐to‐artefact apex ratio Poisson‐gap schemes, which both performed slightly better than the average *NUSscore* dataset (*NUSscore6*). Consistent with previous findings, longer interproton distances were also more prone to significant deviations from the US data (Tables S8–S11).

To evaluate the fidelity of reconstructed spectra for each sampling schedule, we also compared the average deviation of interproton distances (Table 4). That is, how far each interproton distance in the NUS datasets deviated from the reference US spectra, on average. For the *NUSscore* dataset, the average distance deviation ranged between 0.55% and 1.81%, consistently outperforming the random‐shuffle dataset which had deviations ranging from 2.33% to 4.02% (mean = 3.39%).

**TABLE 4:** The average distance deviation of each NUS scheme compared to the uniformly sampled reference data. Best values are highlighted in bold. The average value of all 15 random‐shuffle sampling schemes is given in addition to the range of values, given in parentheses.

	Average distance deviation (%)
*NUSscore1*	0.55
*NUSscore2*	0.53
*NUSscore3*	**0.49**
*NUSscore4*	0.70
*NUSscore5*	1.20
*NUSscore6*	1.50
*NUSscore7*	1.32
*NUSscore8*	1.44
*NUSscore9*	1.81
*NUSscore10*	1.43
*NUSscore11*	1.81
*SAAR‐best*	0.96
*SAAR‐worst*	0.80
*RS‐avg*	3.39 (2.34–4.02)

Both SAAR Poisson‐gap schemes had low distance deviations, however, *SAAR‐worst* (0.80%) performed marginally better than *SAAR‐best* (0.96%). Despite these low deviations, signal‐to‐artefact apex ratio‐based selection did not outperform the top ranking *NUSscore* schemes (*NUSscore1–4*).

**FIGURE 5:**
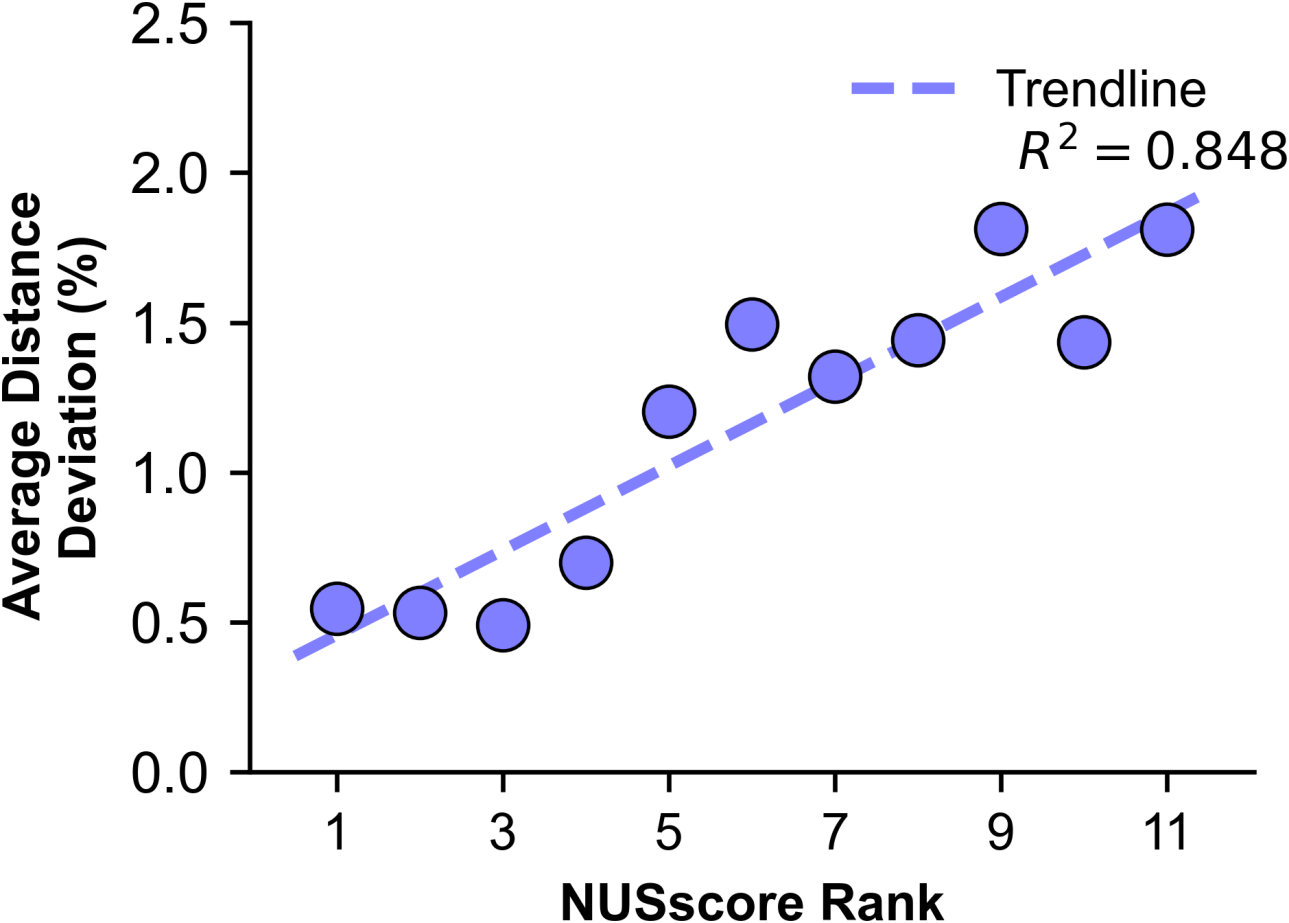
Correlation between NUSscore rank and the average distance deviation compared to the US reference data. Each point represents a Poisson‐gap sampling scheme with NUSscore rank ordered from best (1) to worst (11).

A strong correlation between *NUSscore* and the percentage distance deviation was observed (*R* ^2^ = 0.848), indicating that higher ranked *NUSscore* schedules retain better spectral fidelity (Figure 5). In contrast, a weak correlation was seen between relative sensitivity and distance deviation (*R* ^2^ = 0.442), whereas no correlation between peak‐to‐sidelobe ratio and distance deviation was observed (*R* ^2^ = 0.004).

These findings suggest that *NUSscore* is an accurate predictor of spectral fidelity, in this instance referring to the preservation of NOE intensity and chemical shift positions. Although chemical shift positions were not explicitly measured, they were well maintained for the sampling schedules with low distance deviations, as all datasets were processed using the same integration template. Despite not being designed as a predictor of spectral fidelity, relative sensitivity can be used as a moderate predictor by identifying sampling schemes with biasing towards the start of the sampling scheme. However, it is significantly less effective than *NUSscore*. The *nus‐tool* parameter peak‐to‐sidelobe ratio is unable to aid in identifying sampling schemes that preserve sensitivity.

*NUSscore* was also evaluated for its use with 25% sampling density; however, it was found that even the best sampling schemes were not sufficient at maintaining spectral fidelity. It was found that the highest scoring sampling scheme retained only 84.5% of interproton distances with an average distance deviation of 2.90%, which was deemed unacceptable for quantitative analysis. *NUSscore* still correlated well with the average distance deviation, but no correlation was seen for the percentage of total interproton distances (Table S4).

### 3.3 US‐NUS Hybrid Sampling Schemes

By analysing the Poisson‐gap sampling schemes that resulted in the smallest interproton distance deviations between NUS and US datasets, it was apparent that the forward weighting of the sampling schedules was the most important factor for maintaining high spectral fidelity. Therefore, a novel set of sampling schedules was designed with a long uniformly sampled section at the start of the sampling schedule, followed by a Poisson‐gap distributed NUS section utilising a random seed value.

Sampling schemes with initial uniformly sampled sections were previously investigated by Hyberts et al. [7], where 12.5% of the sampling scheme was uniformly sampled. Although these schemes could produce high‐quality spectra, as quantified using the deviation from a reference spectrum and the presence of minimal artefacts, a high seed dependence and the arbitrary length of the uniformly sampled section necessitated further optimisation of the strategy. ‘Bursty’ sampling approaches were developed by Maciejewski et al. [21], whereby uniform tracts in the sampling schedules are inserted to reduce aliasing and other spectral artefacts. More recently, sampling by quantiles developed by Craft et al. [14] produces long uniformly sampled tracts or highly dense regions at the beginning of the sampling scheme for the purpose of improving sensitivity rather than the reduction of aliasing and artefacts. Quantile sampling schemes are seed independent; however, changing biasing and evolution time parameters result in varying amounts of uniform sampling. Sampling schedules with short uniformly sampled regions have also been studied when comparing signal‐to‐artefact apex ratios of different sampling schemes [19], however with comparatively small uniformly sampled sections.

**FIGURE 6:**
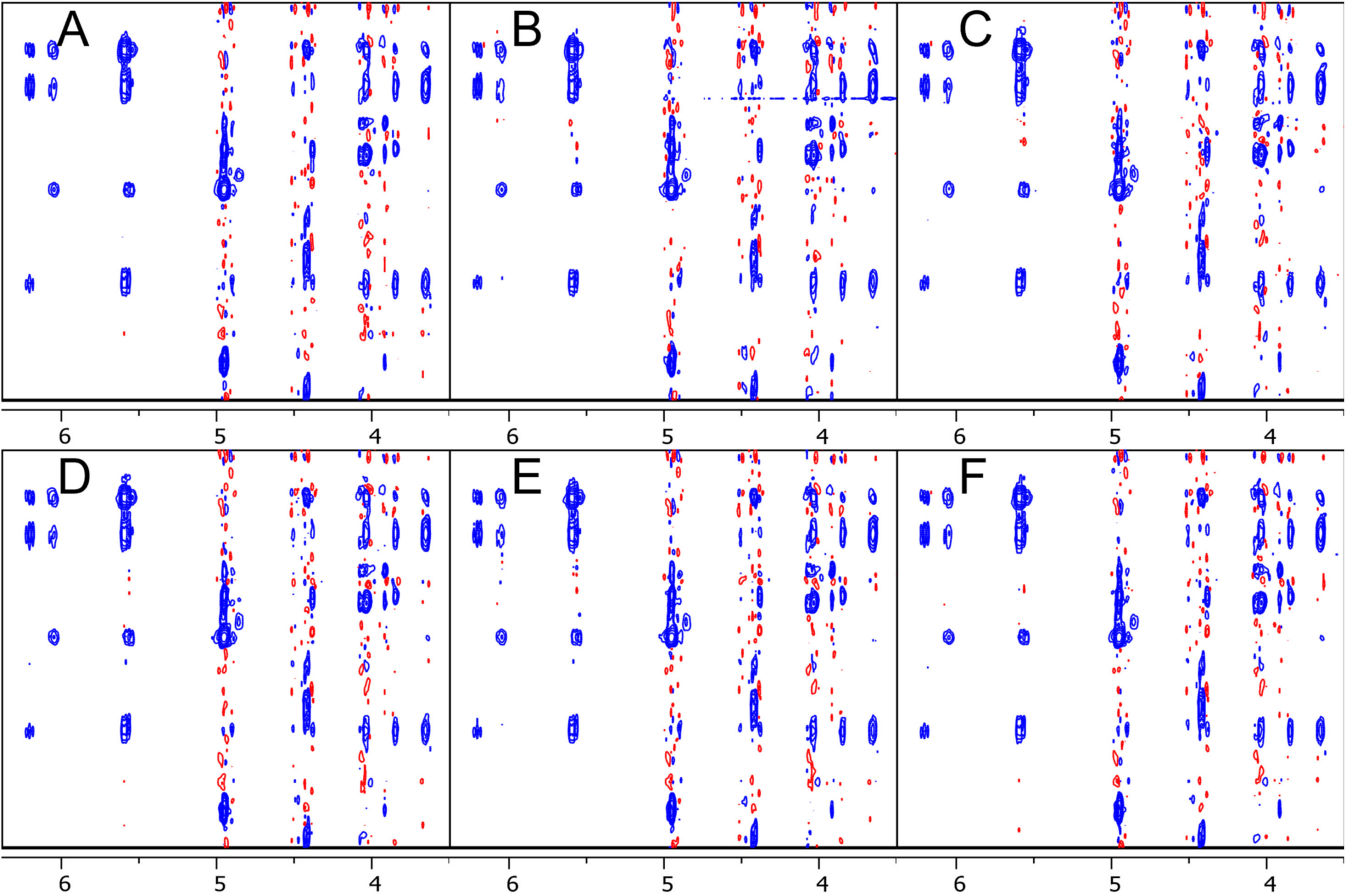
NOESY spectra regions of the US dataset (A), *NUSscore1* (B), and the hybrid sampling schedules 512‐50‐30‐0 (C), 512‐50‐40‐0 (D), and 512‐50‐45‐0 (E) and the quantile‐based sampling schedule (F).

This work explored the use of US‐NUS hybrid schedules with a Poisson‐gap distribution, comprising at least 25% uniform sampling, in order to maximise sensitivity and spectral fidelity. We also compared a quantile‐directed sampling scheme implemented in *nus‐tool* (Figure 6) [14, 17].

**FIGURE 7:**
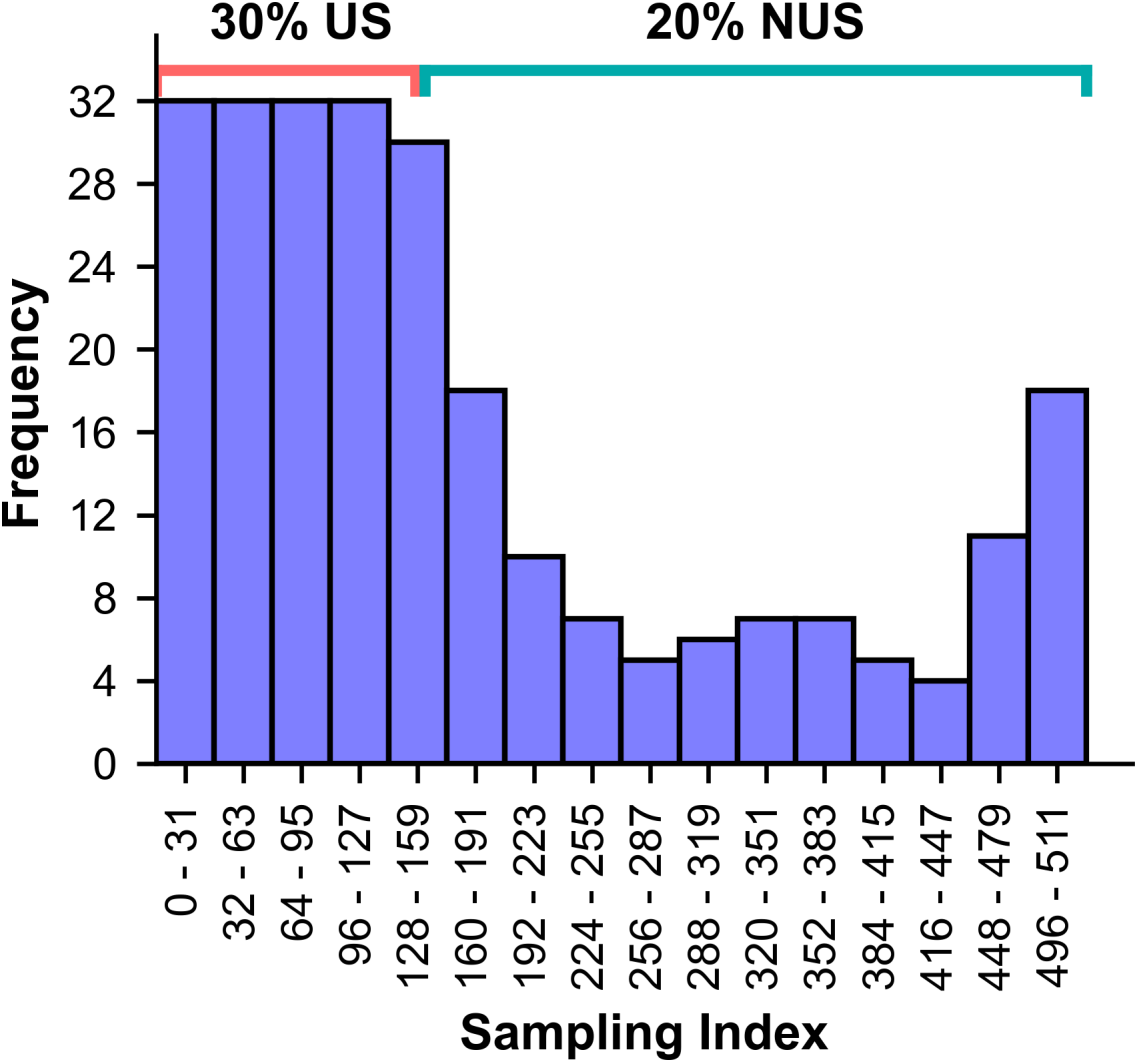
Histogram showing the distribution of sampled points in the sampling scheme of US‐NUS hybrid scheme 512‐50‐30‐0. The first 30% of points are uniformly sampled, followed by 20% of NUS using a Poisson‐gap distribution for a total sampling density of 50%.

The degree of US at the start of each sampling scheme was varied between 25% and 45%, with the remainder of the sampling schedule randomly sampled using a Poisson‐gap distribution to achieve a total sampling coverage of 50% and are referred to in the text as *512‐50‐x‐y* (size of the FID in the indirect dimension consisting of 512 hypercomplex points, 50% total sampling, *x*% US, *y* = seed; Figure 7). For each different proportion of US, three random seeds (0–2) were used to reduce outlier and biasing effects. The quantile sampling schedule comprised 207 uniformly sampled points, equivalent to 40% of the total 512 points, followed by 10% of NUS. These US‐NUS hybrids were then compared with traditional Poisson‐gap (*NUSscore1,6,11*) representing the ‘best predicted’ and ‘worst predicted’ schemes, as well as a typical scheme when using a random seed. The hybrid schemes were also compared to an average of the random‐shuffle sampling schemes. The schemes were compared in relation to the following:

- *Nus‐tool* metrics peak‐to‐sidelobe ratio and relative sensitivity
- presence of artefacts
- number of normalised NOEs with a linear build‐up rate
- number of interproton distances with large deviations from the US data
- magnitude of deviation of each distance from the US data

**FIGURE 8:**
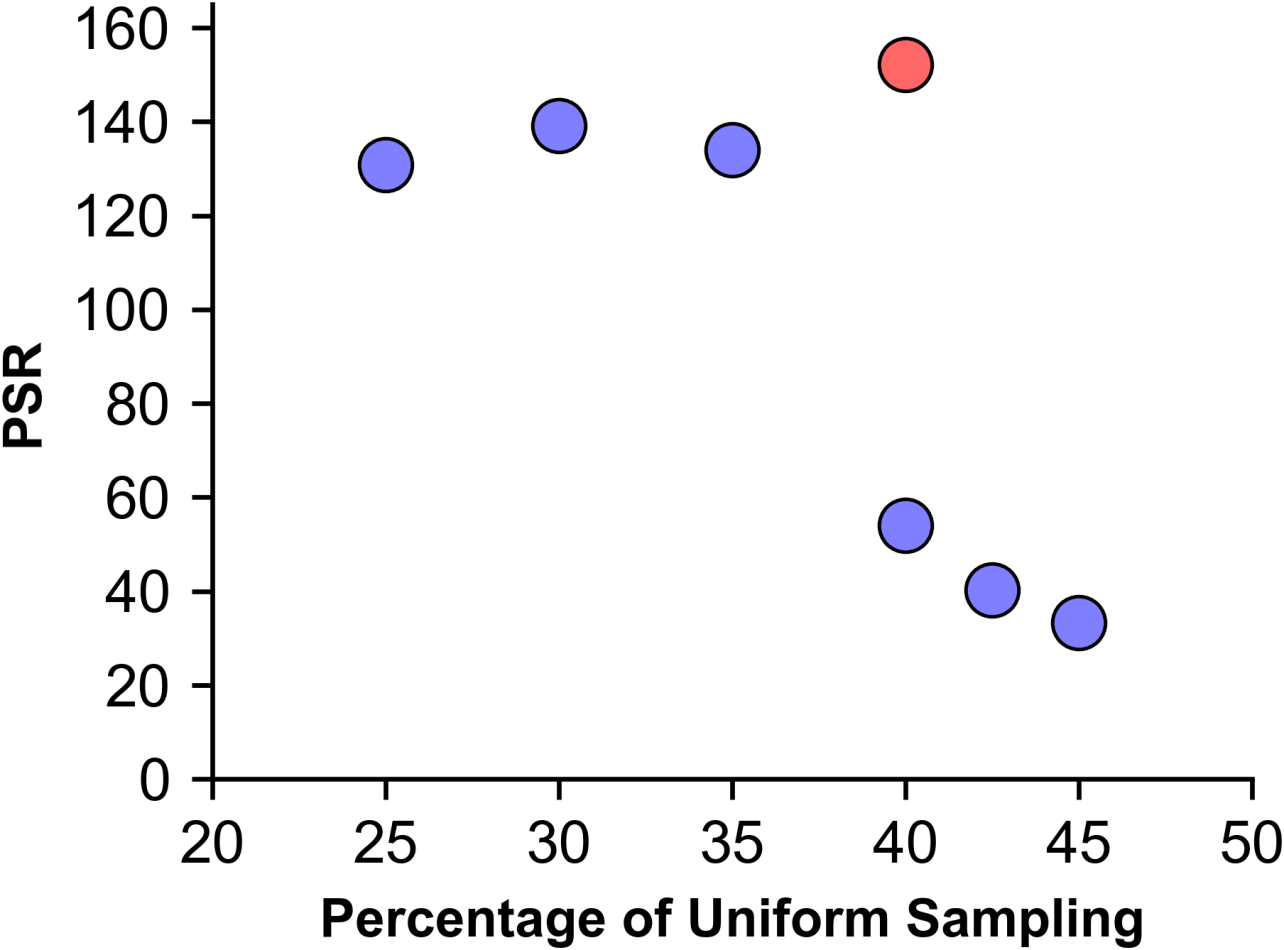
Relationship between the proportion of uniform sampling for the US‐NUS hybrid schemes (average of the seed values 0–2, blue) and the quantile‐directed sampling schedule (red) with the peak‐to‐sidelobe ratio.

The average of all three seeds per US proportion are hence used when comparing the *nus‐tool* metrics. The relative sensitivity metric in *nus‐tool* unsurprisingly scaled linearly with the proportion of US (*R* ^2^ = 0.98), with a narrow range of 50.6–51.1. The relative sensitivity of the hybrid sampling schemes were consistently higher than the Poisson‐gap sampling schemes. For 40%–45% US, the peak‐to‐sidelobe ratio was remarkably low (33.3–54.1; Figure 8) and were lower than any prior Poisson‐gap or random‐shuffle sampling scheme. In contrast, the 25%–35% US hybrid schemes showed peak‐to‐sidelobe ratios higher than any prior Poisson‐gap or random‐shuffle sampling schemes (130.8–139.2). A very high peak‐to‐sidelobe ratio was seen for the quantile‐directed sampling scheme (152.2). These findings suggest a high likelihood of artefacts with the PG US‐NUS hybrids for uniformly sampled proportions above 40%, but a low likelihood for those between 25% and 35% and the quantile‐directed scheme. In order to test this, the presence of artefacts was determined by visually inspecting the US‐NUS hybrid spectra in addition to the difference spectra between the US reference spectra and the US‐NUS hybrids.

**FIGURE 9:**
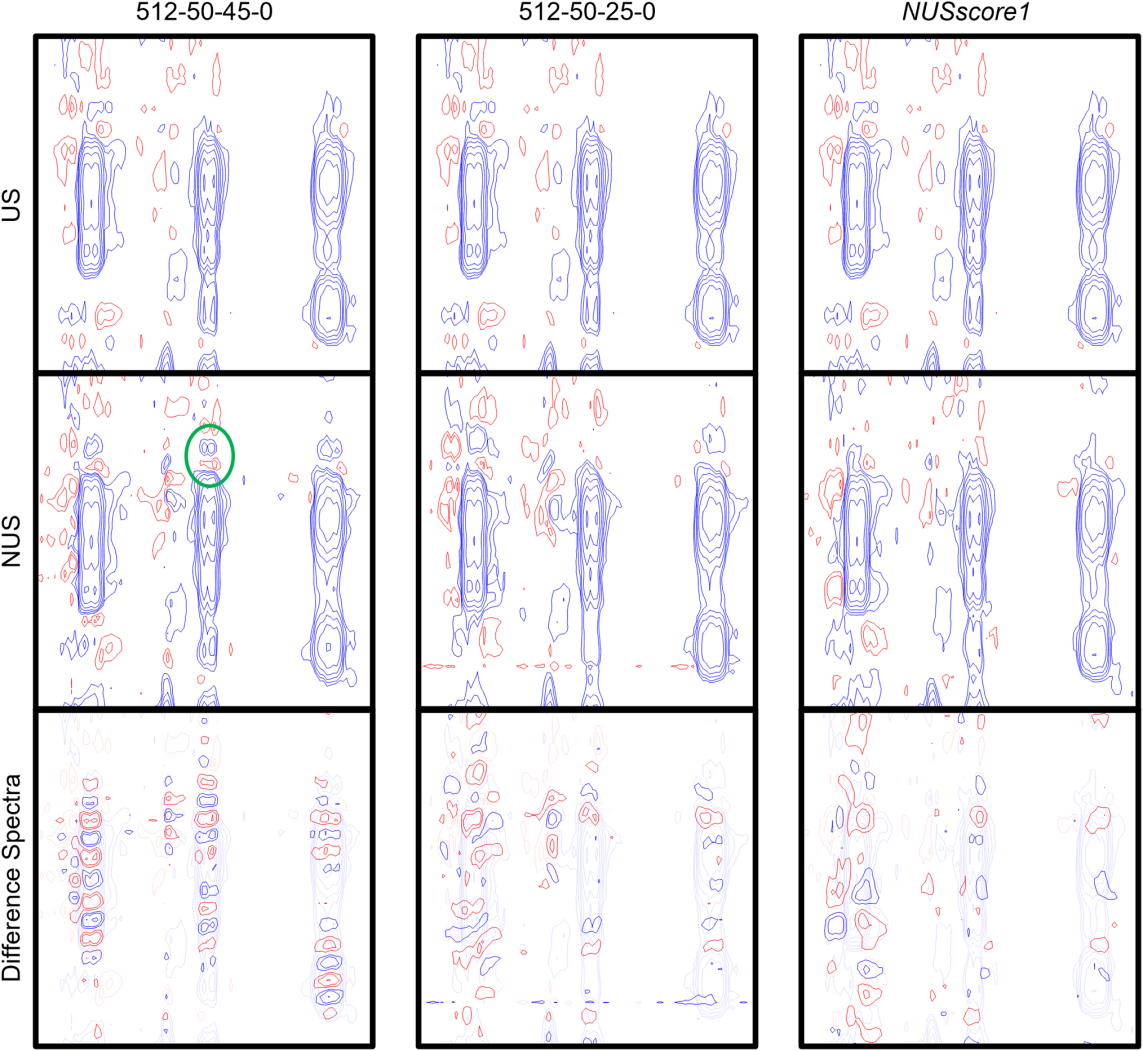
Sinc‐wiggle artefacts present in the US‐NUS hybrid spectra. The top rows show the same section of the uniformly sampled spectrum (t_mix_ = 700 ms). The middle left is the spectrum generated from the hybrid sampling scheme 512‐50‐45‐0; the middle centre is the same region from 512‐50‐25‐0 and middle right the top scoring Poisson‐gap sampling scheme (NUSscore1). The bottom panels display the difference spectra between the US and NUS spectra showing the sinc‐wiggle artefacts. A visible artefact in the NUS spectrum is highlighted by a green circle. No artefacts are seen in the 512‐50‐25‐0 or NUSscore1 NUS spectra.

In all US‐NUS/US reference difference spectra, the presence of antiphase ‘sinc wiggle’‐type artefacts in f1, or ‘ringing artefacts’ were identified beneath some strong cross‐peaks. These artefacts manifest as slight line broadening in f1, followed by signals appearing above or below the cross‐peak (Figure 9).

**FIGURE 10:**
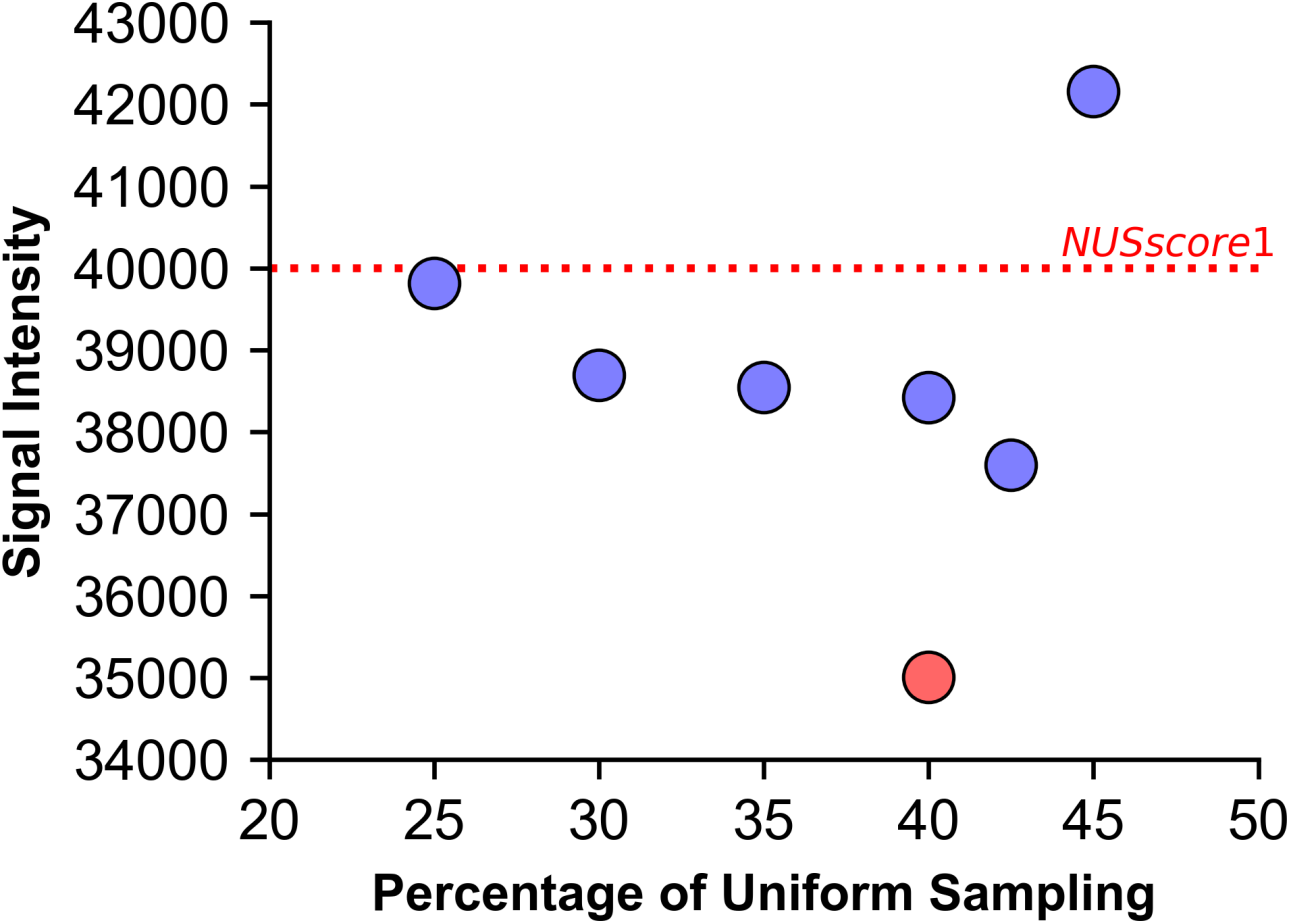
Correlation between proportion of the uniform sampling at the beginning of the US‐NUS hybrid schemes and the signal intensity within the difference spectra phased to magnitude‐mode. Each point represents a US‐NUS hybrid scheme with seed = 0 (blue) or the quantile‐directed sampling schedule (red). The dotted red line represents the intensity of the same integral regions for NUSscore1.

To assess the relative intensity of these artefacts, the difference spectra of the NUS and the US reference spectra were phased to magnitude mode and artefact‐containing regions were integrated. This was conducted for the US‐NUS hybrid spectra (seed = 0), *NUSscore1*, *SAAR‐best* and *SAAR‐worst*. The integrated regions for the 25%–42.5% US hybrid schemes had similar intensities (37,600–39,800 units); however, the 45% US hybrid was more severely affected by the artefacts with integrals of 42,200 units (Figure 10). The quantile‐based sampling scheme had even lower artefact intensities than the PG hybrid schemes (35,000 units). There was no correlation between the peak‐to‐sidelobe ratio and the intensity of the difference spectra of the Poisson‐gap hybrid schemes (*R* ^2^ = 0.07); however, a very weak correlation was seen when incorporating the quantile‐based sampling scheme (*R* ^2^ = 0.31). Although no sinc‐wiggle artefacts were seen in the difference spectra of *NUSscore1*, the difference spectrum still had intensities of 40,000 units—higher than most US‐NUS hybrids (Figure 10). These intensities were marginally higher than what was observed for the 25%–42.5% US‐NUS hybrid spectra, indicative of a greater difference between the US and NUS spectra.

*SAAR‐best* and *SAAR‐worst* had intensities within the difference spectra of 41,700 and 46,000 respectively, consistent with their relative ranking of ‘best’ and ‘worst’. However, *SAAR‐best* was still worse than *NUSscore1* and the 25%–42.5% US‐NUS hybrid schemes. Although the 25%–42.5% US hybrid schemes had less intensity in the difference spectra than the traditional Poisson‐gap sampling schemes, it is worth noting that no visible artefacts were observed within the *SAAR‐best* or *NUSscore1* spectra.

Comparison of *NUSscore6,11* of any random‐shuffle spectra using difference spectra was not feasible due to the large difference in peak intensity between the reference US and NUS spectra, resulting in strong in‐phase peaks and masking potential sinc‐wiggle artefacts. However, no artefacts were observed in these NUS spectra.

Sinc‐wiggles, or ringing artefacts, are commonly observed upon incomplete sampling [19, 22]. Despite their presence, the artefacts in the hybrid spectra do not appear to influence the intensity and fidelity of the cross‐peaks due to the antiphase pattern. The artefacts are easily identifiable in the NUS spectra as their locations do not correspond with the chemical shifts of protons in both dimensions. In addition, the artefacts do not have a corresponding peak on the other side of the diagonal, preventing their misidentification as real cross‐peaks. However, the identification of artefacts may be more difficult in higher dimension spectra or certain heteronuclear 2D spectra in which the corresponding 1D ^1^H, ^13^C, or ^15^N spectra are not acquired separately or in spectra where peaks are not mirrored about the diagonal, such as HSQC.

For each of the Poisson‐gap US‐NUS hybrid schemes, the proportion of valid interproton distances compared to the US reference spectra ranged from 96.0% to 98.6% with almost no correlation to the proportion of US (*R* ^2^ = 0.37). The worst performing individual scheme was *512‐50‐35‐0* with 96% of total distances and the worst performing group, on average, was the 45% US group with 97.1%. The best performing groups were the 40% and 30% US schemes with 98.2% and 98.1% of total distances. Eighty‐nine percent of all the hybrid schemes retained more distances than *NUSscore1*, and all hybrid schemes performed better than all random‐shuffle schemes. The quantile‐based sampling scheme consisting of 40% initial uniform sampling performed as well as 30% and 40% groups, maintaining 98.2% of distances.

**FIGURE 11:**
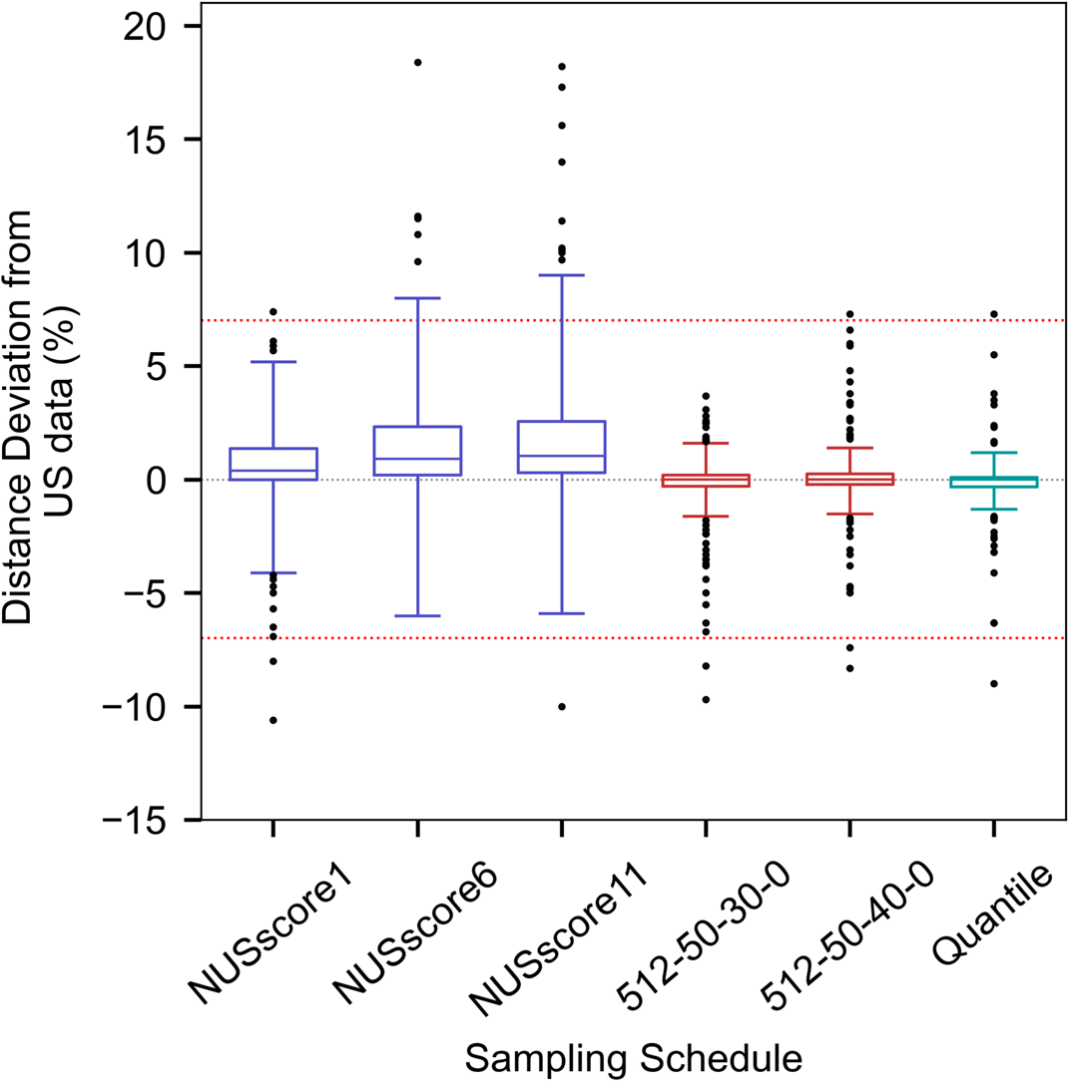
The error of the interproton for NUSscore sampling schemes and the hybrid schemes 512‐50‐30‐0 and 512‐50‐40‐0 and the quantile‐based scheme. The red dotted lines indicate an unacceptable distance deviation of ±7%. Whiskers extend to the furthest data point within three times the interquartile range from the first and third quartiles, with points beyond this threshold shown as black dots.

The number of distances with a ‘large’ or ‘unacceptable’ deviation was compared between the US‐NUS hybrids and the *NUSscore1,6,11* and average random‐shuffle schemes. At low cut‐offs of 1%–5%, even the worst US‐NUS hybrid schemes consistently had fewer large‐deviations than *NUSscore1,6,11* and the average RS. At higher deviation cut‐offs of 6%–9%, the hybrid schemes consistently performed better than *NUSscore6,11* and the average RS, and in 82% of cases, the hybrid schemes had fewer large deviations than *NUSscore1* (Figure 11). These findings suggest that the hybrid schemes are more suitable for generating NUS spectra with reduced errors. No consistent correlation between the proportion of US and the number of distances with large deviations was found for the different percentage cut‐offs, but generally, the higher proportion of US resulted in marginally fewer erroneous distances.

Finally, the average distance deviation between the US‐NUS hybrids and the reference US data was compared against *NUSscore1,6,11* and the average random‐shuffle datasets. For each proportion of uniform sampling in the hybrid spectra, the average of all three seed values was used. In all cases, the average distance deviation for the hybrid schemes was smaller than all Poisson‐gap and random‐shuffle schemes, including the top rated *NUSscore* scheme. The average distance deviation for the hybrid schemes ranged from −0.19% to 0.01% (−0.17% for the quantile‐based scheme), compared to 0.55%, 1.50% and 1.81% for *NUSscore1,6,11*, respectively, and 3.38% for the average random‐shuffle sampling scheme. When considering only the magnitude of the deviation and not the sign, a weak‐to‐moderate correlation between the proportion of US and the average distance deviation is seen (*R* ^2^ = 0.57) whereby the highest proportion of US leads to the smallest distance deviation.

## 4 Conclusion and Discussion

The impact of NUS on the fidelity of spectra with high dynamic range heavily depends on the choice of sampling scheme. For quantitative work, such as the extraction of interproton distances from NOESY spectra, the choice of sampling schedule or seed values is paramount for acquiring high‐fidelity spectra. Various methods have therefore been developed in order to determine optimal sampling schedules using a variety of metrics such as the signal‐to‐artefact apex ratio (SAAR), peak‐to‐sidelobe ratio (PSR), relative sensitivity, and *NUSscore*. However, the performance of these methods against each other and to uniformly sampled data has rarely been compared.

We used the total number of reliable interproton distances, the number of interproton distances that significantly deviate from the US spectra, and the average distance deviation between US and NUS spectra as three experimental observables to identify high‐quality sampling schedules for the acquisition of quantitative NOESY spectra. It is apparent that these three observables are only loosely correlated with one another and that having the lowest average distance deviation does not necessitate that the spectra also retain the highest number of reliable interproton distances. This implies that there is unlikely to be a single sampling scheme that performs best by all metrics.

Our findings demonstrate that *NUSscore* is a strong predictor of sampling schedule performance, with a weak correlation between *NUSscore* and the number of retained interproton distances (*R* ^2^ = 0.374) but a very strong correlation for percentage distance deviation and *NUSscore* (*R* ^2^ = 0.849). In contrast, the peak‐to‐sidelobe ratio exhibited no meaningful relationship (*R* ^2^ = 0.004). The peak‐to‐sidelobe ratio showed no correlation with any of the three observables (number of retained distances, number or erroneous distances, or average percentage distance deviation) used to evaluate spectral fidelity. These findings are consistent with Aoto et al. and Schuyler et al.'s findings that the point‐spread function ratio of sidelobes was unable to predict the amplitude of reconstruction artefacts and was not sufficient alone to screen for optimal schedules [16, 23]. Despite relative sensitivity primarily being used to evaluate different sampling densities or biasing, a weak correlation was seen when comparing the percentage distance deviation (*R* ^2^ = 0.442).

It is worth noting that although *NUSscore* is a strong predictor of sampling schedule performance, in this work the best scoring scheme *NUSscore1* was not the best performing in all metrics. Although *NUSscore1* had the fewest interproton distances with unacceptable deviations (at deviation cut‐offs between 6% and 9%), *NUSscore3* performed marginally better in terms of the average distance deviation and the number of total interproton distances. Therefore, *NUSscore* should be used as a tool to find high‐quality sampling schemes; however, identification of a definitive ‘best scheme’ is likely unattainable.

The signal‐to‐artefact apex ratio‐based selection was not able to outperform the best ranked *NUSscore* schedules, with *SAAR‐best* and *SAAR‐worst* both performing slightly better than the average Poisson‐gap sampling scheme (*NUSscore6*) regarding the number of erroneous distances and the average distance deviation. In most cases, *SAAR‐worst* performed better than *SAAR‐best* except in the measure of the difference‐spectra intensity. This suggests that the SAAR metric is more suited towards identifying NUS schemes with fewer artefacts than identifying schemes with maintained sensitivity. Using the same pool of 100 sets of Poisson‐gap sampled sequences that was used to determine *SAAR* values [19], an attempt was made to select based on optimised sensitivity. The resulting sequence was however not able to achieve better results than either *SAAR‐best* or *SAAR‐worst*.

The random‐shuffle datasets consistently performed worse than even the worst Poisson‐gap sampling schemes (as determined by *NUSscore*), and its use for quantitative work is therefore not recommended.

We have further studied US‐NUS hybrid sampling schedules for enhanced quantitativity. These consist of a long uniformly sampled section at the start of the sampling schedule, followed by a NUS section using a Poisson‐gap distribution. Such a hybrid scheme is demonstrated to have the same resolution, but increased sensitivity, compared to traditional Poisson‐gap sampling schemes with the same overall sampling density. The effect on the length of the uniformly sampled region has only a small effect on average distance deviation, with longer uniformly sampled sections resulting in smaller deviations. However, even with only 25% uniform sampling, the average distance deviation was lower than all previous Poisson‐gap schemes. In 89% of cases, the US‐NUS hybrids also had more total valid distances than *NUSscore1*. The Poisson‐gap hybrid schemes performed similarly to the quantile‐based sampling scheme when using the recommended parameters, which offers a seed‐independent sampling method.

Although sinc‐wiggle artefacts appeared in some US‐NUS hybrids, their presence did not affect peak intensities. The artefacts could be identified easily as they do not correspond to a proton chemical shift in both f1 and f2 in addition to the corresponding artefact on the opposite side of the diagonal not being found. As these artefacts are easily identifiable, they do not interfere with quantitative NOE analysis. However, the methods of detection for the artefacts may not be trivial in higher dimension spectra or experiments that are not symmetrical about the diagonal such as HSQC. Due to the stronger sinc‐wiggle artefacts found in the 45% US‐NUS hybrid spectra compared to the lesser percentages, such extreme amounts of uniform sampling should likely be avoided.

Whether it is more important to sample a particular number of uniformly sampled points at the start of a sampling schedule or instead if it is more important to sample a certain percentage of the sampling scheme is not yet well understood and will require future work.

Both the top‐scoring Poisson‐gap sampling scheme (as identified by *NUSscore*), the US‐NUS hybrid sampling schemes with 25%–42.5% uniform sampling and the quantile‐based sampling scheme (equivalent to 40% initial uniform sampling) all provide high‐quality spectra while reducing acquisition time by half with 50% sampling coverage. This reduction in acquisition time comes at a minimal cost, as both sensitivity and resolution are preserved, particularly for the US‐NUS hybrid sampling schemes. The US‐NUS hybrid schemes have been shown to be appropriate for quantitative analysis of spectra with high dynamic range, such as NOESY, but are also expected to generate high‐fidelity HSQC spectra for metabolomic studies where accurate peak areas are important. These hybrid schemes could also be used for routine qualitative work by non‐specialists to accelerate the acquisition of 2D spectra for chemical assignments. The above results were achieved without time equivalent compensation; therefore, in addition to shortening acquisition times, these schemes can be used to enhance resolution or sensitivity by increasing the number of scans or size of the indirect dimension.

The low seed dependence of the US‐NUS hybrid schemes increases the accessibility and generality of their use, as in almost all cases, all seeds performed better than traditional Poisson‐gap sampling schemes. High‐fidelity Poisson‐gap schemes can be generated using the *NUSscore* program and selecting the highest scoring scheme. The US‐NUS generator python script (S16) requires only the desired number of points in the indirect dimension, the sampling coverage, the desired proportion of uniform sampling, and a seed value. These simple parameters should further increase the accessibility of non‐uniform sampling. The quantile‐based sampling schedule is instead seed independent and easily accessible to non‐experts with recommended parameters detailed within the web‐implemented QSched program or within nus‐tool [14, 17].

As accurate interatomic distances derived from quantitative NOESY experiments are highly important for most solution NMR‐based structure determination, the above conclusions are relevant for a wide variety of research fields. Thereto, our findings are expected to be relevant also for quantitative NMR in other contexts, for instance, at the acquisition of quantitative HSQCs for metabolomic studies.

## 5 Experimental

25,000 Poisson‐gap sampling schemes were generated using the program NUSscore (50%; 256 from 512 complex points, SW 10.95, constant time) [16]. A subset containing 11 equally distributed sampling schemes were selected across the whole range of NUSscore values from 0.624 to 1.047. Random‐shuffle sampling schemes were generated from TopSpin (Version 4.4.0) using random seed values from a random number generator. Sampling schemes *SAAR‐best* and *SAAR‐worst* were taken from the library of high‐fidelity seeds as determined by Hyberts et al. [19]. US‐NUS hybrid schedules were generated using in‐house Python scripts (Supporting Information) using seed values 0, 1, and 2. The quantile‐based sampling schedule was generated within *nus‐tool* using a sin‐PDF, 1.5 bias and 2.5 times evolution. Uniformly sampled spectra of spiramycin were converted to the corresponding NUS spectra using in‐house python scripts implemented in TopSpin to acquire NUS spectra with 512 complex points (50% sampling coverage).

Uniformly sampled 2D NOESY spectra of spiramycin in DMSO‐*d*_6_ (4.0 mM) at 25°C were acquired on a 600‐MHz Bruker Avance NEO spectrometer equipped with a 5‐mm TCI cryogenic probe. Seven NOESY experiments were acquired at different mixing times from 100–700 ms using 2048 × 1024 points with four transients per spectrum (pulse program: noesyph).

All spectra were processed and analysed in MestReNova (version 15.0.1) using sine square apodisation, first point 0.5, and zero filling to 2048 in f1, and sine square apodisation in f2. Third‐order polynomial fit baseline correction was used in f2 and f1. Manual phase correction was applied for the US dataset and applied to all subsequent NUS spectra. NUS spectra were reconstructed using the default parameters of the MIST algorithm in static mode, implemented in MestReNova. The absolute intensity of cross‐peaks were obtained by integration. The same processing and integration template was applied to all datasets.

Interproton distances were calculated using NOE build‐up curves from five to seven mixing times, following the removal of outliers, using the initial rate approximation and using two geminal methylene protons (1.78 Å) as a reference distance [24, 25]. Distances were calculated according to Equation (1):

(1) $$r_{\mathrm{AB}}=r_{\mathrm{AB}}\times {\left(\frac{\sigma_{\mathrm{ref}}}{\sigma_{\mathrm{AB}}}\right)}^{\frac{1}{6}}$$

where *r*_*AB*_ is the interproton distance between protons HA and HB, *r*_*ref*_ is 1.78 Å, and *σ*_*ref*_ and *σ*_*AB*_ are the gradients of the build‐up curves for the reference proton pair and proton pair of interest, respectively.

Distances were considered valid, or high quality, if the build‐up curve had an *R* ^2^ > 0.90. Normalised intensities were used in the calculation of the build‐up rate. Normalised intensities *I*_norm_ were derived using the PANIC method [26], in which cross‐peaks are normalised to the diagonal peaks according to Equation (2):

(2) $$I_{\mathrm{norm}}={\left(\frac{{\text{cross peak}}_{\mathrm{AB}}\times {\text{cross peak}}_{\mathrm{BA}}}{{\text{diagonal peak}}_{A}\times {\text{diagonal peak}}_{B}}\right)}^{0.5}$$

Original NMR FIDs of the uniformly sampled NOESY experiments are freely available online at Zenodo with doi: 10.5281/zenodo.14875694.

## Conflicts of Interest

The authors declare no conflicts of interest.

### Peer Review

The peer review history for this article is available at https://www.webofscience.com/api/gateway/wos/peer‐review/10.1002/mrc.5529.

## Supporting information


**Table S1.** Comparison of interproton distance statistics and nus‐tool metrics for the Poisson‐gap sampling schemes (50% total sampling) and random‐shuffle sampling scheme, taking the average results of 15 random‐shuffle schemes.
**Table S2** Comparison of interproton distance statistics and nus‐tool metrics for the US‐NUS hybrid sampling schemes (seeds 0–2) and the average of each three seed values for each proportion of uniform sampling are given in blue.
**Table S3** Random‐shuffle seed values used to generate the averaged random‐shuffle sampling scheme. Sampling schemes were generated in TopSpin 4.4.0 with NUS‐type set to random‐shuffle (‘nussptype 0’).
**Table S4** Interproton distance statistics for the Poisson‐gap sampling schemes with a total sampling of 25%. The disappearance of interproton distances, the number of distances with large errors, and the large average distance deviation deemed 25% coverage too low for quantitative work.
**Figure S1** Comparison of NUS distances against US distances for the NUSscore dataset. The Euclidean norm (L_2_ norm) is given for each NUS scheme as a measure of how far the two datasets deviate, with a lower value representing better agreement between the US and NUS distances. L_2_ norm is positively correlated with the NUSscore rank (*R* ^2^ = 0.758). The identity line is shown in red.
**Figure S2** Comparison of NUS distances against US distances for the US‐NUS hybrid dataset (seed = 0) and quantile sampling scheme. The L_2_ norm is given for each NUS scheme. L_2_ norm is not correlated with the proportion of uniform sampling (*R* ^2^ = 0.184). The identity line is shown in red.
**Figure S3** Comparison of NUS distances against US distances for the SAAR dataset. The L_2_ norm is given for each NUS scheme. The identity line is shown in red.
**Table S5** Poisson‐gap sampling schemes generated and scored using NUSscore, where NUSscore1 and NUSscore11 are the best and worst scoring schemes respectively. Each sampling scheme contains 256 points.
**Table S6** Signal‐to‐artefact apex ratio determined sampling schemes.
**Table S7** US‐NUS hybrid sampling schedules. Sampling schedules are named in the following format as 512‐50‐x‐y (512 points, 50% total sampling, x% US, y = seed).
**Table S8** Interproton distances calculated from NUSscore1–11 sampling schemes as compared to the uniformly sampled reference spectra. NUSscore1 is the highest scoring sampling scheme, and NUSscore11 the lowest. Red distances signify a greater than 7% deviation from the uniformly sampled data. Blue distances signify an interproton distance that was not valid in the uniformly sampled data but is valid in the NUS dataset (*R* ^2^ > 0.90, *n* > 4). Assignments are given as chemical shifts (ppm).
**Table S9** Interproton distances calculated from SAAR‐best and SAAR‐worst sampling schemes as compared to the uniformly sampled reference spectra. Red distances signify a greater than 7% deviation from the uniformly sampled data. Blue distances signify an interproton distance that was not valid in the uniformly sampled data but is valid in the NUS dataset (*R* ^2^ > 0.90, *n* > 4). Assignments are given as chemical shifts (ppm).
**Table S10** Interproton distances calculated from US‐NUS hybrid schemes 512‐50‐25 to 51250‐35 sampling schemes as compared to the uniformly sampled reference spectra. Red distances signify a greater than 7% deviation from the uniformly sampled data. Blue distances signify an interproton distance that was not valid in the uniformly sampled data but were valid in the NUS dataset (R^2^ > 0.90, *n* > 4). Assignments are given as chemical shifts (ppm).
**Table S11** Interproton distances calculated from US‐NUS hybrid schemes 512‐50‐40 to 512‐50‐45 sampling schemes as compared to the uniformly sampled reference spectra. Red distances signify a greater than 7% deviation from the uniformly sampled data. Blue distances signify an interproton distance that was not valid in the uniformly sampled data but were valid in the NUS dataset (*R* ^2^ > 0.90, *n* > 4). Assignments are given as chemical shifts (ppm).
**Table S12** Interproton distances calculated from the quantile‐based sampling schemes as compared to the uniformly sampled reference spectra. Red distances signify a greater than 7% deviation from the uniformly sampled data. Blue distances signify an interproton distance that was not valid in the uniformly sampled data but were valid in the NUS dataset (*R* ^2^ > 0.90, *n* > 4). Assignments are given as chemical shifts (ppm).

## Data Availability

The data that support the findings of this study are openly available in Zenodo at https://zenodo.org/records/14875694, reference number DOI: 10.5281/zenodo.14875694.
