# Supplementary material for: Non‐Uniform Sampling for Quantitative NOESY
Source: Magn Reson Chem. 2025 May 16;63(7):495–507. doi: 10.1002/mrc.5529 (PMC12129645; doi:10.1002/mrc.5529)
Supplement: Supplementary file 1 — Table S1. Comparison of interproton distance statistics and nus‐tool metrics for the Poisson‐gap sampling schemes (50% total sampling) and random‐shuffle sampling scheme, taking the average results of 15 random‐shuffle schemes. Table S2 Comparison of interproton distance statistics and nus‐tool metrics for the US‐NUS hybrid sampling schemes (seeds 0–2) and the average of each three seed values for each proportion of uniform sampling are given in blue. Table S3 Random‐shuffle seed values used to generate the averaged random‐shuffle sampling scheme. Sampling schemes were generated in TopSpin 4.4.0 with NUS‐type set to random‐shuffle (‘nussptype 0’). Table S4 Interproton distance statistics for the Poisson‐gap sampling schemes with a total sampling of 25%. The disappearance of interproton distances, the number of distances with large errors, and the large average distance deviation deemed 25% coverage too low for quantitative work. Figure S1 Comparison of NUS distances against US distances for the NUSscore dataset. The Euclidean norm (L2 norm) is given for each NUS scheme as a measure of how far the two datasets deviate, with a lower value representing better agreement between the US and NUS distances. L2 norm is positively correlated with the NUSscore rank (R 2 = 0.758). The identity line is shown in red. Figure S2 Comparison of NUS distances against US distances for the US‐NUS hybrid dataset (seed = 0) and quantile sampling scheme. The L2 norm is given for each NUS scheme. L2 norm is not correlated with the proportion of uniform sampling (R 2 = 0.184). The identity line is shown in red. Figure S3 Comparison of NUS distances against US distances for the SAAR dataset. The L2 norm is given for each NUS scheme. The identity line is shown in red. Table S5 Poisson‐gap sampling schemes generated and scored using NUSscore, where NUSscore1 and NUSscore11 are the best and worst scoring schemes respectively. Each sampling scheme contains 256 points. Table S6 Signal‐to‐artefact [file MRC-63-495-s001.docx]

**Supporting Information**

**Non-uniform Sampling for Quantitative NOESY**

William T. P. Darling, Sven G. Hyberts, Máté Erdélyi

| **Table of Contents** | S1 |
| --- | --- |
| **S1. Comparison of Interproton Distance Statistics by NUS Scheme** | **S2** |
| Table S1. Poisson-gap and random-shuffle sampling scheme summary | S2 |
| Table S2. US-NUS hybrid sampling scheme summary | S2 |
| Table S3. Random-shuffle sampling scheme summary | S2 |
| Table S4. 25% NUS Poisson-gap sampling scheme summary | S3 |
| **S2. Scatter Plots of NUS and US distances** | S4 |
| Figure S1. NUSscore scatter plots | S6 |
| Figure S2. US-NUS hybrid scatter plots | S8 |
| Figure S3. *SAAR* Poisson-gap scatter plots | S8 |
| **S3. Sampling Schedules** | S9 |
| Table S5. *NUSscore* Poisson-gap sampling schedules | S9 |
| Table S6. *SAAR*-derived sampling schedules | S11 |
| Table S7. US-NUS hybrid sampling schedules | S12 |
| **S4. US-NUS Sampling Scheme Generator Python Code** | **S17** |
| **S5. Tables of interproton distances** | S22 |
| Table S8. Interproton distances of *NUSscore* Poisson-gap sampling schemes | S22 |
| Table S9. Interproton distances of *SAAR* Poisson-gap sampling schemes | S26 |
| Table S10. Interproton distances of US-NUS hybrid schemes: 25-35% US | S30 |
| Table S11. Interproton distances of US-NUS hybrid schemes: 40-45% US | S34 |
| Table S12. Interproton distances of the quantile-based sampling scheme | S38 |

**S1. Comparison of Interproton Distance Statistics by NUS Scheme**

Table S1 Comparison of interproton distance statistics and nus-tool metrics for the Poisson-gap sampling schemes (50% total sampling) and random-shuffle sampling scheme, taking the average results of 15 random-shuffle schemes.

|  | **Total distances** | **Distances with >7% deviation** | **Average distance deviation (%)** | **Peak-to-sidelobe ratio** | **Relative Sensitivity** |
| --- | --- | --- | --- | --- | --- |
| ***US reference*** | 226 | - | - | - | - |
| ***NUSscore1*** | 218 | 3 | 0.55 | 92.2 | 50.7 |
| ***NUSscore2*** | 216 | 6 | 0.53 | 74.2 | 50.5 |
| ***NUSscore3*** | 223 | 8 | 0.49 | 105.3 | 50.7 |
| ***NUSscore4*** | 216 | 5 | 0.70 | 88.6 | 50.8 |
| ***NUSscore5*** | 202 | 10 | 1.20 | 92.8 | 50.5 |
| ***NUSscore6*** | 216 | 7 | 1.50 | 75.5 | 50.5 |
| ***NUSscore7*** | 213 | 4 | 1.32 | 81.6 | 50.7 |
| ***NUSscore8*** | 212 | 10 | 1.44 | 95.4 | 50.3 |
| ***NUSscore9*** | 216 | 16 | 1.81 | 97.1 | 50.5 |
| ***NUSscore10*** | 207 | 11 | 1.43 | 55.8 | 50.3 |
| ***NUSscore11*** | 202 | 15 | 1.81 | 98.7 | 50.3 |
| ***SAAR-best*** | 214 | 7 | 0.96 | 59.9 | 50.6 |
| ***SAAR-worst*** | 219 | 7 | 0.80 | 89.3 | 50.5 |
| ***RS-avg*** | 183.8 | 28 | 3.39 | 86.9 | 50.1 |

Table S2 Comparison of interproton distance statistics and nus-tool metrics for the US-NUS hybrid sampling schemes (seeds 0-2) and the average of each three seed values for each proportion of uniform sampling are given in blue.

|  | **Total distances** | **Distances with >7% deviation** | **Average distance deviation (%)** | **Peak-to-sidelobe ratio** | **Relative Sensitivity** |
| --- | --- | --- | --- | --- | --- |
| ***US reference*** | 226 | - | - | - | - |
| ***512-50-25-0*** | 220 | 3 | -0.26 | 113.6 | 50.6 |
| ***512-50-25-1*** | 222 | 3 | -0.03 | 121.8 | 50.6 |
| ***512-50-25-2*** | 222 | 4 | 0.01 | 156.9 | 50.6 |
| ***512-50-25-avg*** | *221.3* | *3.3* | *-0.093* | *130.8* | *50.6* |
| ***512-50-30-0*** | 221 | 2 | -0.27 | 126.7 | 50.7 |
| ***512-50-30-1*** | 224 | 2 | -0.21 | 143.3 | 50.7 |
| ***512-50-30-2*** | 220 | 4 | -0.10 | 147.7 | 50.7 |
| ***512-50-30-avg*** | *221.7* | *2.7* | *-0.193* | *139.2* | *50.7* |
| ***512-50-35-0*** | 217 | 1 | -0.16 | 119 | 50.8 |
| ***512-50-35-1*** | 224 | 3 | -0.01 | 107.8 | 50.8 |
| ***512-50-35-2*** | 222 | 3 | -0.04 | 175.1 | 50.8 |
| ***512-50-35-avg*** | *221.0* | *2.3* | *-0.07* | *134* | *50.8* |
| ***512-50-40-0*** | 220 | 3 | 0.08 | 56.3 | 50.9 |
| ***512-50-40-1*** | 225 | 1 | -0.11 | 51.8 | 50.9 |
| ***512-50-40-2*** | 221 | 3 | -0.07 | 54.1 | 51.0 |
| ***512-50-40-avg*** | *222.0* | *2.3* | *-0.033* | *54.1* | *50.9* |
| ***512-50-42.5-0*** | 223 | 3 | -0.15 | 42.8 | 51.0 |
| ***512-50-42.5-1*** | 217 | 2 | -0.06 | 40.9 | 51.0 |
| ***512-50-42.5-2*** | 221 | 2 | 0.09 | 36.9 | 51.0 |
| ***512-50-42.5-avg*** | *220.3* | *2.3* | *-0.04* | *40.2* | *51.0* |
| ***512-50-45-0*** | 219 | 1 | 0.03 | 33.5 | 51.0 |
| ***512-50-45-1*** | 222 | 2 | 0.00 | 32.7 | 51.2 |
| ***512-50-45-2*** | 217 | 2 | 0.01 | 33.6 | 51.1 |
| ***512-50-45-avg*** | *219.3* | *1.7* | *0.013* | *33.3* | *51.1* |
| **Quantile** | 222 | 2 | -0.17 | 152.1 | 51.1 |

Table S3: Random-shuffle seed values used to generate the averaged random-shuffle sampling scheme. Sampling schemes were generated in TopSpin 4.4.0 with NUS-type set to random-shuffle (“nussptype 0”).

| **Seed Value** | **Total distances** | **Distances with >7% deviation** | **Average distance deviation (%)** | **Peak-to-sidelobe ratio** | **Relative Sensitivity** |
| --- | --- | --- | --- | --- | --- |
| ***200*** | 180 | 33 | 3.79 | 81.1 | 50.0 |
| ***360*** | 175 | 24 | 3.37 | 91.6 | 50.0 |
| ***575*** | 176 | 36 | 3.47 | 78.0 | 50.0 |
| ***1183*** | 188 | 39 | 4.02 | 94.2 | 50.0 |
| ***1541*** | 181 | 36 | 3.75 | 88.2 | 50.1 |
| ***2296*** | 188 | 21 | 3.06 | 91.5 | 50.0 |
| ***2695*** | 200 | 22 | 3.49 | 76.5 | 50.0 |
| ***3877*** | 183 | 23 | 3.50 | 93.7 | 50.0 |
| ***5550*** | 188 | 33 | 2.96 | 65.6 | 50.0 |
| ***6471*** | 175 | 26 | 3.30 | 90.8 | 50.3 |
| ***7182*** | 188 | 20 | 3.00 | 82.8 | 50.0 |
| ***7485*** | 193 | 18 | 2.34 | 100.5 | 50.2 |
| ***7524*** | 185 | 28 | 3.73 | 96.6 | 50.0 |
| ***7743*** | 182 | 32 | 3.87 | 85.0 | 50.1 |
| ***8405*** | 175 | 29 | 3.18 | 87.9 | 50.2 |
| ***RS-avg*** | 183.8 | 28 | 3.39 | 86.9 | 50.1 |

Table S4 Interproton distance statistics for the Poisson-gap sampling schemes with a total sampling of 25%. The disappearance of interproton distances, the number of distances with large errors, and the large average distance deviation deemed 25% coverage too low for quantitative work.

|  | **Total distances** | **Distances with >7% deviation** | **Average distance deviation (%)** |
| --- | --- | --- | --- |
| ***US reference*** | 226 | - | - |
| ***25%NUSscore1*** | 191 | 26 | 2.90 |
| ***25%NUSscore2*** | 186 | 28 | 3.07 |
| ***25%NUSscore3*** | 177 | 27 | 3.47 |
| ***25%NUSscore4*** | 185 | 36 | 4.03 |
| ***25%NUSscore5*** | 197 | 54 | 4.72 |
| ***25%NUSscore6*** | 194 | 48 | 3.96 |
| ***25%NUSscore7*** | 175 | 34 | 3.86 |
| ***25%NUSscore8*** | 181 | 49 | 5.11 |
| ***25%NUSscore9*** | 165 | 50 | 5.19 |
| ***25%NUSscore10*** | 183 | 45 | 4.76 |
| ***25%NUSscore11*** | 197 | 58 | 5.23 |
| ***25%SAAR-best*** | 172 | 45 | 5.03 |
| ***25%SAAR-worst*** | 191 | 31 | 4.21 |

**S2. Scatter Plots of NUS and US distances**

*
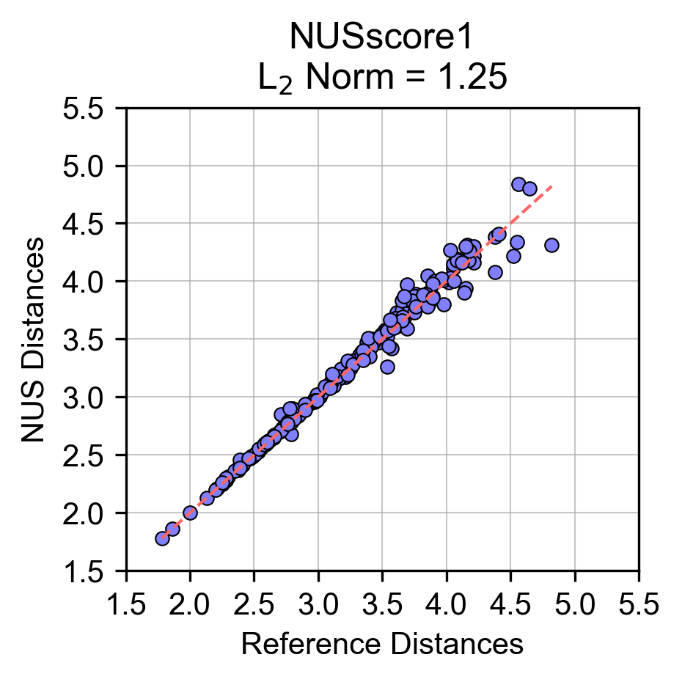
*
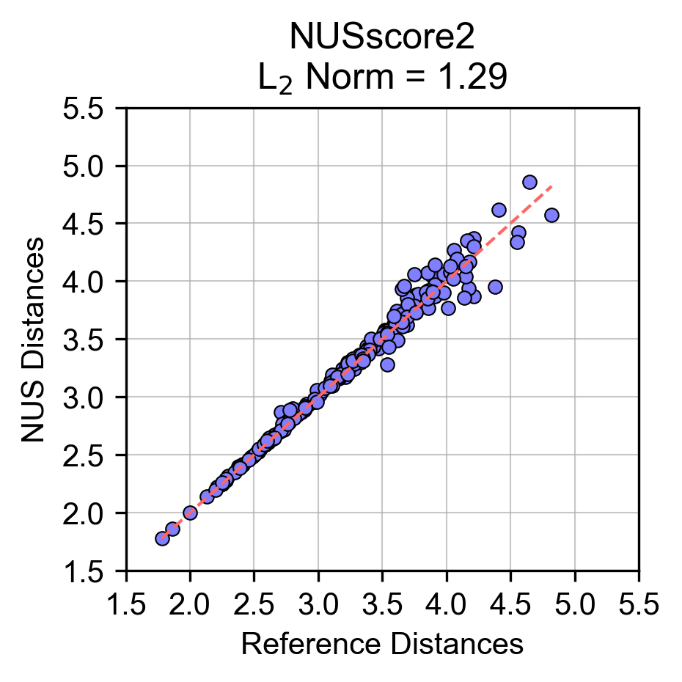


*
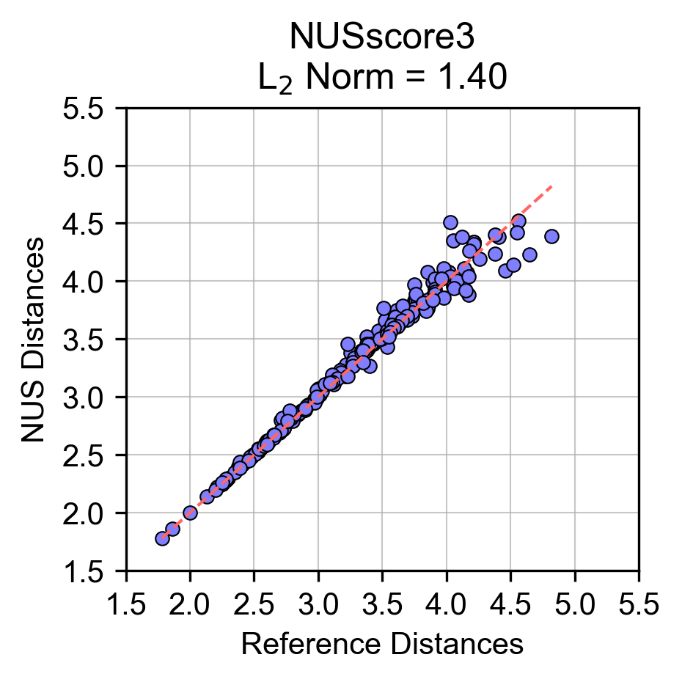

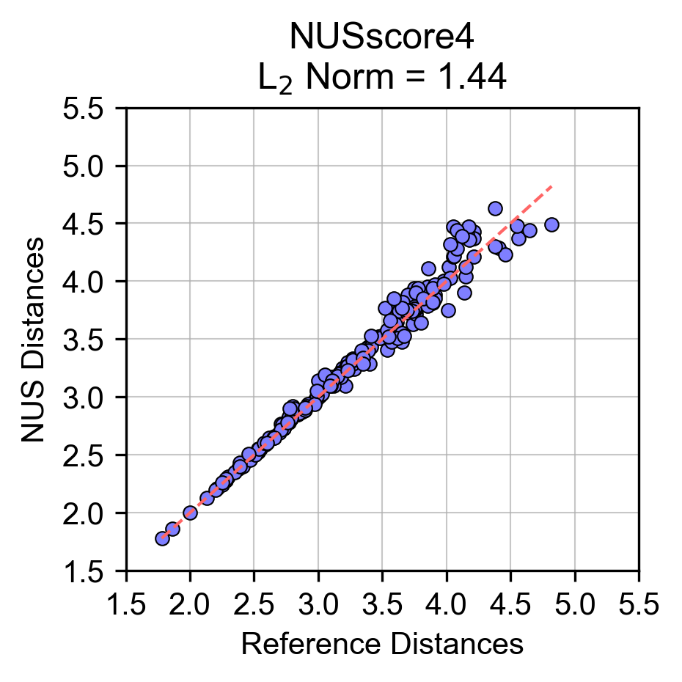
*

*
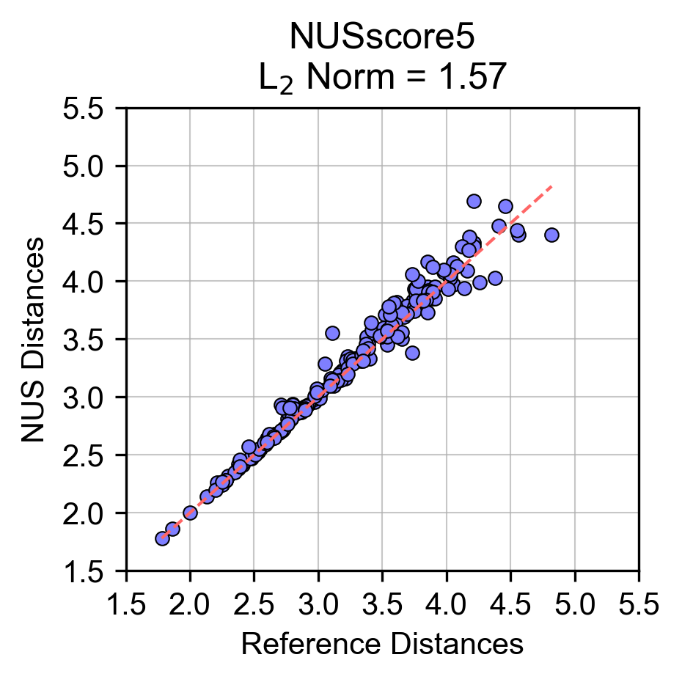

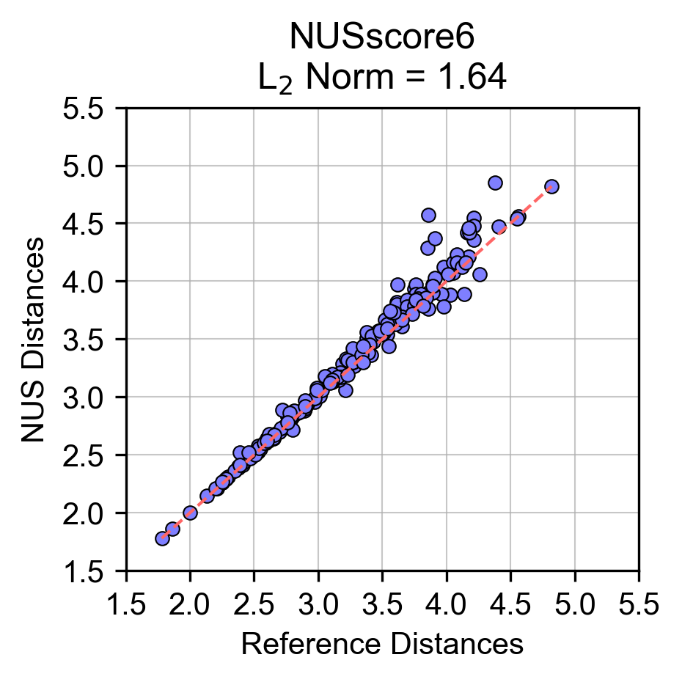
*

*
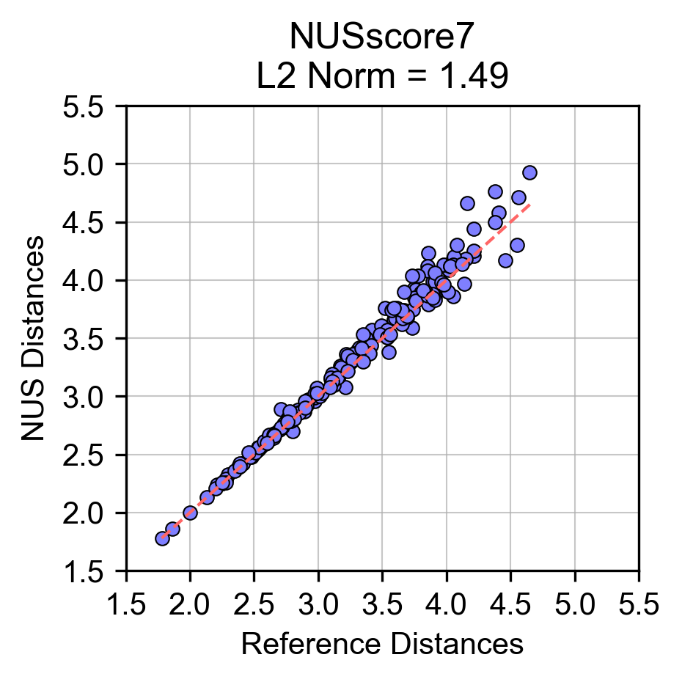

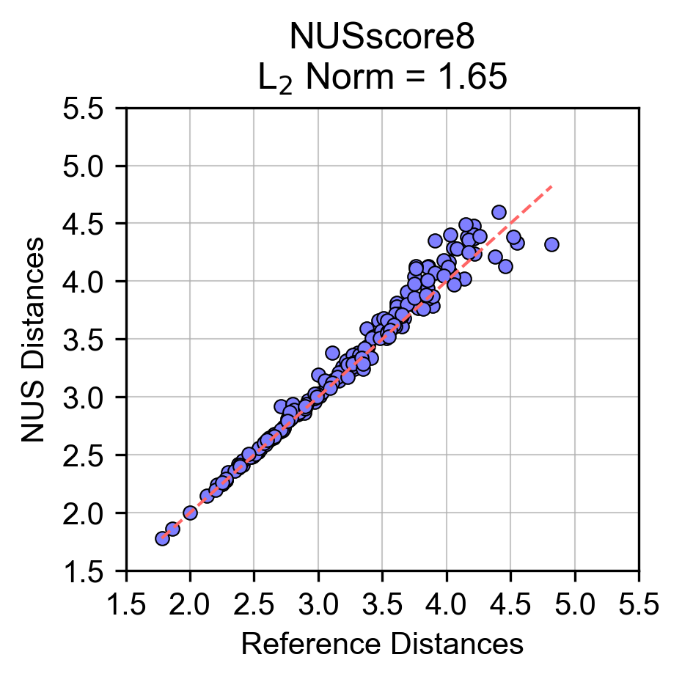
*

*
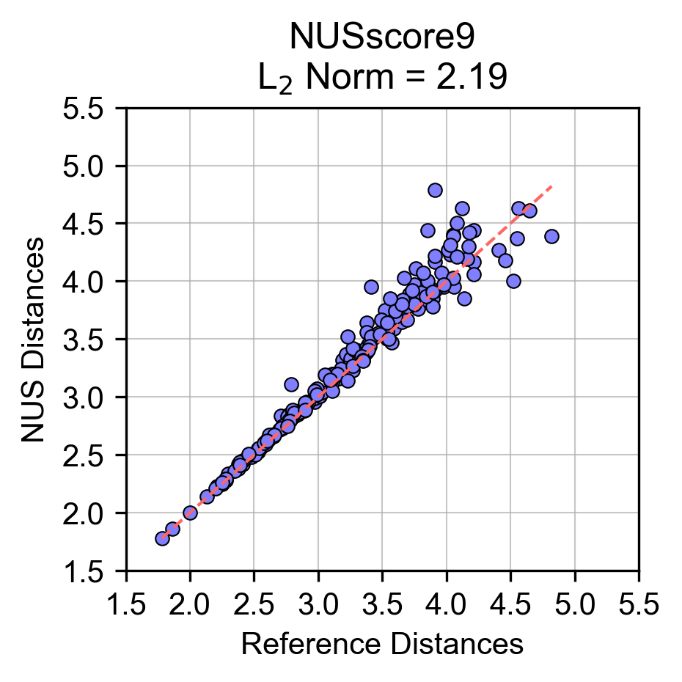

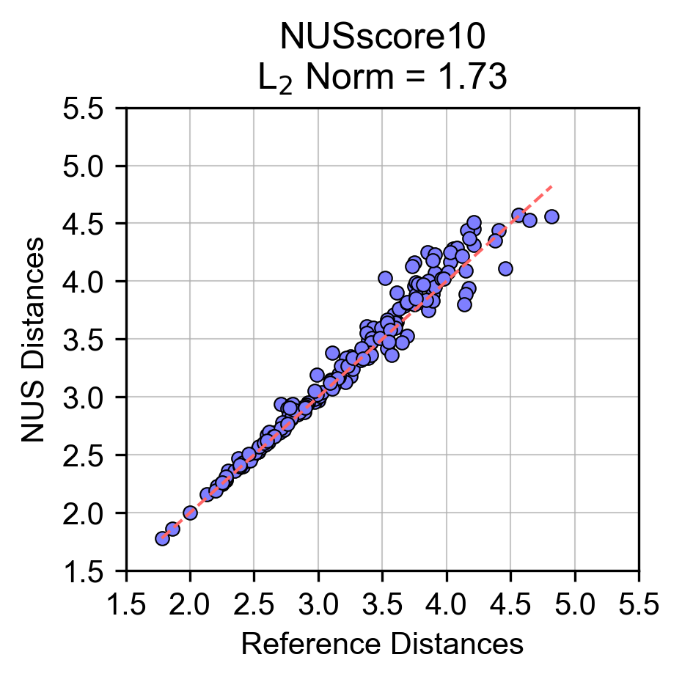
*

*
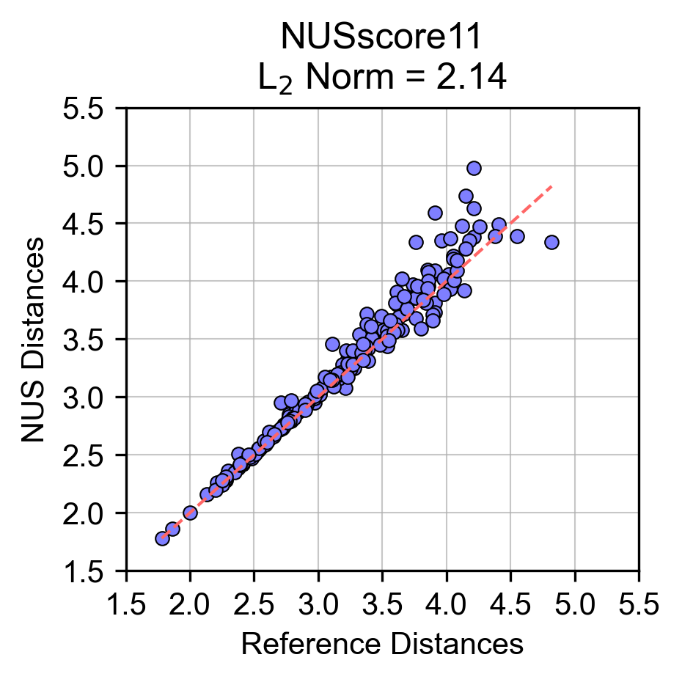
*

Figures S1 Comparison of NUS distances against US distances for the NUSscore dataset. The Euclidean norm (L_2_ norm) is given for each NUS scheme as a measure of how far the two datasets deviate, with a lower value representing better agreement between the US and NUS distances. L_2_ norm is positively correlated with the NUSscore rank (R^2^ = 0.758). The identity line is shown in red.


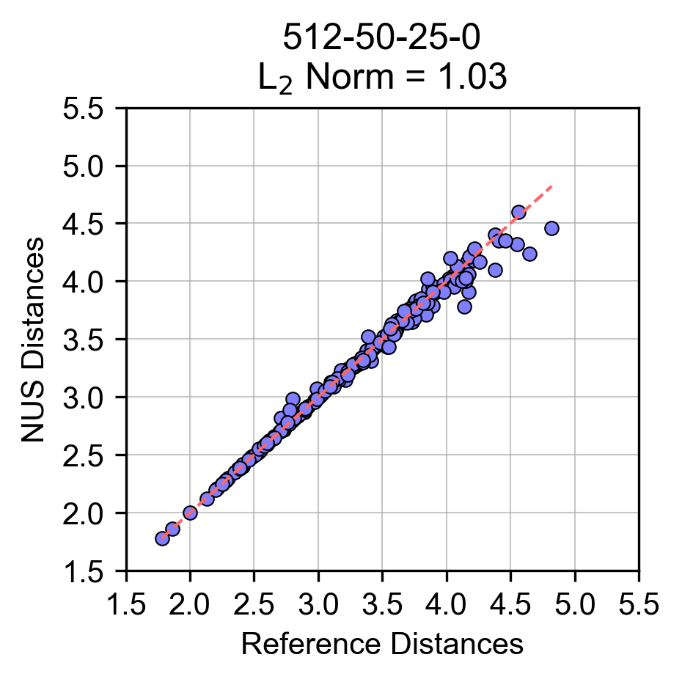

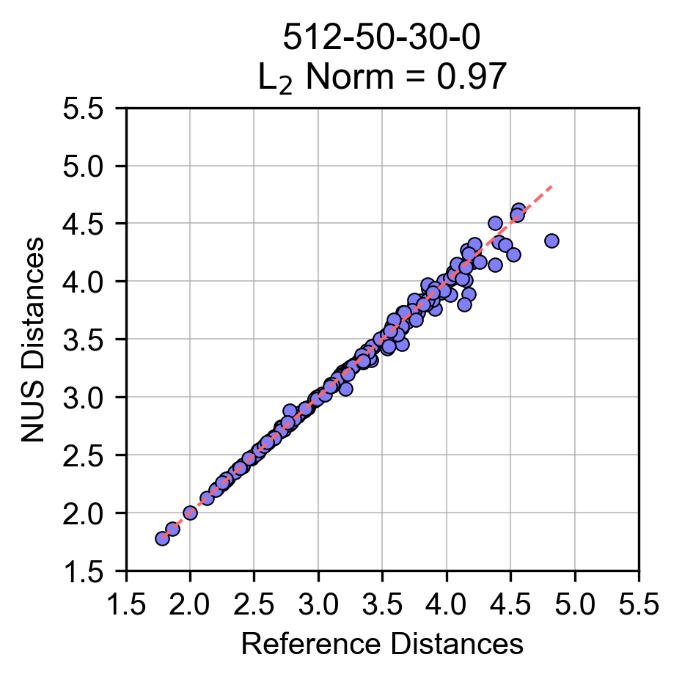


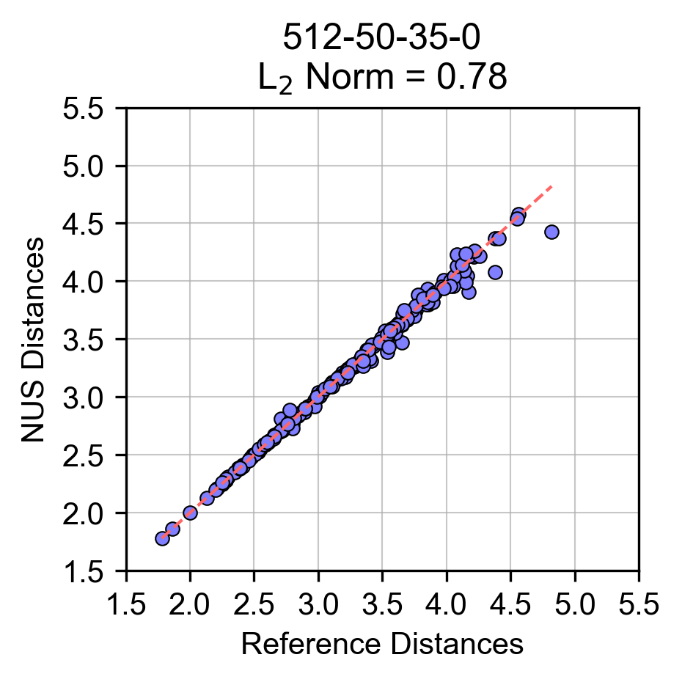

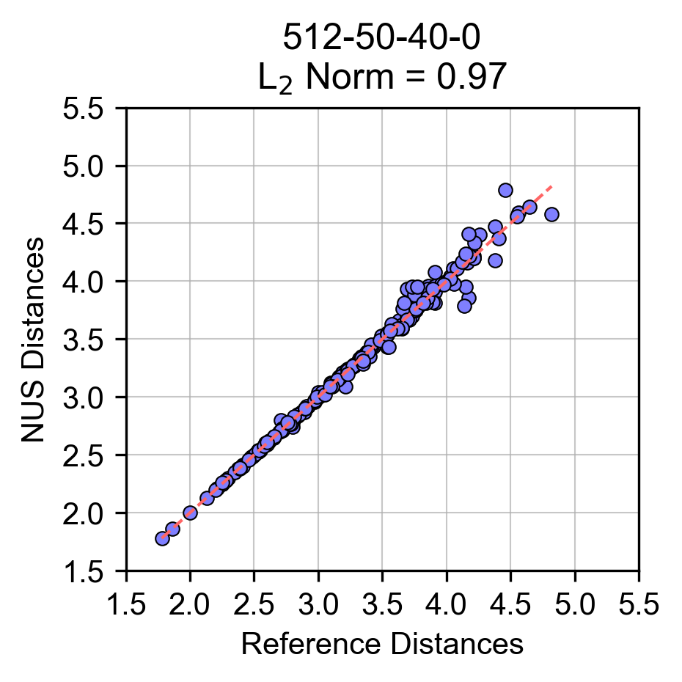

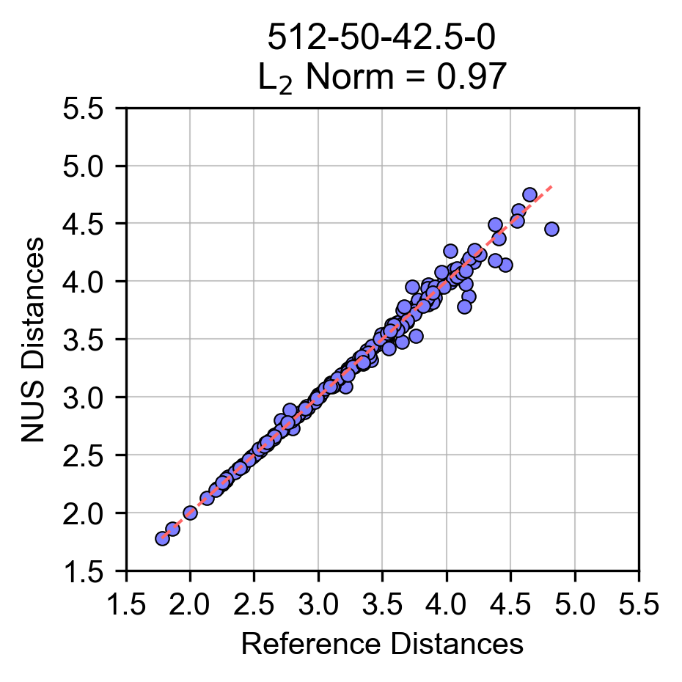

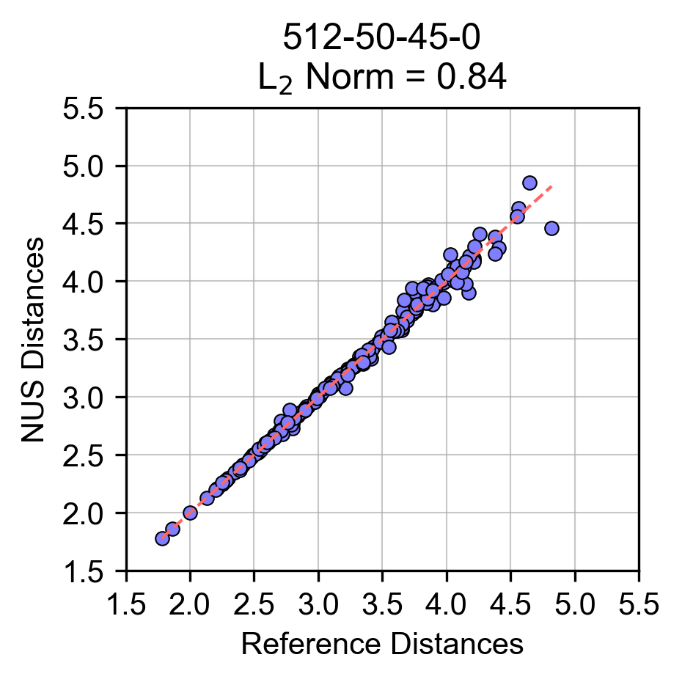


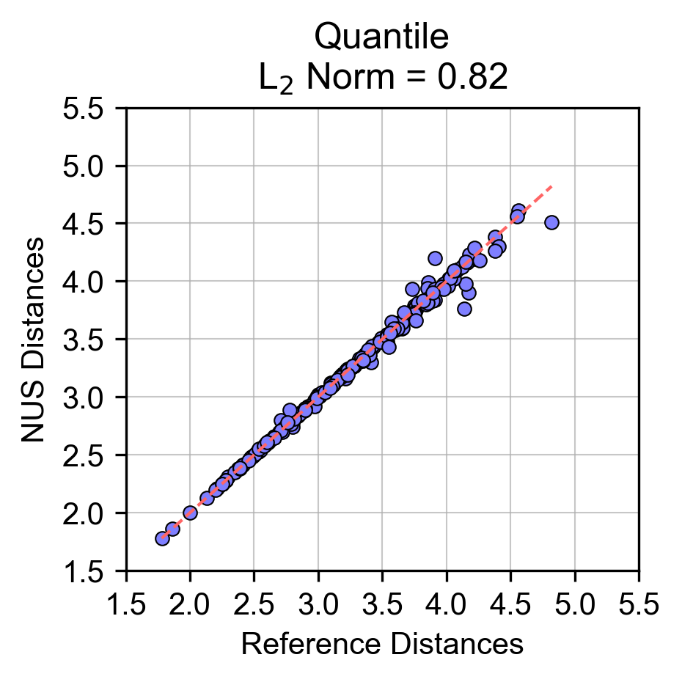


Figures S2 Comparison of NUS distances against US distances for the US-NUS hybrid dataset (seed = 0) and quantile sampling scheme. The L_2_ norm is given for each NUS scheme. L_2_ norm is not correlated with the proportion of uniform sampling (R^2^ = 0.184). The identity line is shown in red.

*
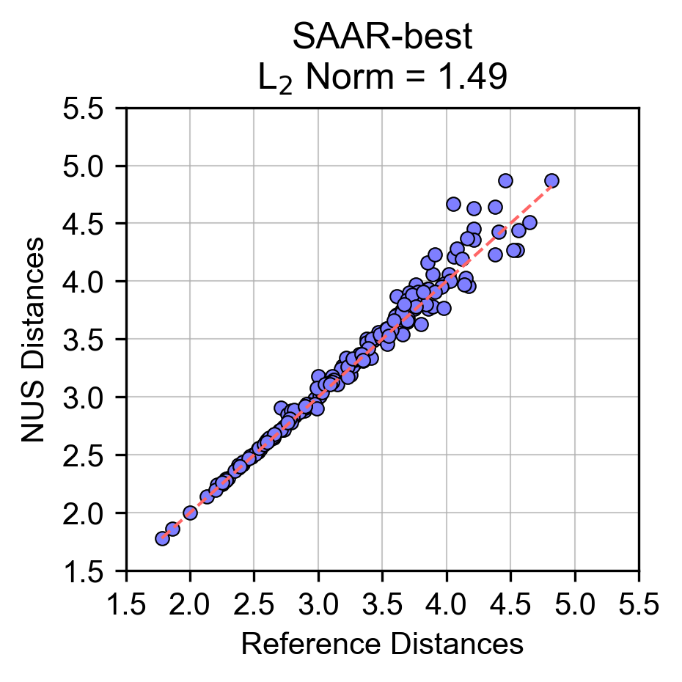
*
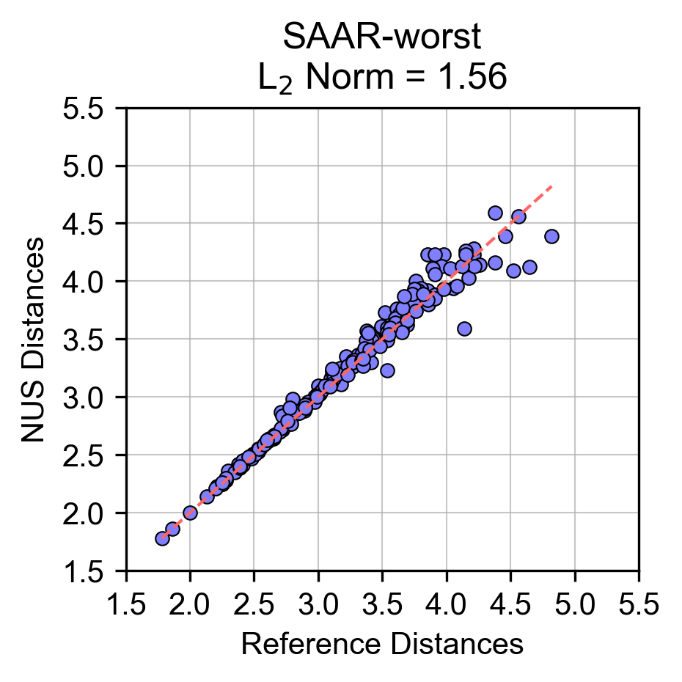


Figures S3 Comparison of NUS distances against US distances for the SAAR dataset. The L_2_ norm is given for each NUS scheme. The identity line is shown in red.

**S3. Sampling Schedules**

Table S5: Poisson-gap sampling schemes generated and scored using NUSscore, where NUSscore1 and NUSscore11 are the best and worst scoring schemes respectively. Each sampling scheme contains 256 points.

| *NUSscore1* | 0 1 2 3 4 5 6 7 8 9 10 11 12 13 14 15 16 17 18 19 20 21 22 23 25 26 27 28 29 30 31 32 33 34 35 36 37 38 39 40 41 42 43 44 45 46 47 49 50 51 52 53 55 56 57 58 59 60 61 63 64 65 66 67 68 69 70 72 73 74 75 76 77 78 79 80 81 83 84 85 86 87 88 89 90 91 92 93 95 96 97 98 101 102 104 106 107 108 109 111 114 115 116 119 120 122 123 124 127 129 132 133 134 137 138 139 140 141 143 144 145 147 148 149 150 153 154 157 158 161 162 165 167 168 169 171 172 176 180 181 184 185 186 188 191 193 195 198 201 203 204 207 212 216 220 224 225 231 233 237 242 246 250 252 254 255 257 262 264 267 271 274 276 278 280 281 285 287 289 295 297 299 302 305 307 310 312 316 320 326 329 331 335 337 340 343 348 350 356 357 360 361 365 368 372 375 377 381 384 389 390 391 395 398 400 405 407 410 411 415 420 424 427 429 430 433 436 437 439 442 447 449 451 454 455 457 460 462 463 465 469 473 476 479 481 484 485 486 491 493 494 496 500 505 506 509 |
| --- | --- |
| *NUSscore2* | 0 1 2 3 4 5 6 7 8 9 10 11 12 13 14 15 16 17 18 19 20 21 22 23 24 25 26 27 28 29 30 31 32 33 34 35 36 37 38 39 40 41 42 43 44 45 47 48 49 50 52 54 55 56 57 58 59 60 61 62 63 64 65 66 67 69 70 71 72 73 75 77 78 80 81 83 85 86 89 90 91 92 94 95 96 97 98 99 100 102 103 104 106 107 108 110 111 113 116 117 120 121 122 123 124 125 127 128 131 132 134 135 138 140 142 143 144 147 150 151 153 154 155 157 158 160 161 162 163 164 167 169 171 174 175 176 179 180 184 187 188 192 193 196 199 201 203 206 211 212 213 214 218 220 222 225 227 231 234 238 241 243 247 248 252 253 258 259 260 266 268 273 275 279 282 283 286 287 289 293 296 297 298 300 304 305 306 308 312 315 317 319 321 326 327 330 333 336 341 342 344 347 350 352 355 359 360 362 366 367 368 372 374 377 383 387 392 398 401 403 405 411 415 416 418 424 427 431 433 437 439 442 446 448 455 459 460 465 466 469 471 474 477 478 481 482 485 489 492 495 498 500 504 505 507 511 |
| *NUSscore3* | 0 1 2 3 4 5 6 7 8 9 10 11 12 13 14 15 17 18 19 20 21 22 23 24 25 26 27 28 29 30 31 32 33 34 35 36 37 38 39 40 41 42 43 44 45 46 47 48 49 50 51 53 54 56 57 58 59 61 62 64 66 67 68 69 71 72 73 75 77 78 79 80 82 83 85 87 88 89 90 91 93 94 95 96 97 98 99 100 102 103 105 106 107 109 111 113 115 117 118 119 120 121 122 124 125 129 131 133 134 136 137 139 141 146 147 150 151 152 153 155 156 159 161 162 165 166 167 169 170 173 174 178 180 183 184 187 189 190 194 195 197 199 201 203 205 206 208 209 210 213 214 216 219 221 225 227 229 233 237 242 244 246 249 251 252 256 259 261 263 266 269 271 275 276 281 282 285 287 289 291 293 295 296 297 299 301 303 308 312 314 316 319 322 324 325 330 333 338 339 340 343 349 352 354 358 361 364 366 368 374 376 381 385 391 395 398 401 404 409 414 416 417 421 424 425 428 430 432 434 439 442 443 444 447 451 455 459 460 461 463 464 467 471 474 476 480 483 488 494 498 499 502 504 505 506 509 |
| *NUSscore4* | 0 1 2 3 4 5 6 7 8 9 10 11 12 13 14 15 16 17 18 19 20 21 22 23 24 26 27 29 30 31 32 34 35 36 37 38 39 40 41 42 43 44 46 47 48 49 50 51 52 53 54 55 56 57 58 60 61 62 63 64 65 66 67 68 69 70 71 73 76 77 78 80 82 84 85 86 87 88 89 91 92 94 95 97 99 100 103 105 107 108 110 111 113 115 116 117 119 121 123 125 126 129 130 132 133 135 137 138 139 141 142 143 144 145 147 148 149 151 154 159 160 162 164 165 167 169 171 172 173 174 175 176 177 179 180 181 182 185 186 187 188 192 195 197 201 202 203 204 206 208 210 211 213 215 216 218 220 224 227 231 234 235 236 237 239 240 242 244 247 249 251 253 254 258 264 266 269 273 276 279 282 284 288 291 293 296 297 300 302 305 307 310 311 314 318 320 323 326 329 332 334 337 338 340 344 348 350 352 354 356 358 359 361 366 370 371 372 375 377 385 389 391 394 399 403 408 412 417 423 424 425 428 432 436 441 444 446 450 453 456 458 463 468 473 477 481 484 488 489 490 493 496 500 503 505 508 |
| *NUSscore5* | 0 1 2 3 4 5 6 7 8 9 10 11 12 13 14 15 16 17 18 19 20 21 22 23 24 26 27 28 29 30 31 32 33 34 35 36 37 38 40 41 42 44 47 48 51 52 53 55 56 58 59 60 61 62 63 65 66 67 68 69 70 71 73 76 77 78 79 80 81 82 83 85 87 88 90 91 94 95 97 98 99 100 102 103 106 107 109 110 111 113 114 117 118 121 122 124 127 128 129 132 133 134 136 138 139 141 142 143 144 145 148 149 151 152 153 155 159 161 163 164 166 168 170 172 175 176 178 179 184 185 187 191 193 194 197 198 200 202 203 204 207 210 213 216 218 222 223 226 228 230 231 233 235 237 239 242 243 244 245 247 248 250 253 257 259 260 263 266 268 270 272 275 277 280 281 285 287 289 292 296 298 301 307 312 318 322 323 327 328 329 331 336 338 341 343 344 346 351 353 355 358 361 365 366 369 370 374 375 379 380 384 385 386 387 391 394 400 403 407 408 410 412 413 417 422 425 428 431 434 438 441 442 445 448 450 453 457 459 460 462 463 465 470 473 477 480 483 487 490 492 497 502 505 506 509 510 |
| *NUSscore6* | 0 1 2 3 4 5 6 7 8 9 11 12 13 14 15 16 17 18 19 21 22 23 24 26 27 28 30 31 32 33 34 35 36 38 39 40 41 42 44 46 47 48 49 50 51 53 54 55 59 60 61 62 63 64 65 66 67 68 69 71 73 74 75 78 79 80 81 82 83 84 85 86 88 89 91 92 94 95 96 97 98 100 103 105 107 109 110 112 113 114 115 116 118 120 121 123 124 127 128 129 131 133 134 135 136 139 142 143 146 147 150 152 155 157 160 161 164 166 168 170 172 174 177 179 181 183 185 187 189 190 192 194 196 197 198 199 200 201 203 206 208 210 212 214 216 218 219 224 227 229 231 234 237 239 242 244 246 247 250 251 254 257 260 261 262 264 267 268 270 272 275 278 280 283 288 290 293 295 298 299 303 306 309 313 315 320 322 325 327 328 330 332 334 338 339 340 344 346 350 352 355 358 361 365 368 371 378 381 384 387 388 392 394 397 400 403 405 409 411 412 415 418 420 426 430 431 440 442 443 447 448 451 452 455 456 458 460 462 463 464 468 470 473 477 483 486 490 492 495 497 500 502 503 505 507 510 |
| *NUSscore7* | 0 1 2 3 4 5 6 7 8 9 10 11 12 13 14 15 16 17 18 19 20 21 22 23 24 25 27 28 29 30 32 33 35 36 37 39 41 42 44 45 46 47 48 49 50 51 54 55 58 59 60 61 63 65 66 68 70 73 74 76 77 78 79 80 81 84 85 87 89 91 93 94 96 97 100 101 102 104 106 107 108 109 110 112 113 115 116 117 120 121 123 124 125 126 127 128 132 133 134 135 136 138 140 142 144 147 149 152 154 158 159 161 163 165 168 171 172 174 175 176 178 179 180 184 186 187 188 189 191 194 199 202 203 208 209 213 214 217 218 219 222 223 224 228 229 232 234 236 237 240 241 242 244 246 250 253 256 258 260 264 268 271 273 277 279 280 281 282 285 287 288 291 293 295 296 298 300 302 307 309 310 312 315 320 321 324 327 329 330 332 334 335 338 341 345 347 350 351 354 356 358 360 362 363 365 369 370 371 372 375 379 381 384 387 390 393 396 398 399 401 403 408 409 411 419 423 427 430 434 437 440 443 448 450 452 454 457 459 463 466 468 470 471 475 478 482 484 486 487 491 492 495 499 500 505 508 |
| *NUSscore8* | 0 1 2 3 4 5 6 7 8 9 10 11 12 13 14 16 17 18 19 20 21 22 23 24 25 26 27 28 29 31 33 35 36 37 38 39 40 41 42 43 45 47 48 49 52 56 57 59 60 61 64 66 67 69 71 72 73 74 76 78 81 82 83 84 86 87 88 89 91 93 95 97 98 100 101 102 104 106 108 111 112 113 116 117 118 121 123 125 126 128 129 130 132 134 136 138 140 142 144 145 148 149 151 154 155 156 158 161 162 163 165 166 168 169 172 173 175 176 179 180 182 184 187 191 194 195 197 200 201 203 204 205 207 208 210 211 215 217 220 223 226 229 231 232 235 237 240 244 245 247 248 251 253 255 256 258 259 261 264 266 269 271 273 276 277 279 282 285 289 296 299 301 305 307 311 314 318 321 325 327 330 331 334 335 337 338 343 345 347 348 351 357 358 360 362 363 364 367 369 371 373 375 378 382 385 387 392 394 395 401 404 406 407 408 411 413 416 419 425 426 429 431 433 435 438 440 443 446 449 451 452 453 457 459 462 464 467 469 470 476 480 481 482 484 486 488 489 491 493 496 500 503 506 508 510 511 |
| *NUSscore9* | 0 1 2 3 4 5 6 7 8 9 11 12 13 14 15 17 18 19 20 21 22 23 24 25 26 28 30 31 32 33 34 36 39 40 41 43 44 45 46 47 49 51 52 53 55 57 58 59 60 61 64 65 67 70 72 73 76 78 79 80 81 82 83 85 86 88 90 91 93 94 96 97 98 99 101 102 104 105 106 109 111 112 114 118 120 121 124 125 126 127 128 130 132 135 136 137 138 139 142 143 145 147 148 150 153 155 157 159 162 163 167 169 171 174 176 179 180 183 185 189 193 194 196 197 198 200 201 204 205 207 208 212 213 216 217 218 219 220 221 223 224 227 229 232 235 237 238 242 245 246 250 252 255 256 258 264 266 268 270 274 277 279 280 283 286 290 292 295 296 299 302 304 307 309 310 312 313 314 318 321 323 326 330 332 335 336 338 341 342 343 347 350 353 355 356 358 360 362 364 368 372 373 374 376 379 383 386 388 391 393 394 396 398 404 406 408 410 411 414 416 418 422 424 425 429 434 435 438 442 443 445 450 452 454 457 458 460 462 467 472 474 475 476 479 483 487 489 492 493 496 497 499 501 503 506 509 |
| *NUSscore10* | 0 1 2 3 4 5 6 7 9 10 11 12 14 15 16 17 18 19 20 22 23 24 26 28 29 30 31 32 34 35 36 37 38 39 40 41 42 43 44 46 48 51 52 54 56 57 58 61 62 63 64 66 69 71 73 75 76 77 78 79 82 84 85 86 88 92 93 94 97 99 101 102 103 105 107 108 109 111 113 114 115 116 118 122 123 124 125 128 131 135 137 142 144 145 146 148 149 151 154 155 157 158 160 163 164 166 168 170 172 174 177 179 182 186 187 189 190 192 193 194 196 197 199 202 204 206 210 213 215 217 219 220 221 224 225 227 229 230 233 237 239 241 242 244 245 247 248 250 251 254 256 258 260 262 265 267 268 269 271 273 275 278 279 281 282 286 288 291 294 298 299 300 304 307 308 311 313 315 321 323 326 327 331 334 336 339 341 342 344 347 348 351 355 356 358 362 363 369 374 376 377 385 386 389 390 393 395 397 399 400 405 408 409 412 413 416 419 422 424 428 431 432 435 436 437 442 444 445 447 450 451 453 456 457 460 464 466 470 472 476 479 481 483 485 486 489 490 491 493 495 499 501 504 507 509 511 |
| *NUSscore11* | 0 1 2 3 4 6 7 8 10 11 12 14 16 17 19 20 21 22 23 25 26 27 28 29 30 31 32 33 34 35 37 38 40 41 43 45 47 48 49 51 52 54 55 58 59 60 62 63 64 65 68 69 70 72 73 75 78 80 81 82 83 84 87 88 91 93 95 96 97 98 99 100 101 102 103 106 107 108 110 112 114 116 118 119 120 123 124 127 128 129 132 134 136 138 141 143 146 149 152 153 154 156 159 160 164 168 170 173 175 179 181 183 185 187 190 191 192 194 195 196 198 202 203 204 208 212 214 216 217 219 222 223 225 226 227 229 231 233 236 238 239 241 245 248 250 251 254 256 258 260 261 263 265 266 268 270 273 276 282 284 285 287 288 290 292 293 296 300 302 304 309 311 312 314 315 319 323 327 328 331 332 333 335 336 337 340 342 346 349 350 353 356 359 360 362 364 365 366 367 370 374 375 378 379 381 383 386 388 392 393 396 397 399 400 402 405 407 410 412 414 416 423 424 427 431 436 437 441 443 445 448 450 454 456 458 461 462 464 467 472 474 477 481 482 484 486 489 493 498 499 500 502 504 507 509 511 |

Table S6: Signal-to-artifact apex ratio determined sampling schemes.

| *SAAR-best* | 0 1 2 3 4 5 6 7 8 9 10 11 12 13 14 16 17 18 19 21 22 23 24 25 26 27 28 29 30 31 32 33 34 35 37 38 39 40 41 42 43 44 45 46 47 49 50 53 54 56 58 59 61 62 63 64 65 66 67 68 70 71 72 74 77 79 81 82 83 84 87 90 93 94 95 96 97 99 100 101 103 105 106 108 109 111 113 114 116 119 121 122 124 127 129 130 132 133 135 137 138 139 140 141 143 146 147 149 151 153 156 157 158 163 165 166 168 170 171 173 174 175 177 179 180 181 182 184 186 188 189 190 193 195 200 202 203 208 212 216 219 222 223 225 226 229 233 235 236 241 242 246 247 249 251 255 257 262 263 264 268 269 271 273 274 276 280 282 285 287 289 292 296 299 300 304 305 309 311 312 315 316 317 323 325 328 332 335 336 339 341 345 348 351 352 354 355 357 361 364 366 371 373 376 379 380 384 385 388 390 393 396 400 402 404 408 409 411 414 417 422 427 428 429 430 435 436 440 444 445 446 451 454 456 460 461 463 466 469 471 475 477 480 483 484 486 489 491 492 493 496 500 501 505 507 509 |
| --- | --- |
| *SAAR-worst* | 0 1 3 4 5 6 7 9 10 11 12 13 14 15 16 18 19 20 21 22 23 24 25 26 27 28 29 30 31 33 35 36 37 38 39 40 41 42 44 45 46 47 48 50 51 52 53 54 56 57 59 60 62 63 65 66 69 70 72 73 74 76 77 78 79 80 81 82 83 85 86 87 88 89 90 92 93 94 95 96 98 99 100 101 102 103 104 105 110 111 112 115 118 120 122 123 125 128 130 135 137 139 140 144 145 146 147 150 151 153 156 158 159 161 163 166 168 172 177 178 179 182 184 187 189 190 193 194 195 197 202 203 204 205 206 209 210 211 212 215 218 219 221 222 223 224 225 228 231 233 235 237 238 239 241 243 246 247 249 250 252 255 257 258 263 265 269 270 272 274 275 277 280 283 287 289 290 293 296 297 298 300 302 306 309 312 317 320 322 325 328 331 332 336 338 341 346 348 351 353 357 362 363 366 369 371 372 375 376 380 383 388 391 394 395 399 403 404 407 411 413 417 420 422 423 427 430 432 434 437 439 441 442 444 447 451 454 457 459 462 464 467 470 472 474 479 481 482 487 490 493 497 500 503 507 510 |

Table S7: US-NUS hybrid sampling schedules. Sampling schedules are named in the following format as 512-50-x-y (512 points, 50% total sampling, x% US, y = seed).

| *512-50-25-0* | 0 1 2 3 4 5 6 7 8 9 10 11 12 13 14 15 16 17 18 19 20 21 22 23 24 25 26 27 28 29 30 31 32 33 34 35 36 37 38 39 40 41 42 43 44 45 46 47 48 49 50 51 52 53 54 55 56 57 58 59 60 61 62 63 64 65 66 67 68 69 70 71 72 73 74 75 76 77 78 79 80 81 82 83 84 85 86 87 88 89 90 91 92 93 94 95 96 97 98 99 100 101 102 103 104 105 106 107 108 109 110 111 112 113 114 115 116 117 118 119 120 121 122 123 124 125 126 127 129 130 131 132 133 134 135 137 138 139 140 142 143 144 146 147 148 149 151 153 155 157 158 161 165 167 170 172 173 174 175 178 179 182 188 192 196 200 202 205 207 209 214 217 220 226 230 235 239 243 246 248 252 254 259 265 271 276 281 284 288 291 294 297 307 313 317 323 327 334 339 345 352 358 362 366 371 374 377 382 389 393 395 400 408 415 417 419 420 426 427 433 439 443 450 451 454 461 464 467 469 472 473 477 480 482 485 487 488 489 490 491 492 494 496 497 499 500 501 502 503 504 505 507 508 509 510 511 |
| --- | --- |
| *512-50-25-1* | 0 1 2 3 4 5 6 7 8 9 10 11 12 13 14 15 16 17 18 19 20 21 22 23 24 25 26 27 28 29 30 31 32 33 34 35 36 37 38 39 40 41 42 43 44 45 46 47 48 49 50 51 52 53 54 55 56 57 58 59 60 61 62 63 64 65 66 67 68 69 70 71 72 73 74 75 76 77 78 79 80 81 82 83 84 85 86 87 88 89 90 91 92 93 94 95 96 97 98 99 100 101 102 103 104 105 106 107 108 109 110 111 112 113 114 115 116 117 118 119 120 121 122 123 124 125 126 127 129 130 131 132 134 135 136 137 139 140 142 143 144 145 147 149 152 154 156 157 159 161 162 163 165 167 168 170 171 172 175 176 179 181 184 186 188 191 194 198 203 205 208 211 214 219 222 227 229 233 238 242 245 248 251 254 260 267 273 277 287 293 295 296 304 308 315 319 322 327 330 333 339 344 348 350 356 361 366 370 376 382 386 387 395 399 404 408 411 417 426 432 433 435 439 443 445 450 457 460 462 465 467 469 471 472 474 475 480 482 485 487 488 489 490 493 494 496 498 501 503 505 506 507 508 509 510 511 |
| *512-50-25-2* | 0 1 2 3 4 5 6 7 8 9 10 11 12 13 14 15 16 17 18 19 20 21 22 23 24 25 26 27 28 29 30 31 32 33 34 35 36 37 38 39 40 41 42 43 44 45 46 47 48 49 50 51 52 53 54 55 56 57 58 59 60 61 62 63 64 65 66 67 68 69 70 71 72 73 74 75 76 77 78 79 80 81 82 83 84 85 86 87 88 89 90 91 92 93 94 95 96 97 98 99 100 101 102 103 104 105 106 107 108 109 110 111 112 113 114 115 116 117 118 119 120 121 122 123 124 125 126 127 129 130 131 132 133 135 136 138 140 142 144 145 146 147 148 150 152 154 155 156 159 160 161 164 167 169 170 173 175 177 182 183 188 193 194 198 200 201 203 207 212 217 219 221 226 233 236 239 242 250 252 260 264 267 271 275 278 282 283 286 293 295 303 309 316 319 325 330 336 344 350 357 363 367 375 382 387 392 395 398 401 402 407 413 419 423 426 428 433 438 442 443 446 448 453 455 457 460 464 466 471 473 475 478 479 480 482 484 486 488 489 491 494 495 496 498 499 500 501 502 503 504 505 507 508 509 510 511 |
| *512-50-30-0* | 0 1 2 3 4 5 6 7 8 9 10 11 12 13 14 15 16 17 18 19 20 21 22 23 24 25 26 27 28 29 30 31 32 33 34 35 36 37 38 39 40 41 42 43 44 45 46 47 48 49 50 51 52 53 54 55 56 57 58 59 60 61 62 63 64 65 66 67 68 69 70 71 72 73 74 75 76 77 78 79 80 81 82 83 84 85 86 87 88 89 90 91 92 93 94 95 96 97 98 99 100 101 102 103 104 105 106 107 108 109 110 111 112 113 114 115 116 117 118 119 120 121 122 123 124 125 126 127 128 129 130 131 132 133 134 135 136 137 138 139 140 141 142 143 144 145 146 147 148 149 150 151 152 153 155 156 158 159 160 161 162 163 164 165 166 168 170 172 173 174 176 178 180 182 185 188 192 197 198 199 202 205 207 211 215 218 224 228 232 234 238 245 251 258 267 273 277 284 289 297 301 305 312 317 320 324 333 337 342 348 350 353 357 361 365 367 372 380 387 391 400 408 415 422 428 433 442 448 449 453 456 460 467 469 472 474 477 479 480 483 484 487 488 489 490 492 495 498 502 503 505 506 508 509 510 511 |
| *512-50-30-1* | 0 1 2 3 4 5 6 7 8 9 10 11 12 13 14 15 16 17 18 19 20 21 22 23 24 25 26 27 28 29 30 31 32 33 34 35 36 37 38 39 40 41 42 43 44 45 46 47 48 49 50 51 52 53 54 55 56 57 58 59 60 61 62 63 64 65 66 67 68 69 70 71 72 73 74 75 76 77 78 79 80 81 82 83 84 85 86 87 88 89 90 91 92 93 94 95 96 97 98 99 100 101 102 103 104 105 106 107 108 109 110 111 112 113 114 115 116 117 118 119 120 121 122 123 124 125 126 127 128 129 130 131 132 133 134 135 136 137 138 139 140 141 142 143 144 145 146 147 148 149 150 151 152 153 155 156 157 158 159 160 161 162 164 166 168 170 172 174 176 179 180 182 183 185 186 188 190 191 194 199 202 205 209 213 215 220 221 228 234 236 238 239 241 244 251 256 265 271 275 282 285 290 299 306 308 313 319 326 336 347 354 357 365 371 376 383 388 391 398 402 406 411 415 421 425 432 435 441 444 449 453 454 456 460 465 470 471 473 476 478 482 484 486 489 491 492 493 497 499 501 502 504 505 506 509 511 |
| *512-50-30-2* | 0 1 2 3 4 5 6 7 8 9 10 11 12 13 14 15 16 17 18 19 20 21 22 23 24 25 26 27 28 29 30 31 32 33 34 35 36 37 38 39 40 41 42 43 44 45 46 47 48 49 50 51 52 53 54 55 56 57 58 59 60 61 62 63 64 65 66 67 68 69 70 71 72 73 74 75 76 77 78 79 80 81 82 83 84 85 86 87 88 89 90 91 92 93 94 95 96 97 98 99 100 101 102 103 104 105 106 107 108 109 110 111 112 113 114 115 116 117 118 119 120 121 122 123 124 125 126 127 128 129 130 131 132 133 134 135 136 137 138 139 140 141 142 143 144 145 146 147 148 149 150 151 152 153 155 156 157 158 159 160 161 162 164 166 167 168 170 171 173 175 177 179 181 182 186 190 191 192 194 198 201 203 207 210 214 217 222 232 235 237 241 246 253 255 260 268 272 279 283 293 297 302 309 315 322 326 333 339 343 350 353 362 371 377 382 386 392 397 401 404 411 417 421 426 430 437 440 445 449 452 457 461 463 467 470 474 475 479 481 483 485 488 490 491 494 495 497 498 501 502 504 506 507 509 510 511 |
| *512-50-35-0* | 0 1 2 3 4 5 6 7 8 9 10 11 12 13 14 15 16 17 18 19 20 21 22 23 24 25 26 27 28 29 30 31 32 33 34 35 36 37 38 39 40 41 42 43 44 45 46 47 48 49 50 51 52 53 54 55 56 57 58 59 60 61 62 63 64 65 66 67 68 69 70 71 72 73 74 75 76 77 78 79 80 81 82 83 84 85 86 87 88 89 90 91 92 93 94 95 96 97 98 99 100 101 102 103 104 105 106 107 108 109 110 111 112 113 114 115 116 117 118 119 120 121 122 123 124 125 126 127 128 129 130 131 132 133 134 135 136 137 138 139 140 141 142 143 144 145 146 147 148 149 150 151 152 153 154 155 156 157 158 159 160 161 162 163 164 165 166 167 168 169 170 171 172 173 174 175 176 177 178 180 181 182 183 185 187 188 189 190 193 194 197 199 200 203 208 212 213 215 222 224 228 236 238 246 252 264 267 271 277 280 289 294 300 307 314 319 325 332 342 348 361 368 374 384 390 400 408 414 424 431 434 443 450 451 461 463 468 472 476 479 482 484 487 490 492 493 495 496 499 501 505 506 508 509 510 511 |
| *512-50-35-1* | 0 1 2 3 4 5 6 7 8 9 10 11 12 13 14 15 16 17 18 19 20 21 22 23 24 25 26 27 28 29 30 31 32 33 34 35 36 37 38 39 40 41 42 43 44 45 46 47 48 49 50 51 52 53 54 55 56 57 58 59 60 61 62 63 64 65 66 67 68 69 70 71 72 73 74 75 76 77 78 79 80 81 82 83 84 85 86 87 88 89 90 91 92 93 94 95 96 97 98 99 100 101 102 103 104 105 106 107 108 109 110 111 112 113 114 115 116 117 118 119 120 121 122 123 124 125 126 127 128 129 130 131 132 133 134 135 136 137 138 139 140 141 142 143 144 145 146 147 148 149 150 151 152 153 154 155 156 157 158 159 160 161 162 163 164 165 166 167 168 169 170 171 172 173 174 175 176 177 178 180 181 183 184 186 187 188 191 193 196 198 202 204 208 212 214 218 220 223 228 233 236 238 242 246 250 259 267 277 282 286 288 294 303 308 316 325 335 339 348 352 365 373 376 379 390 397 401 409 414 417 424 430 434 441 446 453 456 459 462 465 470 474 478 482 486 490 492 495 496 498 502 506 508 509 510 511 |
| *512-50-35-2* | 0 1 2 3 4 5 6 7 8 9 10 11 12 13 14 15 16 17 18 19 20 21 22 23 24 25 26 27 28 29 30 31 32 33 34 35 36 37 38 39 40 41 42 43 44 45 46 47 48 49 50 51 52 53 54 55 56 57 58 59 60 61 62 63 64 65 66 67 68 69 70 71 72 73 74 75 76 77 78 79 80 81 82 83 84 85 86 87 88 89 90 91 92 93 94 95 96 97 98 99 100 101 102 103 104 105 106 107 108 109 110 111 112 113 114 115 116 117 118 119 120 121 122 123 124 125 126 127 128 129 130 131 132 133 134 135 136 137 138 139 140 141 142 143 144 145 146 147 148 149 150 151 152 153 154 155 156 157 158 159 160 161 162 163 164 165 166 167 168 169 170 171 172 173 174 175 176 177 178 180 181 183 184 186 188 191 192 196 197 198 203 205 207 210 214 215 217 220 221 224 228 233 240 246 249 255 265 272 274 282 293 298 303 307 317 324 329 335 338 345 349 354 360 367 374 385 393 398 409 418 423 430 436 440 444 452 457 462 465 467 469 476 481 484 488 489 493 494 496 499 501 502 503 505 508 511 |
| *512-50-40-0* | 0 1 2 3 4 5 6 7 8 9 10 11 12 13 14 15 16 17 18 19 20 21 22 23 24 25 26 27 28 29 30 31 32 33 34 35 36 37 38 39 40 41 42 43 44 45 46 47 48 49 50 51 52 53 54 55 56 57 58 59 60 61 62 63 64 65 66 67 68 69 70 71 72 73 74 75 76 77 78 79 80 81 82 83 84 85 86 87 88 89 90 91 92 93 94 95 96 97 98 99 100 101 102 103 104 105 106 107 108 109 110 111 112 113 114 115 116 117 118 119 120 121 122 123 124 125 126 127 128 129 130 131 132 133 134 135 136 137 138 139 140 141 142 143 144 145 146 147 148 149 150 151 152 153 154 155 156 157 158 159 160 161 162 163 164 165 166 167 168 169 170 171 172 173 174 175 176 177 178 179 180 181 182 183 184 185 186 187 188 189 190 191 192 193 194 195 196 197 198 199 200 201 202 203 204 206 207 210 211 212 213 215 216 219 222 227 231 235 239 243 246 251 258 262 270 275 287 303 310 322 336 348 361 377 389 395 404 417 424 438 447 454 462 469 476 483 489 492 495 498 504 506 507 508 510 511 |
| *512-50-40-1* | 0 1 2 3 4 5 6 7 8 9 10 11 12 13 14 15 16 17 18 19 20 21 22 23 24 25 26 27 28 29 30 31 32 33 34 35 36 37 38 39 40 41 42 43 44 45 46 47 48 49 50 51 52 53 54 55 56 57 58 59 60 61 62 63 64 65 66 67 68 69 70 71 72 73 74 75 76 77 78 79 80 81 82 83 84 85 86 87 88 89 90 91 92 93 94 95 96 97 98 99 100 101 102 103 104 105 106 107 108 109 110 111 112 113 114 115 116 117 118 119 120 121 122 123 124 125 126 127 128 129 130 131 132 133 134 135 136 137 138 139 140 141 142 143 144 145 146 147 148 149 150 151 152 153 154 155 156 157 158 159 160 161 162 163 164 165 166 167 168 169 170 171 172 173 174 175 176 177 178 179 180 181 182 183 184 185 186 187 188 189 190 191 192 193 194 195 196 197 198 199 200 201 202 203 204 206 207 208 210 212 213 217 219 223 224 230 236 245 250 255 263 270 280 286 293 302 311 323 337 351 357 372 382 395 406 423 435 448 454 462 470 474 478 485 487 491 494 498 502 504 505 506 508 509 510 511 |
| *512-50-40-2* | 0 1 2 3 4 5 6 7 8 9 10 11 12 13 14 15 16 17 18 19 20 21 22 23 24 25 26 27 28 29 30 31 32 33 34 35 36 37 38 39 40 41 42 43 44 45 46 47 48 49 50 51 52 53 54 55 56 57 58 59 60 61 62 63 64 65 66 67 68 69 70 71 72 73 74 75 76 77 78 79 80 81 82 83 84 85 86 87 88 89 90 91 92 93 94 95 96 97 98 99 100 101 102 103 104 105 106 107 108 109 110 111 112 113 114 115 116 117 118 119 120 121 122 123 124 125 126 127 128 129 130 131 132 133 134 135 136 137 138 139 140 141 142 143 144 145 146 147 148 149 150 151 152 153 154 155 156 157 158 159 160 161 162 163 164 165 166 167 168 169 170 171 172 173 174 175 176 177 178 179 180 181 182 183 184 185 186 187 188 189 190 191 192 193 194 195 196 197 198 199 200 201 202 203 204 206 207 209 210 211 213 214 215 216 219 222 223 225 228 233 239 244 252 257 261 269 277 288 293 309 320 332 347 361 380 393 409 421 434 445 453 463 466 473 478 484 488 495 497 501 503 504 505 506 509 511 |
| *512-50-42.5-0* | 0 1 2 3 4 5 6 7 8 9 10 11 12 13 14 15 16 17 18 19 20 21 22 23 24 25 26 27 28 29 30 31 32 33 34 35 36 37 38 39 40 41 42 43 44 45 46 47 48 49 50 51 52 53 54 55 56 57 58 59 60 61 62 63 64 65 66 67 68 69 70 71 72 73 74 75 76 77 78 79 80 81 82 83 84 85 86 87 88 89 90 91 92 93 94 95 96 97 98 99 100 101 102 103 104 105 106 107 108 109 110 111 112 113 114 115 116 117 118 119 120 121 122 123 124 125 126 127 128 129 130 131 132 133 134 135 136 137 138 139 140 141 142 143 144 145 146 147 148 149 150 151 152 153 154 155 156 157 158 159 160 161 162 163 164 165 166 167 168 169 170 171 172 173 174 175 176 177 178 179 180 181 182 183 184 185 186 187 188 189 190 191 192 193 194 195 196 197 198 199 200 201 202 203 204 205 206 207 208 209 210 211 212 213 214 215 216 217 219 220 221 223 227 233 236 240 243 247 257 264 267 274 279 292 301 315 327 344 363 380 399 410 427 447 458 470 476 484 490 498 502 504 506 509 510 511 |
| *512-50-42.5-1* | 0 1 2 3 4 5 6 7 8 9 10 11 12 13 14 15 16 17 18 19 20 21 22 23 24 25 26 27 28 29 30 31 32 33 34 35 36 37 38 39 40 41 42 43 44 45 46 47 48 49 50 51 52 53 54 55 56 57 58 59 60 61 62 63 64 65 66 67 68 69 70 71 72 73 74 75 76 77 78 79 80 81 82 83 84 85 86 87 88 89 90 91 92 93 94 95 96 97 98 99 100 101 102 103 104 105 106 107 108 109 110 111 112 113 114 115 116 117 118 119 120 121 122 123 124 125 126 127 128 129 130 131 132 133 134 135 136 137 138 139 140 141 142 143 144 145 146 147 148 149 150 151 152 153 154 155 156 157 158 159 160 161 162 163 164 165 166 167 168 169 170 171 172 173 174 175 176 177 178 179 180 181 182 183 184 185 186 187 188 189 190 191 192 193 194 195 196 197 198 199 200 201 202 203 204 205 206 207 208 209 210 211 212 213 214 215 216 217 219 220 222 224 228 230 233 237 242 246 252 262 270 273 282 293 310 322 338 352 367 383 398 416 424 439 451 461 468 484 490 498 500 503 506 508 510 511 |
| *512-50-42.5-2* | 0 1 2 3 4 5 6 7 8 9 10 11 12 13 14 15 16 17 18 19 20 21 22 23 24 25 26 27 28 29 30 31 32 33 34 35 36 37 38 39 40 41 42 43 44 45 46 47 48 49 50 51 52 53 54 55 56 57 58 59 60 61 62 63 64 65 66 67 68 69 70 71 72 73 74 75 76 77 78 79 80 81 82 83 84 85 86 87 88 89 90 91 92 93 94 95 96 97 98 99 100 101 102 103 104 105 106 107 108 109 110 111 112 113 114 115 116 117 118 119 120 121 122 123 124 125 126 127 128 129 130 131 132 133 134 135 136 137 138 139 140 141 142 143 144 145 146 147 148 149 150 151 152 153 154 155 156 157 158 159 160 161 162 163 164 165 166 167 168 169 170 171 172 173 174 175 176 177 178 179 180 181 182 183 184 185 186 187 188 189 190 191 192 193 194 195 196 197 198 199 200 201 202 203 204 205 206 207 208 209 210 211 212 213 214 215 216 217 219 220 221 222 226 229 233 237 240 246 253 261 267 275 286 301 316 335 346 359 376 386 399 415 431 438 459 469 483 492 495 498 499 501 505 508 510 511 |
| *512-50-45-0* | 0 1 2 3 4 5 6 7 8 9 10 11 12 13 14 15 16 17 18 19 20 21 22 23 24 25 26 27 28 29 30 31 32 33 34 35 36 37 38 39 40 41 42 43 44 45 46 47 48 49 50 51 52 53 54 55 56 57 58 59 60 61 62 63 64 65 66 67 68 69 70 71 72 73 74 75 76 77 78 79 80 81 82 83 84 85 86 87 88 89 90 91 92 93 94 95 96 97 98 99 100 101 102 103 104 105 106 107 108 109 110 111 112 113 114 115 116 117 118 119 120 121 122 123 124 125 126 127 128 129 130 131 132 133 134 135 136 137 138 139 140 141 142 143 144 145 146 147 148 149 150 151 152 153 154 155 156 157 158 159 160 161 162 163 164 165 166 167 168 169 170 171 172 173 174 175 176 177 178 179 180 181 182 183 184 185 186 187 188 189 190 191 192 193 194 195 196 197 198 199 200 201 202 203 204 205 206 207 208 209 210 211 212 213 214 215 216 217 218 219 220 221 222 223 224 225 226 227 228 229 231 232 233 235 240 243 249 255 261 267 279 290 309 332 367 394 417 438 455 473 482 490 500 502 507 511 |
| *512-50-45-1* | 0 1 2 3 4 5 6 7 8 9 10 11 12 13 14 15 16 17 18 19 20 21 22 23 24 25 26 27 28 29 30 31 32 33 34 35 36 37 38 39 40 41 42 43 44 45 46 47 48 49 50 51 52 53 54 55 56 57 58 59 60 61 62 63 64 65 66 67 68 69 70 71 72 73 74 75 76 77 78 79 80 81 82 83 84 85 86 87 88 89 90 91 92 93 94 95 96 97 98 99 100 101 102 103 104 105 106 107 108 109 110 111 112 113 114 115 116 117 118 119 120 121 122 123 124 125 126 127 128 129 130 131 132 133 134 135 136 137 138 139 140 141 142 143 144 145 146 147 148 149 150 151 152 153 154 155 156 157 158 159 160 161 162 163 164 165 166 167 168 169 170 171 172 173 174 175 176 177 178 179 180 181 182 183 184 185 186 187 188 189 190 191 192 193 194 195 196 197 198 199 200 201 202 203 204 205 206 207 208 209 210 211 212 213 214 215 216 217 218 219 220 221 222 223 224 225 226 227 228 229 231 232 233 236 238 241 242 245 247 255 263 270 282 294 310 328 358 388 422 448 471 483 493 497 506 510 |
| *512-50-45-2* | 0 1 2 3 4 5 6 7 8 9 10 11 12 13 14 15 16 17 18 19 20 21 22 23 24 25 26 27 28 29 30 31 32 33 34 35 36 37 38 39 40 41 42 43 44 45 46 47 48 49 50 51 52 53 54 55 56 57 58 59 60 61 62 63 64 65 66 67 68 69 70 71 72 73 74 75 76 77 78 79 80 81 82 83 84 85 86 87 88 89 90 91 92 93 94 95 96 97 98 99 100 101 102 103 104 105 106 107 108 109 110 111 112 113 114 115 116 117 118 119 120 121 122 123 124 125 126 127 128 129 130 131 132 133 134 135 136 137 138 139 140 141 142 143 144 145 146 147 148 149 150 151 152 153 154 155 156 157 158 159 160 161 162 163 164 165 166 167 168 169 170 171 172 173 174 175 176 177 178 179 180 181 182 183 184 185 186 187 188 189 190 191 192 193 194 195 196 197 198 199 200 201 202 203 204 205 206 207 208 209 210 211 212 213 214 215 216 217 218 219 220 221 222 223 224 225 226 227 228 229 231 232 235 239 243 250 254 260 268 277 291 303 317 338 362 394 420 448 470 477 491 497 501 505 509 511 |
| *Quantile* | 0 1 2 3 4 5 6 7 8 9 10 11 12 13 14 15 16 17 18 19 20 21 22 23 24 25 26 27 28 29 30 31 32 33 34 35 36 37 38 39 40 41 42 43 44 45 46 47 48 49 50 51 52 53 54 55 56 57 58 59 60 61 62 63 64 65 66 67 68 69 70 71 72 73 74 75 76 77 78 79 80 81 82 83 84 85 86 87 88 89 90 91 92 93 94 95 96 97 98 99 100 101 102 103 104 105 106 107 108 109 110 111 112 113 114 115 116 117 118 119 120 121 122 123 124 125 126 127 128 129 130 131 132 133 134 135 136 137 138 139 140 141 142 143 144 145 146 147 148 149 150 151 152 153 154 155 156 157 158 159 160 161 162 163 164 165 166 167 168 169 170 171 172 173 174 175 176 177 178 179 180 181 182 183 184 185 186 187 188 189 190 191 192 193 194 195 196 197 198 199 200 201 202 203 204 205 206 208 210 212 214 217 219 221 223 226 228 230 233 236 238 241 243 246 249 252 255 258 261 264 268 271 275 278 282 286 290 294 298 303 308 313 318 324 330 336 343 351 359 368 379 391 406 424 451 511 |

**S4. US-NUS Sampling Scheme Generator Python Code**

Python script for generating US-NUS hybrid schemes

Output can be saved straight to clipboard by setting enable_clipboard_copying to True.

import math

import random

# Set to True to enable clipboard copying

enable_clipboard_copying = False

if enable_clipboard_copying:

import pyperclip

#####################################

## ##

## US/NUS PG GENERATOR ##

## ##

#####################################

def poisson(lmbd):

"""

Generate a Poisson random number using the Knuth algorithm.

"""

L = math.exp(-lmbd)

k = 0

p = 1

while p >= L:

u = random.random() # Uniform random number in [0, 1)

p *= u

k += 1

return k - 1

def poisson_gap_sampling(seed, sampling_points, total_range_size):

"""

Perform Poisson-gap sampling to generate non-uniformly sampled points.

"""

# Initialize parameters

ld = total_range_size / sampling_points # Mean gap

w = 2.0 # Initial weight

sampled_points = []

# Seed the random number generator

random.seed(seed)

while True:

i = 0

k = 0

temp_points = []

while i < total_range_size:

temp_points.append(i)

i += 1

k += 1

# Generate a gap using the Poisson distribution

gap = poisson((ld - 1.0) * w * math.sin((i / (total_range_size + 1)) * math.pi))

i += gap

if k > sampling_points:

w *= 1.02 # Too many points, increase weight

elif k < sampling_points:

w /= 1.02 # Too few points, decrease weight

else:

sampled_points = temp_points

break

return sampled_points[:sampling_points]

# Main function

def main():

# Step 1: Get inputs

total_points = int(input("Indirect dimension number of points? ")) # e.g., 512

sampling_density = float(input("Sampling density (between 0 and 1)? ")) # e.g., 0.5

max_forward_weighting = sampling_density # Maximum allowed forward weighting

forward_weighting = float(input(f"Forward weighting (between 0 and {max_forward_weighting})? ")) # e.g., 0.4

# Step 2: Choose a seed

seed_input = input("Choose a seed (leave blank for a random seed): ")

if seed_input.strip() == "":

seed = random.randint(0, 999999)

else:

seed = int(seed_input)

# Step 3: Ask whether to save as a text file

save_choice = input("Do you want to save the list as a text file? (y/n): ").strip().lower()

# Step 4: Calculate forward-weighted points

forward_weighted_points = round(total_points * forward_weighting)

# Step 5: Calculate remaining points to sample

target_sample_size = round(total_points * sampling_density)

remaining_points_to_sample = target_sample_size - forward_weighted_points

# Step 6: Poisson-gap sampling for the remaining points

total_range_size = total_points - forward_weighted_points - 1

if remaining_points_to_sample > 0 and total_range_size > 0:

poisson_sampled_points = poisson_gap_sampling(seed, remaining_points_to_sample, total_range_size)

# Adjust points to start from the end of forward-weighted list

poisson_sampled_points = [point + forward_weighted_points + 1 for point in poisson_sampled_points]

else:

poisson_sampled_points = []

# Combine both lists

final_list = sorted(forward_list + poisson_sampled_points)

# Step 7: Display the result

print("\nGenerated List:")

for num in final_list:

print(num)

# Step 8: Copy the final list to clipboard if enabled

list_as_string = "\n".join(map(str, final_list))

if enable_clipboard_copying:

pyperclip.copy(list_as_string)

# Print seed info

print(f"\nThe list has been generated using seed {seed}.")

# Step 9: Save if chosen

if save_choice == "y":

filename = f"{total_points}-{int(sampling_density * 100)}-{int(forward_weighting * 100)}-{seed}.txt"

with open(filename, "w") as file:

file.write(list_as_string)

print(f"List saved as {filename}")

# Run the script

if __name__ == "__main__":

main()

**S5. Tables of interproton distances**

Table S8: Interproton distances calculated from NUSscore1-11 sampling schemes as compared to the uniformly sampled reference spectra. NUSscore1 is the highest scoring sampling scheme, and NUSscore11 the lowest. Red distances signify a greater than 7% deviation from the uniformly sampled data. Blue distances signify an interproton distance that was not valid in the uniformly sampled data but is valid in the NUS dataset (R^2^ > 0.90, n > 4). Assignments are given as chemical shifts (ppm).

| f2 δ  (ppm) | f1 δ  (ppm) | Ref.  r (Å) | NUS-score1  r (Å) | NUS-score2  r (Å) | NUS-score3  r (Å) | NUS-score4  r (Å) | NUS-score5  r (Å) | NUS-score6  r (Å) | NUS-score7  r (Å) | NUS-score8  r (Å) | NUS-score9  r (Å) | NUS-score10  r (Å) | NUS-score11  r (Å) |
| --- | --- | --- | --- | --- | --- | --- | --- | --- | --- | --- | --- | --- | --- |
| **9.7** | **6.21** | 4.56 | 4.84 | 4.42 | 4.52 | 4.37 | 4.4 | 4.56 | 4.71 |  | 4.63 | 4.57 |  |
| **9.7** | **4.5** | 4.21 | 4.22 | 4.37 | 4.34 | 4.43 | 4.33 | 4.55 | 4.44 | 4.37 | 4.17 | 4.31 | 4.38 |
| **9.7** | **4.38** | 4.21 | 4.16 | 3.87 | 4.34 | 4.37 | 4.69 | 4.36 | 4.21 | 4.48 | 4.06 | 4.45 | 4.63 |
| **9.7** | **4.42** |  |  |  |  |  |  |  |  | **5.72** |  |  |  |
| **9.7** | **4.02** | 4.16 | 4.31 | 4.35 | 4.07 | 4.4 | 4.09 | 4.42 | 4.66 | 4.38 | 4.19 | 4.44 |  |
| **9.7** | **3.84** | 3.01 | 3.01 | 3.03 | 3.02 | 3.01 | 3.02 | 3.02 | 3.03 | 3.03 | 3.01 | 3.03 | 3.03 |
| **9.7** | **3.65** | 4.38 | 4.38 |  |  | 4.63 |  | 4.85 | 4.76 |  |  |  |  |
| **9.7** | **3.35** | 3.86 |  |  |  | 4.11 |  | 4.57 | 4.23 | 4.13 | 3.93 |  | 3.96 |
| **9.7** | **3.14** |  |  |  | **4.77** |  |  |  |  |  |  |  |  |
| **9.7** | **3.08** |  |  |  | **5.26** |  |  |  |  |  |  |  |  |
| **9.7** | **2.67** | 3.23 | 3.28 | 3.3 | 3.27 | 3.3 | 3.35 | 3.32 | 3.33 | 3.32 | 3.31 | 3.31 | 3.33 |
| **9.7** | **2.22** | 3.19 | 3.24 | 3.24 | 3.24 | 3.25 | 3.23 | 3.29 | 3.27 | 3.26 | 3.32 | 3.27 | 3.28 |
| **9.7** | **2.17** | 3 |  |  | 3.07 | 3.14 |  |  |  | 3.19 |  | 2.97 |  |
| **9.7** | **1.91** | 3.75 | 3.86 | 3.84 | 3.83 | 3.94 | 3.93 | 3.93 | 3.93 | 4.04 | 3.97 | 3.96 | 3.88 |
| **9.7** | **1.48** | 3.61 | 3.73 | 3.74 | 3.75 | 3.77 | 3.82 | 3.82 | 3.75 | 3.81 | 3.76 | 3.9 | 3.8 |
| **9.7** | **1.14** | 3.89 | 3.87 | 4.04 | 3.99 | 3.81 | 3.85 | 3.97 | 3.99 | 3.79 | 3.85 | 3.89 | 4.06 |
| **6.21** | **6.05** | 2.97 | 2.96 | 2.96 | 2.97 | 2.96 | 2.96 | 2.96 | 2.96 | 2.96 | 2.96 | 2.96 | 2.95 |
| **6.21** | **5.57** | 2.53 | 2.53 | 2.53 | 2.53 | 2.53 | 2.53 | 2.53 | 2.54 | 2.54 | 2.53 | 2.53 | 2.54 |
| **6.21** | **4.96** |  |  |  |  |  |  |  |  |  |  | **4.92** |  |
| **6.21** | **4.5** | 3.67 | 3.68 | 3.7 | 3.68 | 3.74 | 3.69 | 3.75 | 3.74 | 3.68 | 3.75 | 3.79 | 3.72 |
| **6.21** | **4.42** | 4.21 | 4.3 | 4.3 | 4.32 | 4.21 | 4.3 | 4.48 | 4.25 | 4.4 | 4.44 | 4.51 | 4.98 |
| **6.21** | **4.02** | 2.53 | 2.54 | 2.54 | 2.54 | 2.53 | 2.53 | 2.54 | 2.54 | 2.53 | 2.54 | 2.53 | 2.54 |
| **6.21** | **3.91** |  | **4.54** | **4.21** |  |  |  |  |  | **4.17** | **4.22** |  |  |
| **6.21** | **3.84** | 3.85 | 3.89 | 3.9 | 3.76 | 3.85 | 3.95 | 3.89 | 4.12 | 3.94 | 3.98 | 3.84 |  |
| **6.21** | **3.65** | 2.84 | 2.84 | 2.85 | 2.85 | 2.85 | 2.87 | 2.86 | 2.88 | 2.86 | 2.87 | 2.86 | 2.87 |
| **6.21** | **3.03** | 3.85 | 4.05 | 4.07 | 4.08 | 3.95 | 4.17 | 4.29 | 4.08 | 4.12 | 4.44 | 4.25 | 4.1 |
| **6.21** | **2.67** | 4.17 | 4.18 | 3.94 | 3.88 | 4.47 |  | 4.21 |  | 4.36 | 4.3 | 3.94 |  |
| **6.21** | **2.28** |  |  | **4.31** | **4.84** |  |  | **4.37** |  | **4.55** |  |  |  |
| **6.21** | **2.22** | 3.74 | 3.79 | 3.78 | 3.75 | 3.63 | 3.83 | 3.76 | 3.75 | 3.8 | 3.82 | 3.81 | 3.97 |
| **6.21** | **2.17** | 2.38 | 2.37 | 2.4 | 2.4 | 2.38 | 2.42 | 2.4 | 2.39 | 2.42 | 2.42 | 2.47 | 2.51 |
| **6.21** | **2.09** | 3.73 |  | 3.79 | 3.7 |  | 3.38 |  | 3.59 |  |  |  |  |
| **6.21** | **1.91** | 3.47 | 3.47 | 3.42 | 3.57 | 3.52 | 3.55 | 3.57 | 3.56 | 3.66 | 3.55 | 3.52 |  |
| **6.21** | **1.48** | 4.15 | 3.94 | 4.04 |  | 4.04 |  |  | 4.18 |  |  | 3.89 | 4.74 |
| **6.21** | **1.3** |  |  |  |  |  |  | **5.46** |  |  |  |  |  |
| **6.21** | **0.97** | 3.38 | 3.4 | 3.44 | 3.52 | 3.42 | 3.51 | 3.51 | 3.46 | 3.59 | 3.64 | 3.61 | 3.72 |
| **6.21** | **0.86** | 4.05 | 4.11 | 4.14 | 4.35 | 4.47 | 3.97 |  | 3.86 | 4.29 | 4.4 | 4.28 | 4.22 |
| **6.05** | **5.57** | 2.49 | 2.49 | 2.49 | 2.5 | 2.5 | 2.5 | 2.5 | 2.5 | 2.49 | 2.5 | 2.5 | 2.49 |
| **6.05** | **4.96** | 4.02 | 3.99 | 4.05 | 4.08 | 4.12 | 4.05 |  | 4.09 | 4.17 | 4 |  | 4.06 |
| **6.05** | **4.5** |  |  |  |  |  |  | **5.07** |  |  |  |  |  |
| **6.05** | **4.02** | 3.91 | 4 | 3.97 | 4.02 | 3.88 | 3.95 | 4.03 | 3.99 | 4.07 | 4.17 | 4.07 | 4.09 |
| **6.05** | **3.65** | 3.43 | 3.47 | 3.43 | 3.46 | 3.49 | 3.55 | 3.48 | 3.53 | 3.52 | 3.51 | 3.6 | 3.55 |
| **6.05** | **3.03** |  | **4.32** |  | **4.45** |  | **4.73** | **4.31** | **4.51** | **4.66** | **4.42** |  | **4.46** |
| **6.05** | **2.28** | 3.98 | 4.01 | 4.06 | 4.11 | 4 | 4.08 | 4.12 | 4.13 | 4.18 | 3.95 | 4.03 | 4.02 |
| **6.05** | **2.17** |  |  |  |  |  |  |  | **3.53** |  |  |  |  |
| **6.05** | **2.09** | 2.3 | 2.31 | 2.32 | 2.3 | 2.31 | 2.32 | 2.31 | 2.33 | 2.35 | 2.34 | 2.36 | 2.36 |
| **6.05** | **1.23** | 4.55 | 4.34 | 4.34 | 4.42 | 4.48 | 4.44 | 4.54 | 4.3 | 4.33 | 4.37 |  | 4.39 |
| **6.05** | **0.97** | 3.52 | 3.58 | 3.58 | 3.66 | 3.77 | 3.71 | 3.67 | 3.76 | 3.58 | 3.75 | 4.03 | 3.57 |
| **6.05** | **0.86** | 4.03 | 4.03 | 4.08 | 4.04 | 4.03 | 4.03 | 3.88 | 4.12 | 4.07 | 4.23 | 4.17 | 3.93 |
| **5.57** | **4.96** | 2.99 | 3 | 3 | 2.99 | 2.98 | 3 | 3 | 3.02 | 3.01 | 3.01 | 2.99 | 3.02 |
| **5.57** | **4.5** | 3.86 | 3.9 | 3.92 | 3.84 | 3.89 | 3.92 | 3.9 | 3.87 | 3.87 | 3.96 | 4 | 4.08 |
| **5.57** | **4.42** |  |  |  |  |  |  |  | **4.68** |  |  |  |  |
| **5.57** | **4.02** | 3.31 | 3.33 | 3.33 | 3.34 | 3.3 | 3.34 | 3.33 | 3.4 | 3.38 | 3.4 | 3.32 | 3.37 |
| **5.57** | **3.91** |  |  |  |  |  |  |  |  |  | **4.64** |  |  |
| **5.57** | **3.65** | 3.25 | 3.27 | 3.26 | 3.27 | 3.25 | 3.3 | 3.3 | 3.33 | 3.31 | 3.32 | 3.35 | 3.33 |
| **5.57** | **3.03** | 3.76 | 3.89 | 3.88 | 3.83 | 3.72 | 3.88 | 3.97 | 3.92 | 4.13 | 4.11 | 3.88 | 4.34 |
| **5.57** | **2.28** | 4.41 | 4.41 | 4.62 | 4.38 | 4.29 | 4.48 | 4.47 | 4.58 | 4.6 | 4.27 | 4.44 | 4.49 |
| **5.57** | **2.22** |  |  |  |  | **4.26** |  |  |  |  |  |  |  |
| **5.57** | **2.17** | 3.54 | 3.26 | 3.28 |  | 3.41 |  |  |  |  |  | 3.42 |  |
| **5.57** | **2.09** | 2.71 | 2.85 | 2.87 | 2.8 | 2.77 | 2.93 | 2.72 | 2.89 | 2.92 | 2.84 | 2.94 | 2.95 |
| **5.57** | **1.91** | 3.61 | 3.61 | 3.66 | 3.61 | 3.65 | 3.71 | 3.8 | 3.76 | 3.78 | 3.75 | 3.65 | 3.91 |
| **5.57** | **1.48** | 3.22 | 3.23 | 3.26 | 3.28 | 3.26 | 3.31 | 3.33 | 3.36 | 3.31 | 3.37 | 3.34 | 3.4 |
| **5.57** | **1.23** | 4.18 | 4.26 | 4.17 | 4.26 | 4.36 | 4.38 | 4.42 |  |  | 4.42 | 4.37 | 4.35 |
| **5.57** | **0.97** | 2.77 | 2.77 | 2.78 | 2.79 | 2.78 | 2.81 | 2.82 | 2.81 | 2.8 | 2.85 | 2.82 | 2.85 |
| **5.57** | **0.86** | 2.87 | 2.88 | 2.87 | 2.88 | 2.87 | 2.87 | 2.88 | 2.87 | 2.87 | 2.89 | 2.89 | 2.9 |
| **4.96** | **4.02** |  | **4.33** | **4.42** | **4.27** |  | **4.5** | **4.26** |  |  |  |  |  |
| **4.96** | **3.91** | 4.14 | 3.9 | 3.86 | 4.11 | 3.9 | 3.94 | 3.89 | 3.97 | 4.02 | 3.85 | 3.8 | 3.92 |
| **4.96** | **3.65** |  |  |  |  |  |  | **4.36** |  |  |  |  | **4.56** |
| **4.96** | **3.35** | 4.38 |  |  | 4.4 |  |  |  |  |  |  | 4.35 |  |
| **4.96** | **3.14** | 3.59 | 3.61 | 3.63 | 3.66 | 3.61 | 3.65 | 3.64 | 3.66 | 3.67 | 3.59 | 3.71 | 3.66 |
| **4.96** | **3.08** | 2.72 | 2.72 | 2.72 | 2.72 | 2.73 | 2.71 | 2.73 | 2.73 | 2.72 | 2.73 | 2.71 | 2.73 |
| **4.96** | **2.77** | 4.05 | 4.15 | 4.02 | 3.96 | 4.21 | 4.16 | 4.07 | 4.18 | 4.04 | 4.39 |  | 4.19 |
| **4.96** | **2.35** | 3.76 | 3.87 | 3.73 |  | 3.76 | 3.93 | 3.89 |  | 3.8 | 3.87 | 3.99 | 3.68 |
| **4.96** | **2.28** |  | **4.18** |  |  |  |  | **4.59** |  | **4.97** |  | **4.67** |  |
| **4.96** | **2.09** | 2.76 | 2.78 | 2.79 | 2.83 | 2.8 | 2.81 | 2.83 | 2.81 | 2.81 | 2.83 | 2.9 | 2.79 |
| **4.96** | **1.81** | 2.84 | 2.84 | 2.85 | 2.85 | 2.85 | 2.86 | 2.87 | 2.86 | 2.85 | 2.85 | 2.85 | 2.87 |
| **4.96** | **1.66** | 2.48 | 2.49 | 2.48 | 2.49 | 2.48 | 2.49 | 2.49 | 2.49 | 2.49 | 2.49 | 2.49 | 2.49 |
| **4.96** | **1.23** | 2.97 | 2.98 | 2.98 | 2.98 | 2.97 | 2.98 | 2.99 | 2.99 | 2.99 | 2.99 | 2.98 | 2.99 |
| **4.96** | **1.14** | 3.01 | 3 | 3.02 | 3.02 | 3.02 | 2.99 | 3.01 | 3 | 3.01 | 3.01 | 3.01 | 3.02 |
| **4.96** | **1.05** |  |  |  |  |  |  |  | **4.3** |  | **4.43** |  | **4.3** |
| **4.9** | **4.5** | 3.53 | 3.56 | 3.57 | 3.56 | 3.56 | 3.55 | 3.59 | 3.57 | 3.51 | 3.52 | 3.57 | 3.58 |
| **4.9** | **4.38** | 3.38 | 3.39 | 3.4 | 3.4 | 3.4 | 3.46 | 3.4 | 3.4 | 3.41 | 3.39 | 3.41 | 3.42 |
| **4.9** | **4.08** | 3.28 | 3.31 | 3.24 | 3.34 | 3.3 | 3.33 | 3.27 | 3.34 | 3.24 | 3.3 | 3.34 | 3.25 |
| **4.9** | **4.02** |  |  |  |  |  |  |  | **5.37** |  |  |  |  |
| **4.9** | **3.91** | 2.38 | 2.39 | 2.39 | 2.38 | 2.38 | 2.38 | 2.39 | 2.39 | 2.39 | 2.38 | 2.39 | 2.39 |
| **4.9** | **3.84** | 3.91 |  |  | 3.93 | 3.97 |  | 3.92 | 4.06 |  | 4.22 | 3.95 | 3.81 |
| **4.9** | **3.39** | 3.17 | 3.22 | 3.19 | 3.23 | 3.21 | 3.21 | 3.21 | 3.26 | 3.21 | 3.21 | 3.21 | 3.21 |
| **4.9** | **3.35** | 2.53 | 2.54 | 2.53 | 2.55 | 2.55 | 2.54 | 2.58 | 2.56 | 2.53 | 2.55 | 2.55 | 2.54 |
| **4.9** | **3.14** |  |  |  |  |  |  |  |  |  | **4.1** |  |  |
| **4.9** | **3.08** | 3.8 | 3.87 | 3.84 | 3.83 | 3.64 | 3.83 | 3.89 | 3.89 | 3.8 | 3.9 | 3.82 | 3.59 |
| **4.9** | **3.03** | 3.96 | 4.02 |  | 4.02 |  |  | 3.89 | 3.98 |  | 4.07 | 4.02 | 4.35 |
| **4.9** | **2.42** | 3.25 | 3.25 | 3.27 | 3.38 | 3.24 | 3.33 | 3.3 | 3.29 | 3.25 | 3.33 | 3.18 | 3.24 |
| **4.9** | **2.35** | 3.21 | 3.17 | 3.17 | 3.18 | 3.1 | 3.16 | 3.06 | 3.08 | 3.25 | 3.25 | 3.13 | 3.08 |
| **4.9** | **1.81** | 4.76 |  |  |  |  |  |  |  |  |  |  |  |
| **4.9** | **1.66** |  |  |  |  |  |  |  |  |  | **5.37** | **4.75** |  |
| **4.9** | **1.48** |  |  | **4.21** | **4.26** |  |  |  | **4.01** | **4.19** |  | **4.35** |  |
| **4.9** | **1.23** |  |  |  |  |  |  |  |  | **4.68** | **5.26** |  |  |
| **4.9** | **1.14** |  |  | **5.12** |  |  |  |  |  |  |  |  |  |
| **4.5** | **4.38** | 4.46 |  |  | 4.09 | 4.23 | 4.65 |  | 4.17 | 4.13 | 4.18 | 4.11 |  |
| **4.5** | **4.08** | 3.27 | 3.29 | 3.32 | 3.27 | 3.33 | 3.31 | 3.3 | 3.31 | 3.36 | 3.23 | 3.24 | 3.31 |
| **4.5** | **3.91** | 2.82 | 2.83 | 2.84 | 2.84 | 2.85 | 2.86 | 2.86 | 2.84 | 2.85 | 2.84 | 2.86 | 2.86 |
| **4.5** | **3.84** | 3.03 | 3.04 | 3.05 | 3.05 | 3.03 | 3.06 | 3.06 | 3.03 | 3.04 | 3.04 | 3.04 | 3.08 |
| **4.5** | **3.65** | 2.66 | 2.67 | 2.67 | 2.67 | 2.66 | 2.68 | 2.68 | 2.68 | 2.68 | 2.68 | 2.69 | 2.69 |
| **4.5** | **3.39** |  |  |  |  |  |  |  |  |  | **4.28** |  |  |
| **4.5** | **3.03** | 3.65 | 3.75 | 3.72 | 3.69 | 3.48 | 3.5 | 3.61 | 3.68 | 3.61 | 3.65 |  | 3.58 |
| **4.5** | **2.42** |  |  |  |  |  |  |  |  |  |  | **3.79** |  |
| **4.5** | **2.28** | 3.39 | 3.43 | 3.42 | 3.42 | 3.43 | 3.46 | 3.44 | 3.44 | 3.44 | 3.45 | 3.49 | 3.42 |
| **4.5** | **2.17** | 2.8 |  |  | 2.82 |  | 2.94 | 2.83 |  | 2.93 | 2.86 |  |  |
| **4.42** | **4.02** | 3.34 | 3.39 | 3.36 | 3.4 | 3.36 | 3.34 | 3.38 | 3.37 | 3.31 | 3.39 | 3.37 | 3.36 |
| **4.42** | **3.91** | 4.06 | 4 | 4.27 |  | 4.22 |  | 4.16 | 4.2 | 3.97 | 3.95 |  | 4.01 |
| **4.38** | **3.91** | 4.26 |  |  | 4.19 |  | 3.99 | 4.06 |  | 4.39 |  |  | 4.47 |
| **4.38** | **3.84** | 2.28 | 2.28 | 2.28 | 2.28 | 2.28 | 2.28 | 2.29 | 2.28 | 2.28 | 2.28 | 2.28 | 2.28 |
| **4.38** | **3.65** | 4.05 |  |  |  |  |  | 4.16 | 4.13 |  | 4.03 |  |  |
| **4.42** | **3.43** | 3.63 | 3.68 | 3.66 | 3.63 | 3.74 | 3.73 | 3.66 | 3.68 | 3.72 | 3.72 | 3.76 | 3.69 |
| **4.38** | **3.39** | 2.99 | 3.02 | 3.06 | 3.06 | 3.01 | 3.07 | 3.08 | 3.07 | 3.03 | 3.07 | 3.02 | 3.04 |
| **4.38** | **3.35** | 3.32 | 3.36 | 3.36 | 3.31 | 3.31 |  |  | 3.42 | 3.36 | 3.33 |  | 3.54 |
| **4.38** | **3.14** | 2.25 | 2.25 | 2.25 | 2.25 | 2.25 | 2.25 | 2.26 | 2.25 | 2.25 | 2.25 | 2.25 | 2.25 |
| **4.38** | **3.08** | 3.16 | 3.16 | 3.16 | 3.18 | 3.18 | 3.16 | 3.17 | 3.17 | 3.14 | 3.16 | 3.17 | 3.16 |
| **4.38** | **3.03** | 3.33 | 3.33 | 3.3 | 3.33 | 3.31 | 3.31 | 3.33 | 3.37 | 3.32 | 3.37 | 3.32 | 3.36 |
| **4.42** | **2.67** | 3.71 | 3.79 |  |  |  |  |  |  | 3.9 | 3.89 |  |  |
| **4.38** | **2.67** | 3.78 | 3.81 | 3.89 | 3.83 | 3.94 | 4 | 3.85 | 4.04 | 3.77 | 3.76 | 3.98 | 3.86 |
| **4.38** | **2.42** |  | **3.79** | **3.95** | **3.72** | **3.85** |  |  |  |  |  | **4.05** |  |
| **4.38** | **2.35** | 2.41 | 2.41 | 2.41 | 2.42 | 2.41 | 2.41 | 2.41 | 2.42 | 2.41 | 2.42 | 2.4 | 2.42 |
| **4.42** | **2.22** | 3.38 | 3.47 | 3.4 | 3.46 | 3.42 | 3.52 | 3.56 | 3.46 | 3.43 | 3.56 | 3.55 | 3.63 |
| **4.38** | **2.22** | 3.69 | 3.84 | 3.62 | 3.67 | 3.74 |  | 3.84 |  | 3.91 | 3.67 | 3.81 |  |
| **4.38** | **2.17** |  |  | **3.46** | **3.26** |  |  |  | **3.28** |  |  |  |  |
| **4.42** | **1.91** | 2.59 | 2.6 | 2.59 | 2.6 | 2.59 | 2.6 | 2.6 | 2.6 | 2.6 | 2.6 | 2.59 | 2.61 |
| **4.38** | **1.91** |  |  |  |  |  |  |  |  | **4.69** |  |  |  |
| **4.42** | **1.74** | 3.42 | 3.5 | 3.48 | 3.46 | 3.52 | 3.58 | 3.53 | 3.57 | 3.51 | 3.52 | 3.51 | 3.51 |
| **4.38** | **1.48** | 4.52 | 4.22 |  | 4.14 |  |  |  |  | 4.38 | 4 |  |  |
| **4.42** | **1.4** | 2.73 | 2.74 | 2.73 | 2.73 | 2.73 | 2.73 | 2.74 | 2.74 | 2.74 | 2.75 | 2.73 | 2.76 |
| **4.42** | **1.3** |  | **3.67** |  |  |  |  |  |  |  |  |  |  |
| **4.38** | **1.14** | 4.08 | 4.19 | 4.19 |  | 4.28 | 4.13 | 4.23 | 4.3 |  | 4.5 | 4.29 | 4.09 |
| **4.42** | **0.86** | 3.6 | 3.68 | 3.67 | 3.69 | 3.57 | 3.64 | 3.64 | 3.73 | 3.61 | 3.69 | 3.64 | 3.81 |
| **4.08** | **3.91** | 2.28 | 2.28 | 2.28 | 2.28 | 2.28 | 2.28 | 2.29 | 2.29 | 2.28 | 2.29 | 2.29 | 2.29 |
| **4.08** | **2.42** |  |  |  |  |  |  |  |  |  | **3.81** |  |  |
| **4.08** | **1.91** | 4.65 | 4.8 | 4.86 | 4.23 | 4.44 |  |  | 4.93 |  | 4.61 | 4.53 |  |
| **4.08** | **1.14** | 3.69 | 3.97 | 3.86 | 3.71 | 3.73 | 3.79 | 3.78 | 3.68 | 3.8 | 3.78 | 3.53 | 3.76 |
| **4.08** | **1.05** |  |  |  |  |  |  |  |  | **5.18** |  |  |  |
| **4.02** | **3.91** | 2.89 | 2.89 | 2.89 | 2.89 | 2.88 | 2.89 | 2.88 | 2.87 | 2.86 | 2.88 | 2.87 | 2.91 |
| **4.02** | **3.84** | 3.86 | 3.8 | 3.77 | 3.79 | 3.83 | 3.91 | 3.76 | 3.79 | 3.85 | 3.83 | 3.75 | 4 |
| **4.02** | **3.65** | 3.6 | 3.61 | 3.62 | 3.69 | 3.63 | 3.79 | 3.63 | 3.67 | 3.72 | 3.74 | 3.6 | 3.63 |
| **4.02** | **3.08** | 3.16 | 3.16 | 3.17 | 3.17 | 3.2 | 3.19 | 3.15 | 3.19 | 3.21 | 3.2 | 3.2 | 3.19 |
| **4.02** | **2.77** | 3.11 | 3.11 | 3.12 | 3.12 | 3.15 | 3.14 | 3.13 | 3.12 | 3.13 | 3.13 | 3.13 | 3.14 |
| **4.02** | **2.67** | 2.92 | 2.93 | 2.93 | 2.93 | 2.94 | 2.93 | 2.95 | 2.97 | 2.97 | 2.95 | 2.95 | 2.96 |
| **4.02** | **2.42** | 3.54 | 3.55 | 3.55 | 3.43 | 3.51 | 3.45 | 3.54 | 3.51 | 3.54 | 3.5 | 3.5 | 3.44 |
| **4.02** | **2.35** |  |  |  | **3.85** |  |  | **3.79** |  |  |  |  |  |
| **4.02** | **2.22** | 2.53 | 2.54 | 2.53 | 2.54 | 2.53 | 2.54 | 2.54 | 2.54 | 2.54 | 2.55 | 2.53 | 2.55 |
| **4.02** | **2.17** | 2.41 | 2.42 | 2.42 | 2.43 | 2.4 | 2.43 | 2.43 | 2.42 | 2.45 | 2.45 | 2.43 | 2.43 |
| **4.02** | **1.91** | 2.61 | 2.62 | 2.61 | 2.61 | 2.62 | 2.62 | 2.62 | 2.62 | 2.62 | 2.63 | 2.61 | 2.63 |
| **4.02** | **1.66** |  |  |  |  |  |  |  |  |  | **4.28** |  |  |
| **4.02** | **1.48** | 3.37 | 3.41 | 3.37 | 3.41 | 3.4 | 3.46 | 3.42 | 3.5 | 3.4 | 3.42 |  | 3.44 |
| **4.02** | **1.14** | 3.36 | 3.35 | 3.36 | 3.39 | 3.37 | 3.37 | 3.35 | 3.37 | 3.42 | 3.37 | 3.39 | 3.4 |
| **4.02** | **1.05** |  |  |  |  |  |  | **4.46** |  |  |  |  |  |
| **4.02** | **0.97** | 3.28 | 3.31 | 3.29 | 3.34 | 3.24 | 3.31 | 3.33 | 3.37 | 3.3 | 3.41 | 3.34 | 3.31 |
| **4.02** | **0.86** | 3.75 | 3.73 | 3.79 | 3.76 | 3.78 | 3.78 | 3.79 | 3.85 | 3.86 | 3.8 | 3.8 | 3.86 |
| **3.91** | **3.35** |  |  | **3.83** | **3.98** |  |  |  |  |  |  |  |  |
| **3.91** | **3.14** | 3.91 |  | 3.87 | 3.91 | 3.85 | 3.85 |  | 3.86 |  | 3.92 | 4.23 | 3.73 |
| **3.91** | **3.08** | 3.89 | 3.98 | 3.91 | 3.87 | 3.94 | 3.91 | 3.9 | 3.88 | 3.87 | 3.78 | 3.83 | 3.71 |
| **3.91** | **2.77** | 3.84 | 3.89 | 3.91 | 3.74 | 3.82 | 3.84 | 3.86 | 3.89 | 3.88 | 3.87 | 3.84 | 3.81 |
| **3.91** | **2.42** |  | **3.72** |  |  |  |  | **3.63** |  |  | **3.53** |  |  |
| **3.91** | **2.35** | 3.18 | 3.24 | 3.2 | 3.21 | 3.17 | 3.15 | 3.17 | 3.25 |  | 3.24 | 3.27 |  |
| **3.91** | **1.81** | 3.27 | 3.31 | 3.33 | 3.3 | 3.29 | 3.32 | 3.42 | 3.36 | 3.26 | 3.42 | 3.33 | 3.4 |
| **3.91** | **1.66** | 3.75 | 3.87 | 4.06 | 3.97 | 3.76 | 3.74 |  |  | 3.98 |  | 4.16 |  |
| **3.91** | **1.14** |  | **3.98** |  | **4.25** |  |  |  |  | **4.12** |  | **4.57** |  |
| **3.91** | **1.05** | 3.65 | 3.83 | 3.93 |  | 3.55 | 3.56 | 3.69 | 3.62 | 3.7 |  | 3.47 |  |
| **3.84** | **3.65** | 2.9 | 2.91 | 2.9 | 2.9 | 2.9 | 2.92 | 2.9 | 2.91 | 2.92 | 2.9 | 2.92 | 2.91 |
| **3.84** | **3.39** | 3.51 | 3.55 | 3.55 | 3.77 | 3.53 | 3.6 | 3.55 |  | 3.68 |  | 3.6 | 3.58 |
| **3.84** | **3.14** | 3.1 | 3.13 | 3.14 | 3.14 | 3.13 | 3.16 | 3.15 | 3.15 | 3.13 | 3.14 | 3.15 | 3.16 |
| **3.84** | **3.08** | 4.22 |  |  |  |  |  |  |  | 4.24 |  |  |  |
| **3.84** | **3.03** | 2.91 | 2.94 | 2.94 | 2.92 | 2.92 | 2.92 | 2.93 | 2.92 | 2.94 | 2.96 | 2.93 | 2.93 |
| **3.84** | **2.67** | 2.79 | 2.8 | 2.81 | 2.8 | 2.81 | 2.84 | 2.81 | 2.83 | 2.82 | 2.82 | 2.81 | 2.82 |
| **3.84** | **2.35** |  |  |  |  |  |  |  | **3.52** |  |  | **3.67** |  |
| **3.84** | **2.28** | 3.73 | 3.83 | 3.74 | 3.73 | 3.74 | 4.06 | 3.72 | 4.04 |  | 3.92 | 4.13 |  |
| **3.84** | **2.22** | 2.78 | 2.8 | 2.8 | 2.79 | 2.8 | 2.8 | 2.81 | 2.8 | 2.81 | 2.8 | 2.81 | 2.81 |
| **3.84** | **2.17** | 2.21 | 2.21 | 2.22 | 2.22 | 2.21 | 2.26 | 2.21 | 2.24 | 2.24 | 2.23 | 2.23 | 2.26 |
| **3.84** | **1.91** | 3.49 | 3.54 | 3.51 | 3.5 | 3.53 | 3.52 | 3.54 | 3.61 | 3.57 | 3.67 | 3.6 | 3.7 |
| **3.84** | **1.48** | 3.41 | 3.43 | 3.5 | 3.46 | 3.43 | 3.64 | 3.36 | 3.44 | 3.34 | 3.52 | 3.47 | 3.61 |
| **3.84** | **0.97** | 3.11 | 3.16 | 3.19 | 3.19 | 3.17 | 3.55 | 3.2 | 3.19 | 3.38 | 3.2 | 3.38 | 3.46 |
| **3.65** | **3.39** | 3.4 | 3.35 | 3.41 | 3.27 | 3.29 | 3.33 | 3.45 | 3.37 |  | 3.44 | 3.38 |  |
| **3.65** | **3.03** | 2.35 | 2.36 | 2.35 | 2.35 | 2.35 | 2.35 | 2.36 | 2.36 | 2.36 | 2.36 | 2.36 | 2.35 |
| **3.65** | **2.67** | 3.7 |  | 3.8 |  | 3.88 |  |  |  |  |  |  |  |
| **3.65** | **2.42** | 3.23 | 3.31 | 3.29 | 3.46 | 3.27 | 3.25 | 3.32 | 3.35 | 3.28 | 3.52 | 3.27 | 3.29 |
| **3.65** | **2.28** | 2.55 | 2.55 | 2.55 | 2.55 | 2.55 | 2.55 | 2.55 | 2.56 | 2.55 | 2.56 | 2.56 | 2.56 |
| **3.65** | **2.22** | 3.54 | 3.52 | 3.52 | 3.54 | 3.54 | 3.52 | 3.61 | 3.54 | 3.66 | 3.58 | 3.5 | 3.53 |
| **3.65** | **2.17** | 2.13 | 2.13 | 2.14 | 2.14 | 2.13 | 2.14 | 2.15 | 2.13 | 2.15 | 2.14 | 2.16 | 2.16 |
| **3.65** | **1.91** | 3.66 | 3.69 | 3.61 | 3.79 | 3.82 |  |  | 3.67 |  | 3.84 |  |  |
| **3.65** | **1.48** | 2.97 | 2.97 | 2.98 | 2.95 | 2.94 | 3.01 | 2.99 | 3.02 | 3.03 | 3.05 | 3.05 | 3.01 |
| **3.65** | **0.97** | 2.6 | 2.6 | 2.61 | 2.62 | 2.6 | 2.64 | 2.64 | 2.63 | 2.63 | 2.63 | 2.67 | 2.63 |
| **3.65** | **0.86** |  | **4.31** |  |  | **4.67** |  | **4.53** |  |  |  | **4.52** |  |
| **3.39** | **3.14** |  | **4.15** | **4.35** |  |  |  | **4.27** | **4.18** | **4.48** | **4.21** |  |  |
| **3.39** | **3.08** |  |  |  |  |  |  | **4.11** | **4.17** |  |  | **4.2** |  |
| **3.39** | **3.03** | 2.48 | 2.48 | 2.48 | 2.48 | 2.48 | 2.47 | 2.48 | 2.48 | 2.48 | 2.48 | 2.48 | 2.47 |
| **3.39** | **2.42** | 3.27 | 3.28 | 3.31 | 3.27 | 3.32 | 3.29 | 3.3 | 3.31 | 3.29 | 3.27 | 3.34 | 3.28 |
| **3.39** | **2.35** | 4.01 |  | 3.77 |  | 3.75 | 3.93 | 4.06 | 3.9 | 4.12 | 4.27 | 4.08 |  |
| **3.39** | **2.28** | 2.7 | 2.7 | 2.7 | 2.69 | 2.69 | 2.69 | 2.7 | 2.71 | 2.7 | 2.72 | 2.69 | 2.72 |
| **3.43** | **2.13** | 3.54 | 3.57 | 3.55 | 3.57 | 3.55 | 3.58 | 3.63 | 3.57 | 3.56 | 3.56 | 3.67 | 3.57 |
| **3.43** | **2.03** |  |  | **4.7** |  |  |  |  |  |  |  |  |  |
| **3.39** | **1.48** |  |  | **3.93** | **3.95** |  |  |  |  |  |  |  |  |
| **3.43** | **1.4** | 3.69 | 3.59 | 3.69 | 3.7 | 3.75 | 3.72 |  | 3.69 |  |  | 3.82 | 3.76 |
| **3.39** | **0.97** | 3.65 | 3.66 | 3.65 | 3.66 | 3.77 | 3.73 | 3.67 | 3.74 | 3.71 | 3.8 |  | 4.02 |
| **3.35** | **3.14** | 3.57 | 3.42 | 3.54 | 3.62 | 3.48 | 3.62 | 3.73 | 3.74 |  | 3.47 | 3.36 |  |
| **3.35** | **3.08** | 2.65 | 2.66 | 2.64 | 2.67 | 2.65 | 2.65 | 2.64 | 2.64 | 2.65 | 2.67 | 2.66 | 2.69 |
| **3.35** | **2.67** |  |  |  |  |  | **3.66** | **3.89** |  | **4.05** |  | **3.79** |  |
| **3.35** | **2.42** | 2.73 | 2.72 | 2.72 | 2.73 | 2.73 | 2.73 | 2.74 | 2.76 | 2.73 | 2.74 | 2.72 | 2.75 |
| **3.35** | **2.35** | 2.99 | 2.97 | 2.96 | 3 | 3.05 | 3.04 | 3.06 | 3.03 | 3 | 3.02 | 3.19 | 3.05 |
| **3.35** | **2.28** |  |  |  |  |  |  |  |  |  |  |  | **3.81** |
| **3.35** | **2.22** |  |  |  |  |  |  |  | **3.82** |  |  |  |  |
| **3.14** | **3.08** | 2.59 | 2.59 | 2.59 | 2.59 | 2.6 | 2.59 | 2.6 | 2.59 | 2.59 | 2.59 | 2.59 | 2.59 |
| **3.14** | **2.67** |  |  |  |  |  |  |  |  |  |  |  | **4.29** |
| **3.08** | **2.67** | 4.08 | 4.18 |  | 4 | 4.44 |  | 4.16 |  | 4.28 | 4.21 |  | 4.18 |
| **3.14** | **2.35** | 2.47 | 2.47 | 2.47 | 2.48 | 2.46 | 2.47 | 2.47 | 2.48 | 2.48 | 2.5 | 2.45 | 2.48 |
| **3.08** | **2.42** | 3.12 | 3.1 | 3.14 | 3.11 | 3.1 | 3.1 | 3.15 | 3.1 | 3.15 | 3.2 | 3.1 | 3.09 |
| **3.14** | **1.81** | 4.03 | 4.27 | 4.13 | 4.51 | 4.32 | 4.06 |  | 4.12 | 4.4 | 4.31 | 4.25 | 4.37 |
| **3.08** | **1.81** | 4.12 | 4.16 |  | 4.38 | 4.39 | 4.3 | 4.12 | 4.14 |  | 4.63 | 4.22 | 4.48 |
| **3.08** | **1.66** | 3.76 | 3.78 | 3.73 | 3.89 | 3.9 | 3.83 | 3.84 | 3.82 | 4.11 |  | 3.85 | 3.86 |
| **3.14** | **1.14** | 3.1 | 3.11 | 3.12 | 3.12 | 3.1 | 3.12 | 3.13 | 3.16 | 3.12 | 3.15 | 3.14 | 3.17 |
| **3.08** | **1.14** | 3.15 | 3.18 | 3.17 | 3.16 | 3.18 | 3.14 | 3.17 | 3.16 | 3.17 | 3.2 | 3.16 | 3.2 |
| **3.08** | **1.05** |  |  |  |  |  |  |  |  |  |  |  | **4.86** |
| **3.03** | **2.67** | 4.06 |  |  | 3.94 |  |  |  |  |  |  |  |  |
| **3.03** | **2.42** | 3.39 | 3.51 | 3.37 | 3.45 | 3.4 | 3.42 | 3.38 |  |  | 3.41 | 3.34 | 3.31 |
| **3.03** | **2.28** | 2.51 | 2.51 | 2.51 | 2.51 | 2.5 | 2.5 | 2.5 | 2.52 | 2.51 | 2.5 | 2.52 | 2.51 |
| **3.03** | **2.22** | 3.62 |  | 3.49 | 3.61 | 3.51 | 3.52 | 3.97 |  |  |  |  | 3.58 |
| **3.03** | **2.17** | 2.79 | 2.68 | 2.85 | 2.86 | 2.84 | 2.81 | 2.83 | 2.72 | 2.87 | 3.11 | 2.93 | 2.79 |
| **3.03** | **1.91** | 3.77 |  |  |  |  |  |  |  |  |  |  | 3.96 |
| **3.03** | **1.48** | 2.54 | 2.55 | 2.55 | 2.55 | 2.55 | 2.55 | 2.56 | 2.56 | 2.56 | 2.56 | 2.57 | 2.56 |
| **3.03** | **0.97** | 2.25 | 2.25 | 2.25 | 2.25 | 2.24 | 2.24 | 2.26 | 2.25 | 2.25 | 2.26 | 2.26 | 2.24 |
| **3.03** | **0.86** | 3.85 | 3.78 | 3.85 | 3.84 | 3.79 | 3.73 |  | 3.87 | 4.01 | 4 |  | 3.94 |
| **2.77** | **2.28** | 3.91 |  | 4.14 | 3.89 |  |  | 4.37 | 3.83 | 4.35 | 4.79 |  | 4.59 |
| **2.77** | **2.22** |  |  |  | **4.16** |  |  | **3.95** |  | **4.4** |  | **4.05** |  |
| **2.77** | **1.81** | 3.48 | 3.52 | 3.5 | 3.5 | 3.51 | 3.53 | 3.57 | 3.53 | 3.51 | 3.54 | 3.51 | 3.45 |
| **2.77** | **1.66** | 2.85 | 2.85 | 2.86 | 2.86 | 2.86 | 2.87 | 2.87 | 2.86 | 2.87 | 2.87 | 2.87 | 2.89 |
| **2.77** | **1.14** | 3.34 | 3.36 | 3.36 | 3.39 | 3.4 | 3.36 | 3.36 | 3.41 | 3.34 | 3.35 | 3.42 | 3.38 |
| **2.77** | **1.05** | 3.54 | 3.57 | 3.54 | 3.54 | 3.58 | 3.57 | 3.59 | 3.51 | 3.55 | 3.64 | 3.64 | 3.53 |
| **2.67** | **2.22** | 1.86 | 1.86 | 1.86 | 1.86 | 1.86 | 1.86 | 1.86 | 1.86 | 1.86 | 1.86 | 1.86 | 1.86 |
| **2.67** | **2.17** | 2.39 | 2.46 | 2.4 | 2.44 | 2.43 | 2.46 | 2.52 | 2.42 | 2.41 | 2.44 | 2.4 | 2.41 |
| **2.67** | **1.91** | 2.58 | 2.59 | 2.59 | 2.58 | 2.6 | 2.6 | 2.6 | 2.61 | 2.6 | 2.6 | 2.6 | 2.62 |
| **2.67** | **1.48** | 2.77 | 2.78 | 2.8 | 2.79 | 2.83 | 2.85 | 2.86 | 2.82 | 2.83 | 2.8 | 2.85 | 2.83 |
| **2.67** | **0.97** | 3.05 | 3.09 | 3.08 | 3.11 | 3.19 | 3.29 | 3.18 |  | 3.14 | 3.19 |  | 3.17 |
| **2.67** | **0.86** |  |  |  |  |  |  |  |  |  |  | **4.04** |  |
| **2.42** | **2.35** | 2.8 | 2.9 | 2.9 | 2.79 | 2.92 | 2.93 | 2.72 | 2.7 | 2.94 | 2.89 | 2.94 | 2.82 |
| **2.42** | **2.28** | 2.2 | 2.2 | 2.2 | 2.2 | 2.2 | 2.2 | 2.21 | 2.21 | 2.2 | 2.21 | 2.19 | 2.2 |
| **2.42** | **1.14** | 3.82 | 3.88 |  | 3.81 | 3.85 | 3.83 | 3.79 | 3.91 | 3.76 | 4.07 | 3.97 | 3.84 |
| **2.35** | **1.14** |  |  |  |  |  |  |  |  | **4.99** |  |  |  |
| **2.28** | **1.91** | 4.17 |  |  | 4.04 |  | 4.27 | 4.46 |  | 4.25 |  |  |  |
| **2.28** | **1.23** |  |  |  |  |  |  |  |  |  |  |  | **4.47** |
| **2.28** | **0.97** | 3.35 | 3.4 | 3.33 | 3.4 | 3.34 | 3.4 | 3.44 | 3.53 | 3.24 | 3.32 |  | 3.46 |
| **2.22** | **2.17** | 2.28 | 2.3 | 2.29 | 2.29 | 2.29 | 2.28 | 2.29 | 2.26 | 2.29 | 2.29 | 2.31 | 2.31 |
| **2.22** | **2.13** | 3.41 |  |  |  | 3.53 |  |  |  |  | 3.95 | 3.36 |  |
| **2.22** | **2.03** | 3.67 | 3.87 | 3.96 |  | 3.53 |  |  | 3.9 |  | 4.03 |  | 3.87 |
| **2.22** | **1.91** | 2.25 | 2.26 | 2.26 | 2.26 | 2.26 | 2.27 | 2.27 | 2.26 | 2.26 | 2.26 | 2.26 | 2.28 |
| **2.17** | **1.91** | 2.79 | 2.78 | 2.82 | 2.82 | 2.82 | 2.81 | 2.84 | 2.79 | 2.82 | 2.81 | 2.82 | 2.97 |
| **2.22** | **1.48** | 2.81 | 2.81 | 2.82 | 2.85 | 2.9 | 2.9 | 2.88 | 2.81 | 2.89 | 2.86 | 2.89 | 2.82 |
| **2.17** | **1.48** | 2.72 | 2.73 | 2.77 | 2.82 | 2.76 | 2.91 | 2.89 | 2.74 | 2.71 | 2.75 | 2.78 |  |
| **2.22** | **0.97** | 3.11 | 3.2 | 3.1 | 3.13 | 3.14 | 3.15 | 3.15 | 3.13 | 3.12 | 3.05 | 3.07 | 3.15 |
| **2.17** | **0.97** | 2.46 | 2.47 | 2.46 | 2.45 | 2.51 | 2.57 | 2.52 | 2.52 | 2.51 | 2.51 | 2.51 | 2.5 |
| **2.22** | **0.86** | 3.59 | 3.6 | 3.7 | 3.6 | 3.85 | 3.81 | 3.73 | 3.76 | 3.62 |  |  | 3.56 |
| **2.17** | **0.86** |  |  |  |  |  |  |  |  |  |  | **3.44** |  |
| **2.13** | **2.03** | 3.35 | 3.32 | 3.31 | 3.3 | 3.29 | 3.31 | 3.3 | 3.3 | 3.29 | 3.31 | 3.33 | 3.32 |
| **2.13** | **1.48** | 4.82 | 4.31 | 4.57 | 4.39 | 4.49 | 4.4 | 4.82 |  | 4.32 | 4.39 | 4.56 | 4.34 |
| **2.13** | **1.23** |  | **4.71** |  |  | **5.33** |  | **5.09** | **5.36** |  |  |  |  |
| **2.09** | **1.23** | 2.9 | 2.94 | 2.92 | 2.89 | 2.89 | 2.9 | 2.97 | 2.96 | 2.9 | 2.95 | 2.92 | 2.94 |
| **2.13** | **0.97** | 4.38 | 4.08 | 3.95 | 4.24 | 4.3 | 4.03 |  | 4.5 | 4.21 |  |  | 4.39 |
| **2.03** | **1.74** | 3.98 | 3.8 | 3.9 | 3.86 | 3.98 | 4.1 | 3.78 | 3.96 | 4.05 | 3.97 | 4.02 | 3.89 |
| **2.03** | **1.3** | 3.89 | 3.86 | 3.91 | 3.84 | 3.82 | 4.12 | 3.96 | 3.85 |  | 3.91 | 4.18 | 3.66 |
| **1.91** | **1.48** | 2.39 | 2.39 | 2.39 | 2.39 | 2.4 | 2.4 | 2.41 | 2.4 | 2.4 | 2.41 | 2.41 | 2.42 |
| **1.91** | **1.4** | 4.15 | 4.3 | 4.13 | 3.92 | 4.12 |  | 4.16 |  | 4.49 |  | 4.09 | 4.28 |
| **1.91** | **0.97** | 2.62 | 2.62 | 2.65 | 2.63 | 2.65 | 2.68 | 2.68 | 2.67 | 2.65 | 2.67 | 2.7 | 2.7 |
| **1.91** | **0.86** | 2.71 | 2.71 | 2.71 | 2.71 | 2.72 | 2.71 | 2.73 | 2.73 | 2.72 | 2.73 | 2.73 | 2.73 |
| **1.81** | **1.66** | 2 | 2 | 2 | 2 | 2 | 2 | 2 | 2 | 2 | 2 | 2 | 2 |
| **1.81** | **1.14** | 3.56 | 3.67 |  | 3.56 | 3.66 | 3.71 | 3.74 | 3.53 | 3.58 | 3.85 | 3.58 | 3.66 |
| **1.81** | **1.05** | 2.9 | 2.89 | 2.91 | 2.9 | 2.91 | 2.89 | 2.92 | 2.9 | 2.92 | 2.89 | 2.91 | 2.89 |
| **1.74** | **1.4** | 2.65 | 2.65 | 2.65 | 2.65 | 2.65 | 2.66 | 2.65 | 2.67 | 2.66 | 2.66 | 2.66 | 2.66 |
| **1.66** | **1.4** |  |  |  |  |  |  |  |  | **3.61** |  |  |  |
| **1.74** | **1.3** | 2.78 | 2.9 | 2.89 | 2.88 | 2.9 | 2.91 | 2.86 | 2.87 | 2.87 | 2.79 | 2.91 | 2.79 |
| **1.66** | **1.14** |  | **3.74** |  |  |  |  |  |  |  |  |  |  |
| **1.66** | **1.05** | 3.09 | 3.08 | 3.1 | 3.12 | 3.1 | 3.1 | 3.12 | 3.08 | 3.08 | 3.15 | 3.12 | 3.15 |
| **1.74** | **0.86** |  |  | **4.48** | **4.42** |  |  |  |  |  |  |  |  |
| **1.48** | **0.97** | 1.78 | 1.78 | 1.78 | 1.78 | 1.78 | 1.78 | 1.78 | 1.78 | 1.78 | 1.78 | 1.78 | 1.78 |
| **1.48** | **0.86** | 2.66 | 2.66 | 2.65 | 2.67 | 2.65 | 2.65 | 2.67 | 2.66 | 2.66 | 2.67 | 2.66 | 2.68 |
| **1.4** | **1.3** | 2.6 | 2.61 | 2.62 | 2.59 | 2.6 | 2.61 | 2.62 | 2.6 | 2.63 | 2.62 | 2.62 | 2.61 |
| **1.3** | **1.23** | 3.55 | 3.44 | 3.43 | 3.52 | 3.52 | 3.78 | 3.44 | 3.38 | 3.52 | 3.5 | 3.48 | 3.49 |
| **1.09** | **1.05** | 3.23 | 3.19 | 3.2 | 3.18 | 3.23 | 3.2 | 3.19 | 3.22 | 3.17 | 3.14 |  | 3.17 |
| **1.05** | **0.97** |  |  |  |  |  |  |  | **3.81** |  |  |  | **4.02** |
| **0.97** | **0.86** | 2.76 | 2.77 | 2.77 | 2.79 | 2.78 | 2.77 | 2.78 | 2.78 | 2.79 | 2.75 | 2.77 | 2.78 |

Table S9: Interproton distances calculated from SAAR-best and SAAR-worst sampling schemes as compared to the uniformly sampled reference spectra. Red distances signify a greater than 7% deviation from the uniformly sampled data. Blue distances signify an interproton distance that was not valid in the uniformly sampled data but is valid in the NUS dataset (R^2^ > 0.90, n > 4). Assignments are given as chemical shifts (ppm).

| f2 δ  (ppm) | f1 δ  (ppm) | Ref.  r (Å) | SAAR-best  r (Å) | SAAR-worst  r (Å) |
| --- | --- | --- | --- | --- |
| **9.7** | **6.21** | 4.56 | 4.44 | 4.56 |
| **9.7** | **4.5** | 4.21 | 4.45 | 4.23 |
| **9.7** | **4.38** | 4.21 | 4.36 |  |
| **9.7** | **4.42** |  |  | **5.53** |
| **9.7** | **4.02** | 4.16 | 4.37 | 4.11 |
| **9.7** | **3.84** | 3.01 | 3.02 | 3.03 |
| **9.7** | **3.65** | 4.38 | 4.64 | 4.59 |
| **9.7** | **3.35** | 3.86 |  |  |
| **9.7** | **3.14** |  |  | **4.86** |
| **9.7** | **3.08** |  |  | **4.87** |
| **9.7** | **2.67** | 3.23 | 3.28 | 3.29 |
| **9.7** | **2.22** | 3.19 | 3.27 | 3.21 |
| **9.7** | **2.17** | 3 | 3.18 | 3.1 |
| **9.7** | **1.91** | 3.75 | 3.92 | 3.87 |
| **9.7** | **1.48** | 3.61 | 3.87 | 3.67 |
| **9.7** | **1.14** | 3.89 | 4.06 | 3.89 |
| **6.21** | **6.05** | 2.97 | 2.97 | 2.99 |
| **6.21** | **5.57** | 2.53 | 2.53 | 2.54 |
| **6.21** | **4.5** | 3.67 | 3.72 | 3.67 |
| **6.21** | **4.42** | 4.21 | 4.63 | 4.28 |
| **6.21** | **4.02** | 2.53 | 2.54 | 2.54 |
| **6.21** | **3.91** |  | **4.31** | **4.46** |
| **6.21** | **3.84** | 3.85 | 3.93 | 3.92 |
| **6.21** | **3.65** | 2.84 | 2.87 | 2.86 |
| **6.21** | **3.03** | 3.85 | 4.16 | 4.23 |
| **6.21** | **2.67** | 4.17 | 3.96 | 4.03 |
| **6.21** | **2.28** |  | **4.65** |  |
| **6.21** | **2.22** | 3.74 | 3.77 | 3.81 |
| **6.21** | **2.17** | 2.38 | 2.41 | 2.42 |
| **6.21** | **2.09** | 3.73 | 3.77 |  |
| **6.21** | **1.91** | 3.47 | 3.56 | 3.56 |
| **6.21** | **1.48** | 4.15 | 4.03 | 4.26 |
| **6.21** | **0.97** | 3.38 | 3.5 | 3.57 |
| **6.21** | **0.86** | 4.05 | 4.67 |  |
| **6.05** | **5.57** | 2.49 | 2.5 | 2.51 |
| **6.05** | **4.96** | 4.02 | 4.06 |  |
| **6.05** | **4.02** | 3.91 | 4.23 | 3.88 |
| **6.05** | **3.65** | 3.43 | 3.49 | 3.5 |
| **6.05** | **3.03** |  | **4.45** | **4.43** |
| **6.05** | **2.28** | 3.98 | 3.98 | 4.23 |
| **6.05** | **2.17** |  | **3.6** |  |
| **6.05** | **2.09** | 2.3 | 2.3 | 2.36 |
| **6.05** | **1.48** |  | **4.35** |  |
| **6.05** | **1.23** | 4.55 | 4.27 |  |
| **6.05** | **0.97** | 3.52 | 3.54 | 3.73 |
| **6.05** | **0.86** | 4.03 | 4 |  |
| **5.57** | **4.96** | 2.99 | 2.98 | 3.02 |
| **5.57** | **4.5** | 3.86 | 3.93 | 3.86 |
| **5.57** | **4.02** | 3.31 | 3.32 | 3.36 |
| **5.57** | **3.84** |  |  | **4.41** |
| **5.57** | **3.65** | 3.25 | 3.29 | 3.26 |
| **5.57** | **3.03** | 3.76 | 3.79 | 3.89 |
| **5.57** | **2.28** | 4.41 | 4.43 |  |
| **5.57** | **2.17** | 3.54 |  | 3.23 |
| **5.57** | **2.09** | 2.71 | 2.91 | 2.87 |
| **5.57** | **1.91** | 3.61 | 3.71 | 3.76 |
| **5.57** | **1.48** | 3.22 | 3.34 | 3.35 |
| **5.57** | **1.23** | 4.18 |  |  |
| **5.57** | **0.97** | 2.77 | 2.79 | 2.81 |
| **5.57** | **0.86** | 2.87 | 2.88 | 2.89 |
| **4.96** | **4.02** |  | **4.31** |  |
| **4.96** | **3.91** | 4.14 | 3.97 | 3.59 |
| **4.96** | **3.35** | 4.38 |  | 4.16 |
| **4.96** | **3.14** | 3.59 | 3.62 | 3.6 |
| **4.96** | **3.08** | 2.72 | 2.72 | 2.72 |
| **4.96** | **2.77** | 4.05 |  | 3.94 |
| **4.96** | **2.35** | 3.76 | 3.97 | 4 |
| **4.96** | **2.09** | 2.76 | 2.85 | 2.82 |
| **4.96** | **1.81** | 2.84 | 2.86 | 2.86 |
| **4.96** | **1.66** | 2.48 | 2.49 | 2.48 |
| **4.96** | **1.23** | 2.97 | 2.98 | 3 |
| **4.96** | **1.14** | 3.01 | 3.01 | 3.03 |
| **4.9** | **4.5** | 3.53 | 3.59 | 3.55 |
| **4.9** | **4.38** | 3.38 | 3.4 | 3.45 |
| **4.9** | **4.08** | 3.28 | 3.29 | 3.3 |
| **4.9** | **3.91** | 2.38 | 2.39 | 2.39 |
| **4.9** | **3.84** | 3.91 |  | 4.23 |
| **4.9** | **3.39** | 3.17 | 3.18 | 3.25 |
| **4.9** | **3.35** | 2.53 | 2.55 | 2.55 |
| **4.9** | **3.14** |  | **4.22** |  |
| **4.9** | **3.08** | 3.8 | 3.63 | 3.94 |
| **4.9** | **3.03** | 3.96 | 3.95 | 4.13 |
| **4.9** | **2.42** | 3.25 | 3.19 | 3.26 |
| **4.9** | **2.35** | 3.21 | 3.19 | 3.2 |
| **4.9** | **1.81** | 4.76 |  |  |
| **4.9** | **1.48** |  | **4.3** | **4.16** |
| **4.5** | **4.38** | 4.46 | 4.87 | 4.39 |
| **4.5** | **4.08** | 3.27 | 3.27 | 3.31 |
| **4.5** | **3.91** | 2.82 | 2.84 | 2.85 |
| **4.5** | **3.84** | 3.03 | 3.04 | 3.06 |
| **4.5** | **3.65** | 2.66 | 2.67 | 2.67 |
| **4.5** | **3.03** | 3.65 | 3.75 | 3.71 |
| **4.5** | **2.28** | 3.39 | 3.44 | 3.44 |
| **4.5** | **2.17** | 2.8 | 2.82 | 2.86 |
| **4.42** | **4.02** | 3.34 | 3.37 | 3.37 |
| **4.42** | **3.91** | 4.06 | 4.21 |  |
| **4.38** | **3.91** | 4.26 |  | 4.14 |
| **4.38** | **3.84** | 2.28 | 2.28 | 2.28 |
| **4.38** | **3.65** | 4.05 |  |  |
| **4.42** | **3.43** | 3.63 | 3.7 | 3.71 |
| **4.38** | **3.39** | 2.99 | 3.08 | 3.01 |
| **4.38** | **3.35** | 3.32 | 3.37 | 3.32 |
| **4.38** | **3.14** | 2.25 | 2.25 | 2.25 |
| **4.38** | **3.08** | 3.16 | 3.17 | 3.18 |
| **4.38** | **3.03** | 3.33 | 3.33 | 3.3 |
| **4.42** | **2.67** | 3.71 | 3.9 |  |
| **4.38** | **2.67** | 3.78 | 3.91 | 3.92 |
| **4.38** | **2.42** |  |  | **3.81** |
| **4.38** | **2.35** | 2.41 | 2.42 | 2.41 |
| **4.42** | **2.22** | 3.38 | 3.47 | 3.42 |
| **4.38** | **2.22** | 3.69 | 3.65 | 3.62 |
| **4.38** | **2.17** |  | **3.08** | **3.29** |
| **4.42** | **1.91** | 2.59 | 2.61 | 2.6 |
| **4.42** | **1.74** | 3.42 | 3.5 | 3.49 |
| **4.38** | **1.48** | 4.52 | 4.27 | 4.09 |
| **4.42** | **1.4** | 2.73 | 2.74 | 2.75 |
| **4.38** | **1.14** | 4.08 | 4.28 |  |
| **4.42** | **0.86** | 3.6 | 3.7 | 3.68 |
| **4.08** | **3.91** | 2.28 | 2.29 | 2.29 |
| **4.08** | **2.42** |  |  | **4.06** |
| **4.08** | **1.91** | 4.65 | 4.51 | 4.12 |
| **4.08** | **1.14** | 3.69 | 3.84 | 3.68 |
| **4.08** | **0.97** |  |  | **3.77** |
| **4.02** | **3.91** | 2.89 | 2.88 | 2.88 |
| **4.02** | **3.84** | 3.86 | 3.76 | 3.8 |
| **4.02** | **3.65** | 3.6 | 3.6 | 3.64 |
| **4.02** | **3.08** | 3.16 | 3.17 | 3.19 |
| **4.02** | **2.77** | 3.11 | 3.13 | 3.14 |
| **4.02** | **2.67** | 2.92 | 2.94 | 2.96 |
| **4.02** | **2.42** | 3.54 | 3.53 | 3.53 |
| **4.02** | **2.35** |  | **4.3** | **3.91** |
| **4.02** | **2.22** | 2.53 | 2.54 | 2.53 |
| **4.02** | **2.17** | 2.41 | 2.44 | 2.45 |
| **4.02** | **1.91** | 2.61 | 2.62 | 2.62 |
| **4.02** | **1.48** | 3.37 | 3.39 | 3.49 |
| **4.02** | **1.14** | 3.36 | 3.38 | 3.42 |
| **4.02** | **0.97** | 3.28 | 3.32 | 3.32 |
| **4.02** | **0.86** | 3.75 | 3.76 | 3.81 |
| **3.91** | **3.14** | 3.91 | 3.91 | 3.85 |
| **3.91** | **3.08** | 3.89 | 3.78 | 4.11 |
| **3.91** | **2.77** | 3.84 | 3.8 | 3.85 |
| **3.91** | **2.42** |  |  | **3.52** |
| **3.91** | **2.35** | 3.18 | 3.24 | 3.11 |
| **3.91** | **1.81** | 3.27 | 3.32 | 3.26 |
| **3.91** | **1.66** | 3.75 | 3.84 | 3.93 |
| **3.91** | **1.14** |  | **4.49** |  |
| **3.91** | **1.05** | 3.65 | 3.67 | 3.6 |
| **3.84** | **3.65** | 2.9 | 2.91 | 2.9 |
| **3.84** | **3.39** | 3.51 | 3.53 |  |
| **3.84** | **3.14** | 3.1 | 3.16 | 3.16 |
| **3.84** | **3.08** | 4.22 |  | 4.13 |
| **3.84** | **3.03** | 2.91 | 2.94 | 2.93 |
| **3.84** | **2.67** | 2.79 | 2.8 | 2.82 |
| **3.84** | **2.28** | 3.73 | 3.88 | 3.89 |
| **3.84** | **2.22** | 2.78 | 2.8 | 2.8 |
| **3.84** | **2.17** | 2.21 | 2.24 | 2.23 |
| **3.84** | **1.91** | 3.49 | 3.51 | 3.61 |
| **3.84** | **1.48** | 3.41 | 3.34 | 3.3 |
| **3.84** | **0.97** | 3.11 | 3.18 | 3.22 |
| **3.65** | **3.39** | 3.4 |  | 3.41 |
| **3.65** | **3.03** | 2.35 | 2.36 | 2.35 |
| **3.65** | **2.67** | 3.7 | 3.68 |  |
| **3.65** | **2.42** | 3.23 | 3.26 | 3.27 |
| **3.65** | **2.28** | 2.55 | 2.55 | 2.56 |
| **3.65** | **2.22** | 3.54 | 3.5 | 3.49 |
| **3.65** | **2.17** | 2.13 | 2.14 | 2.14 |
| **3.65** | **1.91** | 3.66 | 3.54 | 3.77 |
| **3.65** | **1.48** | 2.97 | 2.94 | 2.96 |
| **3.65** | **0.97** | 2.6 | 2.63 | 2.62 |
| **3.39** | **3.14** |  | **4.23** | **4.32** |
| **3.39** | **3.03** | 2.48 | 2.48 | 2.47 |
| **3.39** | **2.42** | 3.27 | 3.33 | 3.3 |
| **3.39** | **2.35** | 4.01 |  |  |
| **3.39** | **2.28** | 2.7 | 2.71 | 2.7 |
| **3.43** | **2.13** | 3.54 | 3.46 | 3.6 |
| **3.39** | **1.48** |  |  | **3.8** |
| **3.43** | **1.4** | 3.69 | 3.66 | 3.66 |
| **3.39** | **0.97** | 3.65 | 3.73 | 3.56 |
| **3.35** | **3.14** | 3.57 | 3.58 |  |
| **3.35** | **3.08** | 2.65 | 2.65 | 2.64 |
| **3.35** | **2.42** | 2.73 | 2.72 | 2.74 |
| **3.35** | **2.35** | 2.99 | 2.9 | 3 |
| **3.14** | **3.08** | 2.59 | 2.6 | 2.6 |
| **3.08** | **2.67** | 4.08 |  | 3.96 |
| **3.14** | **2.35** | 2.47 | 2.49 | 2.49 |
| **3.08** | **2.42** | 3.12 | 3.15 | 3.16 |
| **3.14** | **1.81** | 4.03 |  | 4.11 |
| **3.08** | **1.81** | 4.12 | 4.19 | 4.13 |
| **3.08** | **1.66** | 3.76 | 3.78 | 3.74 |
| **3.14** | **1.14** | 3.1 | 3.13 | 3.11 |
| **3.08** | **1.14** | 3.15 | 3.11 | 3.2 |
| **3.03** | **2.67** | 4.06 |  |  |
| **3.03** | **2.42** | 3.39 | 3.42 | 3.55 |
| **3.03** | **2.28** | 2.51 | 2.51 | 2.51 |
| **3.03** | **2.22** | 3.62 |  |  |
| **3.03** | **2.17** | 2.79 | 2.88 | 2.92 |
| **3.03** | **1.91** | 3.77 |  |  |
| **3.03** | **1.48** | 2.54 | 2.56 | 2.55 |
| **3.03** | **0.97** | 2.25 | 2.26 | 2.25 |
| **3.03** | **0.86** | 3.85 |  | 3.84 |
| **2.77** | **2.28** | 3.91 |  | 4.06 |
| **2.77** | **2.22** |  | **4.1** | **4.33** |
| **2.77** | **1.81** | 3.48 | 3.54 | 3.44 |
| **2.77** | **1.66** | 2.85 | 2.87 | 2.86 |
| **2.77** | **1.14** | 3.34 | 3.37 | 3.32 |
| **2.77** | **1.05** | 3.54 | 3.59 | 3.56 |
| **2.67** | **2.22** | 1.86 | 1.86 | 1.86 |
| **2.67** | **2.17** | 2.39 | 2.4 | 2.39 |
| **2.67** | **1.91** | 2.58 | 2.59 | 2.59 |
| **2.67** | **1.48** | 2.77 | 2.79 | 2.79 |
| **2.67** | **1.14** |  |  | **3.9** |
| **2.67** | **0.97** | 3.05 | 3.11 | 3.1 |
| **2.42** | **2.35** | 2.8 | 2.83 | 2.98 |
| **2.42** | **2.28** | 2.2 | 2.2 | 2.21 |
| **2.42** | **1.14** | 3.82 | 3.91 | 3.89 |
| **2.28** | **1.91** | 4.17 |  |  |
| **2.28** | **1.48** |  |  | **3.58** |
| **2.28** | **0.97** | 3.35 | 3.31 | 3.27 |
| **2.22** | **2.17** | 2.28 | 2.28 | 2.3 |
| **2.22** | **2.13** | 3.41 |  |  |
| **2.22** | **2.03** | 3.67 | 3.8 | 3.87 |
| **2.22** | **1.91** | 2.25 | 2.26 | 2.26 |
| **2.17** | **1.91** | 2.79 | 2.78 | 2.77 |
| **2.22** | **1.48** | 2.81 | 2.89 | 2.88 |
| **2.17** | **1.48** | 2.72 |  | 2.84 |
| **2.22** | **0.97** | 3.11 | 3.13 | 3.24 |
| **2.17** | **0.97** | 2.46 | 2.47 | 2.48 |
| **2.22** | **0.86** | 3.59 | 3.66 |  |
| **2.13** | **2.03** | 3.35 | 3.32 | 3.33 |
| **2.13** | **1.48** | 4.82 | 4.87 | 4.39 |
| **2.09** | **1.23** | 2.9 | 2.92 | 2.93 |
| **2.13** | **0.97** | 4.38 | 4.23 |  |
| **2.03** | **1.74** | 3.98 | 3.77 | 3.93 |
| **2.03** | **1.3** | 3.89 |  |  |
| **1.91** | **1.48** | 2.39 | 2.4 | 2.4 |
| **1.91** | **1.4** | 4.15 |  | 4.23 |
| **1.91** | **0.97** | 2.62 | 2.65 | 2.64 |
| **1.91** | **0.86** | 2.71 | 2.72 | 2.73 |
| **1.81** | **1.66** | 2 | 2 | 2 |
| **1.81** | **1.14** | 3.56 |  | 3.6 |
| **1.81** | **1.05** | 2.9 | 2.92 | 2.91 |
| **1.74** | **1.4** | 2.65 | 2.66 | 2.65 |
| **1.66** | **1.4** |  |  | **4.04** |
| **1.74** | **1.3** | 2.78 | 2.81 | 2.91 |
| **1.66** | **1.05** | 3.09 | 3.11 | 3.09 |
| **1.48** | **0.97** | 1.78 | 1.78 | 1.78 |
| **1.48** | **0.86** | 2.66 | 2.68 | 2.66 |
| **1.4** | **1.3** | 2.6 | 2.61 | 2.63 |
| **1.3** | **1.23** | 3.55 | 3.53 | 3.54 |
| **1.09** | **1.05** | 3.23 | 3.17 | 3.19 |
| **0.97** | **0.86** | 2.76 | 2.78 | 2.79 |

Table S10: Interproton distances calculated from US-NUS hybrid schemes 512-50-25 to 512-50-35 sampling schemes as compared to the uniformly sampled reference spectra. Red distances signify a greater than 7% deviation from the uniformly sampled data. Blue distances signify an interproton distance that was not valid in the uniformly sampled data but were valid in the NUS dataset (R^2^ > 0.90, n > 4). Assignments are given as chemical shifts (ppm).

| f2 δ  (ppm) | f1 δ  (ppm) | Ref.  r (Å) | 512-50-25-0  r (Å) | 512-50-25-1  r (Å) | 512-50-25-2  r (Å) | 512-50-30-0  r (Å) | 512-50-30-1  r (Å) | 512-50-30-2  r (Å) | 512-50-35-0  r (Å) | 512-50-35-1  r (Å) | 512-50-35-2  r (Å) |
| --- | --- | --- | --- | --- | --- | --- | --- | --- | --- | --- | --- |
| **9.7** | **6.21** | 4.56 | 4.6 | 4.64 | 4.63 | 4.62 | 4.63 | 4.63 | 4.58 | 4.62 | 4.58 |
| **9.7** | **4.5** | 4.21 | 4.26 | 4.23 | 4.33 | 4.17 | 4.3 | 4.29 | 4.21 | 4.23 | 4.24 |
| **9.7** | **4.38** | 4.21 | 4.21 | 4.32 | 4.31 | 4.25 | 4.26 | 4.2 | 4.23 | 4.24 | 4.22 |
| **9.7** | **4.42** |  |  | **5.64** |  |  |  |  |  | **5.63** |  |
| **9.7** | **4.02** | 4.16 | 4.17 | 4.34 | 4.31 | 4.27 | 4.26 | 4.26 | 4.05 | 4.19 | 4.2 |
| **9.7** | **3.84** | 3.01 | 3.01 | 3.02 | 3.01 | 3.01 | 3.01 | 3.01 | 3.02 | 3.01 | 3.01 |
| **9.7** | **3.65** | 4.38 | 4.4 | 4.65 | 4.59 | 4.5 | 4.53 | 4.51 | 4.37 | 4.41 | 4.39 |
| **9.7** | **3.35** | 3.86 | 3.93 | 3.94 | 3.8 | 3.93 | 4.04 | 4.01 | 3.92 | 3.92 | 3.98 |
| **9.7** | **3.14** |  | **4.59** |  |  | **4.62** |  |  |  |  |  |
| **9.7** | **3.08** |  |  | **4.77** | **4.7** |  |  |  |  |  |  |
| **9.7** | **2.67** | 3.23 | 3.24 | 3.26 | 3.27 | 3.24 | 3.25 | 3.25 | 3.24 | 3.24 | 3.25 |
| **9.7** | **2.22** | 3.19 | 3.21 | 3.24 | 3.23 | 3.22 | 3.22 | 3.21 | 3.21 | 3.21 | 3.22 |
| **9.7** | **2.17** | 3 |  |  |  |  |  |  | 3.04 | 3.05 | 3.02 |
| **9.7** | **1.91** | 3.75 | 3.81 | 3.83 | 3.82 | 3.8 | 3.82 | 3.83 | 3.77 | 3.82 | 3.84 |
| **9.7** | **1.48** | 3.61 | 3.66 | 3.69 | 3.67 | 3.64 | 3.65 | 3.67 | 3.62 | 3.65 | 3.65 |
| **9.7** | **1.14** | 3.89 | 3.96 | 3.95 | 3.94 | 3.94 | 3.93 | 3.93 | 3.9 | 3.95 | 3.93 |
| **6.21** | **6.05** | 2.97 | 2.97 | 2.97 | 2.97 | 2.97 | 2.97 | 2.96 | 2.97 | 2.97 | 2.97 |
| **6.21** | **5.57** | 2.53 | 2.53 | 2.53 | 2.53 | 2.53 | 2.53 | 2.53 | 2.53 | 2.53 | 2.53 |
| **6.21** | **4.5** | 3.67 | 3.66 | 3.66 | 3.66 | 3.67 | 3.67 | 3.65 | 3.67 | 3.67 | 3.66 |
| **6.21** | **4.42** | 4.21 | 4.18 | 4.25 | 4.24 | 4.2 | 4.18 | 4.18 | 4.24 | 4.25 | 4.18 |
| **6.21** | **4.02** | 2.53 | 2.53 | 2.53 | 2.53 | 2.54 | 2.53 | 2.53 | 2.53 | 2.53 | 2.53 |
| **6.21** | **3.91** |  |  |  |  | **4.56** |  | **4.33** |  |  | **4.82** |
| **6.21** | **3.84** | 3.85 | 3.84 | 3.84 | 3.83 | 3.84 | 3.84 | 3.83 | 3.85 | 3.84 | 3.85 |
| **6.21** | **3.65** | 2.84 | 2.84 | 2.84 | 2.85 | 2.84 | 2.84 | 2.84 | 2.84 | 2.84 | 2.84 |
| **6.21** | **3.03** | 3.85 | 4.02 | 3.98 | 4 | 3.97 | 3.94 | 3.92 | 3.93 | 3.94 | 4 |
| **6.21** | **2.67** | 4.17 | 3.91 | 3.89 | 3.88 | 3.89 | 3.9 | 3.86 | 3.91 | 3.94 | 4.14 |
| **6.21** | **2.22** | 3.74 | 3.75 | 3.73 | 3.73 | 3.75 | 3.76 | 3.76 | 3.75 | 3.75 | 3.75 |
| **6.21** | **2.17** | 2.38 | 2.38 | 2.38 | 2.38 | 2.38 | 2.38 | 2.39 | 2.39 | 2.39 | 2.38 |
| **6.21** | **2.09** | 3.73 | 3.65 | 3.66 | 3.66 | 3.74 | 3.72 | 3.71 | 3.71 | 3.71 | 3.66 |
| **6.21** | **1.91** | 3.47 | 3.45 | 3.46 | 3.46 | 3.47 | 3.47 | 3.46 | 3.47 | 3.47 | 3.47 |
| **6.21** | **1.48** | 4.15 | 4 | 3.94 | 4.01 | 4.01 | 3.99 | 3.95 | 3.99 | 4.02 | 4.01 |
| **6.21** | **0.97** | 3.38 | 3.36 | 3.37 | 3.37 | 3.38 | 3.38 | 3.36 | 3.37 | 3.37 | 3.37 |
| **6.21** | **0.86** | 4.05 | 4.05 | 4.01 | 4.04 | 4.03 | 4.05 |  |  |  | 4.1 |
| **6.05** | **5.57** | 2.49 | 2.49 | 2.49 | 2.49 | 2.49 | 2.49 | 2.49 | 2.5 | 2.49 | 2.49 |
| **6.05** | **4.96** | 4.02 | 4.02 | 4.03 | 4 | 4.01 | 4.02 | 4.01 | 4.01 | 4.03 | 4.01 |
| **6.05** | **4.02** | 3.91 | 3.9 | 3.92 | 3.96 | 3.91 | 3.91 | 3.86 | 3.9 | 3.92 | 3.9 |
| **6.05** | **3.65** | 3.43 | 3.42 | 3.44 | 3.44 | 3.43 | 3.43 | 3.42 | 3.44 | 3.44 | 3.43 |
| **6.05** | **2.28** | 3.98 | 3.98 | 4.02 | 4.03 | 4 | 3.98 | 3.98 | 4.01 | 4.01 | 4 |
| **6.05** | **2.09** | 2.3 | 2.3 | 2.3 | 2.31 | 2.3 | 2.3 | 2.3 | 2.31 | 2.3 | 2.3 |
| **6.05** | **1.91** |  | **5.3** | **5.01** | **5.04** | **5.25** | **5.32** |  | **5.01** | **5.17** |  |
| **6.05** | **1.23** | 4.55 | 4.32 | 4.52 | 4.35 | 4.57 | 4.52 | 4.51 | 4.54 | 4.55 | 4.55 |
| **6.05** | **0.97** | 3.52 | 3.51 | 3.53 | 3.51 | 3.53 | 3.52 | 3.51 | 3.57 | 3.59 | 3.53 |
| **6.05** | **0.86** | 4.03 | 4.01 | 4.04 | 4.05 | 4.03 | 4 | 4.02 | 4 | 4.05 | 4 |
| **5.57** | **4.96** | 2.99 | 2.99 | 2.99 | 2.99 | 2.99 | 2.99 | 2.99 | 2.99 | 2.99 | 2.99 |
| **5.57** | **4.5** | 3.86 | 3.83 | 3.86 | 3.85 | 3.85 | 3.85 | 3.85 | 3.86 | 3.86 | 3.84 |
| **5.57** | **4.42** |  | **4.48** |  |  |  |  |  |  |  |  |
| **5.57** | **4.02** | 3.31 | 3.3 | 3.3 | 3.3 | 3.3 | 3.31 | 3.3 | 3.3 | 3.3 | 3.3 |
| **5.57** | **3.65** | 3.25 | 3.24 | 3.25 | 3.25 | 3.25 | 3.24 | 3.25 | 3.25 | 3.24 | 3.24 |
| **5.57** | **3.03** | 3.76 | 3.74 | 3.81 | 3.81 | 3.76 | 3.76 | 3.8 | 3.76 | 3.77 | 3.79 |
| **5.57** | **2.28** | 4.41 | 4.35 | 4.35 | 4.38 | 4.34 | 4.36 | 4.34 | 4.37 | 4.38 | 4.35 |
| **5.57** | **2.17** | 3.54 | 3.43 | 3.46 | 3.47 | 3.42 | 3.45 | 3.45 | 3.39 | 3.44 | 3.46 |
| **5.57** | **2.09** | 2.71 | 2.82 | 2.73 | 2.81 | 2.74 | 2.81 | 2.81 | 2.81 | 2.81 | 2.8 |
| **5.57** | **1.91** | 3.61 | 3.59 | 3.62 | 3.61 | 3.6 | 3.6 | 3.62 | 3.61 | 3.63 | 3.6 |
| **5.57** | **1.48** | 3.22 | 3.22 | 3.22 | 3.22 | 3.22 | 3.22 | 3.23 | 3.22 | 3.24 | 3.23 |
| **5.57** | **1.23** | 4.18 | 4.21 | 4.18 | 4.21 | 4.21 | 4.2 | 4.22 | 4.22 | 4.25 | 4.24 |
| **5.57** | **0.97** | 2.77 | 2.77 | 2.78 | 2.77 | 2.77 | 2.77 | 2.77 | 2.77 | 2.77 | 2.77 |
| **5.57** | **0.86** | 2.87 | 2.87 | 2.87 | 2.87 | 2.87 | 2.87 | 2.87 | 2.87 | 2.87 | 2.87 |
| **4.96** | **4.5** |  |  |  |  |  | **4.64** | **4.6** |  | **4.78** | **4.68** |
| **4.96** | **4.02** |  | **4.31** | **4.3** | **4.31** |  | **4.3** |  | **4.27** | **4.34** | **4.38** |
| **4.96** | **3.91** | 4.14 | 3.78 | 3.82 | 4.16 | 3.8 | 3.77 | 4.15 | 4.09 | 4.16 | 3.81 |
| **4.96** | **3.65** |  |  |  |  |  |  |  |  |  |  |
| **4.96** | **3.35** | 4.38 |  |  |  |  |  |  |  |  |  |
| **4.96** | **3.14** | 3.59 | 3.6 | 3.61 | 3.6 | 3.59 | 3.58 | 3.58 | 3.6 | 3.59 | 3.61 |
| **4.96** | **3.08** | 2.72 | 2.72 | 2.72 | 2.73 | 2.72 | 2.72 | 2.72 | 2.72 | 2.72 | 2.73 |
| **4.96** | **2.77** | 4.05 | 3.96 | 4.09 | 3.96 | 4.08 | 4.07 | 4.06 | 3.96 | 4.09 | 4.09 |
| **4.96** | **2.35** | 3.76 | 3.83 | 3.75 | 3.79 | 3.76 | 3.76 | 3.78 | 3.75 | 3.79 | 3.79 |
| **4.96** | **2.09** | 2.76 | 2.77 | 2.75 | 2.77 | 2.77 | 2.76 | 2.76 | 2.76 | 2.77 | 2.76 |
| **4.96** | **1.81** | 2.84 | 2.84 | 2.85 | 2.84 | 2.84 | 2.84 | 2.84 | 2.84 | 2.84 | 2.85 |
| **4.96** | **1.66** | 2.48 | 2.48 | 2.48 | 2.48 | 2.48 | 2.48 | 2.48 | 2.48 | 2.48 | 2.48 |
| **4.96** | **1.23** | 2.97 | 2.97 | 2.97 | 2.97 | 2.97 | 2.97 | 2.97 | 2.97 | 2.97 | 2.97 |
| **4.96** | **1.14** | 3.01 | 3.01 | 3.01 | 3.01 | 3.01 | 3.01 | 3.01 | 3.01 | 3.01 | 3.01 |
| **4.9** | **4.5** | 3.53 | 3.53 | 3.54 | 3.54 | 3.53 | 3.53 | 3.53 | 3.52 | 3.53 | 3.53 |
| **4.9** | **4.38** | 3.38 | 3.4 | 3.39 | 3.37 | 3.38 | 3.38 | 3.38 | 3.37 | 3.38 | 3.38 |
| **4.9** | **4.08** | 3.28 | 3.29 | 3.28 | 3.28 | 3.28 | 3.28 | 3.29 | 3.27 | 3.27 | 3.28 |
| **4.9** | **3.91** | 2.38 | 2.38 | 2.38 | 2.38 | 2.38 | 2.38 | 2.38 | 2.38 | 2.38 | 2.38 |
| **4.9** | **3.84** | 3.91 |  |  | 3.94 | 3.94 | 3.93 | 3.96 | 3.9 | 3.91 | 3.93 |
| **4.9** | **3.39** | 3.17 | 3.2 | 3.18 | 3.19 | 3.19 | 3.19 | 3.18 | 3.18 | 3.18 | 3.17 |
| **4.9** | **3.35** | 2.53 | 2.53 | 2.53 | 2.54 | 2.54 | 2.54 | 2.54 | 2.53 | 2.53 | 2.54 |
| **4.9** | **3.14** |  |  |  |  | **4.34** | **4.3** | **4.31** | **4.31** |  |  |
| **4.9** | **3.08** | 3.8 | 3.85 | 3.82 | 3.8 | 3.83 | 3.81 | 3.83 | 3.84 | 3.8 | 3.79 |
| **4.9** | **3.03** | 3.96 | 3.92 | 3.99 | 3.94 | 3.9 | 3.97 | 4.06 | 3.95 | 4.04 | 4.06 |
| **4.9** | **2.42** | 3.25 | 3.25 | 3.27 | 3.26 | 3.26 | 3.25 | 3.25 | 3.26 | 3.25 | 3.24 |
| **4.9** | **2.35** | 3.21 | 3.15 | 3.17 | 3.15 | 3.07 | 3.07 | 3.08 | 3.17 | 3.09 | 3.08 |
| **4.9** | **1.81** | 4.76 |  |  |  |  |  |  |  |  |  |
| **4.9** | **1.48** |  | **4.09** | **4.09** |  | **4.24** | **4.1** |  | **4.11** | **4.07** | **4.02** |
| **4.5** | **4.38** | 4.46 | 4.35 | 4.17 | 4.08 | 4.31 | 4.16 |  |  | 4.9 |  |
| **4.5** | **4.08** | 3.27 | 3.27 | 3.28 | 3.27 | 3.26 | 3.27 | 3.28 | 3.27 | 3.27 | 3.28 |
| **4.5** | **3.91** | 2.82 | 2.82 | 2.83 | 2.83 | 2.82 | 2.82 | 2.82 | 2.82 | 2.83 | 2.82 |
| **4.5** | **3.84** | 3.03 | 3.03 | 3.03 | 3.04 | 3.03 | 3.04 | 3.03 | 3.04 | 3.04 | 3.04 |
| **4.5** | **3.65** | 2.66 | 2.66 | 2.67 | 2.66 | 2.66 | 2.66 | 2.66 | 2.67 | 2.66 | 2.67 |
| **4.5** | **3.03** | 3.65 | 3.66 | 3.67 | 3.48 | 3.46 | 3.59 | 3.49 | 3.47 | 3.59 | 3.62 |
| **4.5** | **2.28** | 3.39 | 3.39 | 3.39 | 3.39 | 3.38 | 3.4 | 3.39 | 3.39 | 3.39 | 3.39 |
| **4.5** | **2.17** | 2.8 | 2.8 |  | 2.79 | 2.8 | 2.78 | 2.8 | 2.77 | 2.78 | 2.79 |
| **4.5** | **1.91** |  |  |  |  |  |  |  |  |  |  |
| **4.5** | **0.97** |  |  | **4.24** |  |  |  | **4.22** |  |  |  |
| **4.42** | **4.02** | 3.34 | 3.34 | 3.34 | 3.35 | 3.35 | 3.35 | 3.33 | 3.35 | 3.35 | 3.34 |
| **4.42** | **3.91** | 4.06 | 3.95 | 4 | 4.08 | 4.06 | 3.98 | 4 | 4.04 | 4.04 | 4.04 |
| **4.38** | **3.91** | 4.26 | 4.17 | 4.1 | 4.13 | 4.17 | 4.15 | 4.25 | 4.22 | 4.34 | 4.17 |
| **4.38** | **3.84** | 2.28 | 2.28 | 2.28 | 2.28 | 2.28 | 2.28 | 2.28 | 2.28 | 2.28 | 2.28 |
| **4.38** | **3.65** | 4.05 |  |  | 4.08 |  |  |  |  |  |  |
| **4.42** | **3.43** | 3.63 | 3.65 | 3.65 | 3.65 | 3.64 | 3.64 | 3.66 | 3.64 | 3.63 | 3.64 |
| **4.38** | **3.39** | 2.99 | 3.07 | 3 | 3.07 | 3 | 3 | 3 | 3 | 2.99 | 3.06 |
| **4.38** | **3.35** | 3.32 | 3.29 | 3.33 | 3.33 | 3.32 | 3.34 | 3.32 | 3.31 | 3.29 | 3.32 |
| **4.38** | **3.14** | 2.25 | 2.25 | 2.25 | 2.25 | 2.25 | 2.25 | 2.25 | 2.25 | 2.25 | 2.25 |
| **4.38** | **3.08** | 3.16 | 3.16 | 3.16 | 3.17 | 3.17 | 3.16 | 3.15 | 3.17 | 3.16 | 3.16 |
| **4.38** | **3.03** | 3.33 | 3.31 | 3.32 | 3.32 | 3.32 | 3.33 | 3.32 | 3.32 | 3.32 | 3.31 |
| **4.42** | **2.67** | 3.71 | 3.76 |  | 3.73 | 3.67 | 3.72 | 3.71 | 3.73 | 3.68 | 3.71 |
| **4.38** | **2.67** | 3.78 | 3.79 | 3.76 | 3.76 | 3.73 | 3.77 | 3.76 | 3.88 | 3.77 | 3.75 |
| **4.38** | **2.42** |  |  | **3.79** |  | **3.81** | **3.83** | **3.84** | **3.72** | **3.77** | **3.79** |
| **4.38** | **2.35** | 2.41 | 2.4 | 2.41 | 2.41 | 2.4 | 2.41 | 2.41 | 2.41 | 2.41 | 2.41 |
| **4.42** | **2.22** | 3.38 | 3.4 | 3.37 | 3.42 | 3.4 | 3.4 | 3.41 | 3.41 | 3.39 | 3.4 |
| **4.38** | **2.22** | 3.69 | 3.68 | 3.68 | 3.64 | 3.7 | 3.69 | 3.66 | 3.67 | 3.65 | 3.92 |
| **4.42** | **1.91** | 2.59 | 2.59 | 2.59 | 2.59 | 2.59 | 2.59 | 2.59 | 2.59 | 2.59 | 2.59 |
| **4.42** | **1.74** | 3.42 | 3.42 | 3.44 | 3.46 | 3.44 | 3.44 | 3.44 | 3.45 | 3.43 | 3.44 |
| **4.38** | **1.48** | 4.52 |  |  | 4.39 | 4.23 | 4.3 |  |  | 4.5 |  |
| **4.42** | **1.4** | 2.73 | 2.73 | 2.73 | 2.73 | 2.73 | 2.73 | 2.73 | 2.73 | 2.73 | 2.73 |
| **4.38** | **1.14** | 4.08 | 4.13 | 4.14 | 4.18 | 4.15 | 4.14 | 4.12 | 4.13 | 4.11 | 4.12 |
| **4.42** | **0.97** |  |  | **4.06** |  |  |  |  |  |  |  |
| **4.42** | **0.86** | 3.6 | 3.59 | 3.58 | 3.61 | 3.61 | 3.61 | 3.56 | 3.6 | 3.6 | 3.6 |
| **4.08** | **3.91** | 2.28 | 2.28 | 2.28 | 2.28 | 2.28 | 2.28 | 2.28 | 2.28 | 2.28 | 2.28 |
| **4.08** | **1.91** | 4.65 | 4.24 | 4.81 | 4.87 |  | 4.52 |  |  |  | 4.36 |
| **4.08** | **1.14** | 3.69 | 3.74 | 3.71 | 3.78 | 3.71 | 3.74 | 3.7 | 3.72 | 3.7 | 3.72 |
| **4.02** | **3.91** | 2.89 | 2.87 | 2.87 | 2.87 | 2.88 | 2.88 | 2.87 | 2.87 | 2.88 | 2.87 |
| **4.02** | **3.84** | 3.86 | 3.76 | 3.78 | 3.81 | 3.8 | 3.81 | 3.76 | 3.8 | 3.81 | 3.81 |
| **4.02** | **3.65** | 3.6 | 3.56 | 3.58 | 3.58 | 3.58 | 3.57 | 3.57 | 3.55 | 3.58 | 3.61 |
| **4.02** | **3.08** | 3.16 | 3.15 | 3.16 | 3.16 | 3.17 | 3.16 | 3.15 | 3.16 | 3.16 | 3.16 |
| **4.02** | **2.77** | 3.11 | 3.09 | 3.1 | 3.11 | 3.1 | 3.11 | 3.1 | 3.1 | 3.1 | 3.1 |
| **4.02** | **2.67** | 2.92 | 2.92 | 2.91 | 2.92 | 2.91 | 2.92 | 2.92 | 2.92 | 2.92 | 2.91 |
| **4.02** | **2.42** | 3.54 | 3.49 | 3.52 | 3.51 | 3.52 | 3.52 | 3.51 | 3.54 | 3.55 | 3.53 |
| **4.02** | **2.22** | 2.53 | 2.52 | 2.52 | 2.53 | 2.52 | 2.53 | 2.52 | 2.53 | 2.53 | 2.52 |
| **4.02** | **2.17** | 2.41 | 2.42 | 2.41 | 2.4 | 2.41 | 2.4 | 2.41 | 2.4 | 2.4 | 2.41 |
| **4.02** | **1.91** | 2.61 | 2.61 | 2.61 | 2.61 | 2.61 | 2.61 | 2.61 | 2.61 | 2.61 | 2.61 |
| **4.02** | **1.48** | 3.37 | 3.34 | 3.34 | 3.34 | 3.36 | 3.34 | 3.35 | 3.34 | 3.35 | 3.34 |
| **4.02** | **1.3** |  | **4.64** |  |  |  | **4.84** | **4.75** |  | **4.79** | **4.81** |
| **4.02** | **1.14** | 3.36 | 3.34 | 3.35 | 3.36 | 3.36 | 3.35 | 3.34 | 3.35 | 3.36 | 3.35 |
| **4.02** | **1.05** |  |  | **4.35** |  |  |  |  |  | **4.41** |  |
| **4.02** | **0.97** | 3.28 | 3.26 | 3.25 | 3.26 | 3.28 | 3.26 | 3.26 | 3.26 | 3.25 | 3.26 |
| **4.02** | **0.86** | 3.75 | 3.71 | 3.73 | 3.74 | 3.74 | 3.74 | 3.73 | 3.72 | 3.71 | 3.73 |
| **3.91** | **3.35** |  |  | **4.02** |  |  |  |  |  |  |  |
| **3.91** | **3.14** | 3.91 |  | 3.9 | 3.93 | 3.76 |  |  |  |  | 3.82 |
| **3.91** | **3.08** | 3.89 | 3.79 | 3.94 | 3.82 | 3.84 | 3.85 | 3.8 | 3.82 | 3.87 | 3.88 |
| **3.91** | **2.77** | 3.84 | 3.71 | 3.8 | 3.8 | 3.8 | 3.82 | 3.79 | 3.8 | 3.89 | 3.8 |
| **3.91** | **2.42** |  |  |  | **3.77** |  |  |  |  |  |  |
| **3.91** | **2.35** | 3.18 | 3.23 | 3.14 | 3.17 | 3.18 | 3.15 | 3.18 | 3.16 | 3.14 | 3.17 |
| **3.91** | **1.81** | 3.27 | 3.26 | 3.27 | 3.27 | 3.26 | 3.28 | 3.27 | 3.27 | 3.29 | 3.28 |
| **3.91** | **1.66** | 3.75 | 3.68 | 3.86 | 3.76 | 3.84 | 3.72 | 3.7 | 3.7 | 3.73 | 3.88 |
| **3.91** | **1.05** | 3.65 | 3.67 | 3.62 | 3.62 | 3.6 | 3.63 | 3.61 | 3.62 | 3.63 | 3.64 |
| **3.84** | **3.65** | 2.9 | 2.9 | 2.9 | 2.9 | 2.9 | 2.9 | 2.9 | 2.9 | 2.9 | 2.9 |
| **3.84** | **3.39** | 3.51 | 3.52 | 3.5 | 3.5 | 3.49 | 3.48 | 3.49 | 3.51 | 3.49 | 3.49 |
| **3.84** | **3.14** | 3.1 | 3.13 | 3.12 | 3.13 | 3.11 | 3.12 | 3.1 | 3.11 | 3.11 | 3.11 |
| **3.84** | **3.08** | 4.22 | 4.28 |  | 4.3 | 4.32 |  | 4.35 | 4.26 | 4.23 |  |
| **3.84** | **3.03** | 2.91 | 2.91 | 2.91 | 2.91 | 2.91 | 2.91 | 2.91 | 2.91 | 2.91 | 2.91 |
| **3.84** | **2.67** | 2.79 | 2.79 | 2.79 | 2.79 | 2.78 | 2.79 | 2.79 | 2.79 | 2.79 | 2.79 |
| **3.84** | **2.28** | 3.73 | 3.75 | 3.9 | 3.78 | 3.75 | 3.76 | 3.75 | 3.75 | 3.75 | 4.02 |
| **3.84** | **2.22** | 2.78 | 2.78 | 2.79 | 2.78 | 2.77 | 2.78 | 2.78 | 2.78 | 2.78 | 2.78 |
| **3.84** | **2.17** | 2.21 | 2.21 | 2.21 | 2.21 | 2.21 | 2.21 | 2.21 | 2.21 | 2.21 | 2.21 |
| **3.84** | **1.91** | 3.49 | 3.47 | 3.5 | 3.49 | 3.49 | 3.5 | 3.49 | 3.51 | 3.49 | 3.52 |
| **3.84** | **1.48** | 3.41 | 3.31 | 3.42 | 3.43 | 3.32 | 3.44 | 3.31 | 3.31 | 3.31 | 3.44 |
| **3.84** | **0.97** | 3.11 | 3.11 | 3.1 | 3.11 | 3.1 | 3.11 | 3.11 | 3.13 | 3.1 | 3.11 |
| **3.65** | **3.39** | 3.4 | 3.36 | 3.29 | 3.35 | 3.34 | 3.33 | 3.28 | 3.33 | 3.34 | 3.33 |
| **3.65** | **3.03** | 2.35 | 2.35 | 2.35 | 2.35 | 2.35 | 2.35 | 2.35 | 2.35 | 2.35 | 2.35 |
| **3.65** | **2.67** | 3.7 |  |  |  |  |  |  |  |  |  |
| **3.65** | **2.42** | 3.23 | 3.22 | 3.24 | 3.23 | 3.21 | 3.22 | 3.21 | 3.23 | 3.23 | 3.22 |
| **3.65** | **2.28** | 2.55 | 2.54 | 2.55 | 2.55 | 2.55 | 2.54 | 2.55 | 2.55 | 2.55 | 2.54 |
| **3.65** | **2.22** | 3.54 | 3.53 | 3.51 | 3.54 | 3.54 | 3.53 | 3.55 | 3.51 | 3.52 | 3.54 |
| **3.65** | **2.17** | 2.13 | 2.12 | 2.13 | 2.13 | 2.13 | 2.13 | 2.13 | 2.13 | 2.13 | 2.13 |
| **3.65** | **1.91** | 3.66 | 3.65 | 3.63 | 3.69 | 3.73 | 3.63 | 3.77 | 3.72 | 3.6 | 3.59 |
| **3.65** | **1.48** | 2.97 | 2.96 | 2.96 | 2.96 | 2.98 | 2.97 | 2.98 | 2.92 | 2.93 | 2.97 |
| **3.65** | **0.97** | 2.6 | 2.59 | 2.6 | 2.59 | 2.6 | 2.59 | 2.6 | 2.6 | 2.6 | 2.6 |
| **3.39** | **3.14** |  | **4.3** |  | **4.18** | **4.22** | **4.25** | **4.23** | **4.18** | **4.21** | **4.23** |
| **3.39** | **3.03** | 2.48 | 2.48 | 2.48 | 2.48 | 2.47 | 2.48 | 2.48 | 2.48 | 2.48 | 2.48 |
| **3.39** | **2.42** | 3.27 | 3.28 | 3.3 | 3.28 | 3.27 | 3.28 | 3.29 | 3.28 | 3.26 | 3.26 |
| **3.39** | **2.35** | 4.01 |  | 3.9 |  |  |  | 4.03 |  | 3.82 | 3.84 |
| **3.39** | **2.28** | 2.7 | 2.7 | 2.7 | 2.7 | 2.7 | 2.7 | 2.7 | 2.7 | 2.7 | 2.7 |
| **3.43** | **2.13** | 3.54 | 3.54 | 3.51 | 3.54 | 3.54 | 3.53 | 3.53 | 3.52 | 3.53 | 3.54 |
| **3.43** | **2.03** |  |  |  |  |  |  |  |  |  |  |
| **3.43** | **1.4** | 3.69 | 3.64 | 3.65 | 3.63 | 3.65 | 3.67 | 3.65 | 3.68 | 3.68 | 3.69 |
| **3.39** | **0.97** | 3.65 | 3.66 | 3.63 | 3.65 | 3.61 | 3.63 | 3.63 | 3.63 | 3.61 | 3.6 |
| **3.35** | **3.14** | 3.57 | 3.63 | 3.56 | 3.63 | 3.61 | 3.6 | 3.62 | 3.59 | 3.54 | 3.63 |
| **3.35** | **3.08** | 2.65 | 2.65 | 2.65 | 2.65 | 2.65 | 2.65 | 2.64 | 2.65 | 2.64 | 2.64 |
| **3.35** | **2.42** | 2.73 | 2.72 | 2.72 | 2.72 | 2.72 | 2.72 | 2.72 | 2.73 | 2.72 | 2.73 |
| **3.35** | **2.35** | 2.99 | 2.98 | 3.01 | 2.98 | 2.98 | 3 | 3.01 | 3 | 2.99 | 2.98 |
| **3.14** | **3.08** | 2.59 | 2.59 | 2.59 | 2.59 | 2.59 | 2.59 | 2.59 | 2.59 | 2.59 | 2.59 |
| **3.08** | **2.67** | 4.08 | 4.02 | 4 | 4.1 |  | 4 | 4 | 4.23 | 4.07 | 4.11 |
| **3.14** | **2.35** | 2.47 | 2.47 | 2.47 | 2.47 | 2.47 | 2.47 | 2.47 | 2.47 | 2.47 | 2.47 |
| **3.08** | **2.42** | 3.12 | 3.09 | 3.11 | 3.11 | 3.11 | 3.1 | 3.11 | 3.12 | 3.11 | 3.1 |
| **3.14** | **1.81** | 4.03 | 4.2 | 4.24 | 4.32 | 3.88 | 4.21 | 4.16 | 3.96 | 4.24 | 4.23 |
| **3.08** | **1.81** | 4.12 | 4 | 4.22 | 4.09 | 4.02 | 4.12 | 4.2 | 4.14 | 4.12 | 4.09 |
| **3.08** | **1.66** | 3.76 | 3.76 | 3.67 | 3.73 | 3.67 | 3.67 | 3.74 | 3.79 | 3.74 | 3.67 |
| **3.14** | **1.14** | 3.1 | 3.11 | 3.11 | 3.11 | 3.11 | 3.11 | 3.1 | 3.11 | 3.11 | 3.11 |
| **3.08** | **1.14** | 3.15 | 3.16 | 3.16 | 3.17 | 3.16 | 3.15 | 3.16 | 3.16 | 3.16 | 3.16 |
| **3.03** | **2.67** | 4.06 |  |  | 3.99 |  | 4.05 |  |  |  |  |
| **3.03** | **2.42** | 3.39 | 3.52 | 3.39 | 3.43 | 3.39 | 3.4 | 3.42 | 3.41 | 3.42 | 3.42 |
| **3.03** | **2.28** | 2.51 | 2.5 | 2.51 | 2.51 | 2.5 | 2.51 | 2.51 | 2.51 | 2.51 | 2.51 |
| **3.03** | **2.22** | 3.62 |  | 3.64 |  | 3.54 | 3.62 | 3.68 | 3.62 | 3.6 | 3.59 |
| **3.03** | **2.17** | 2.79 | 2.83 | 2.81 | 2.82 | 2.8 | 2.82 | 2.8 | 2.8 | 2.82 | 2.83 |
| **3.03** | **1.91** | 3.77 |  |  |  |  |  |  |  |  |  |
| **3.03** | **1.48** | 2.54 | 2.55 | 2.55 | 2.54 | 2.54 | 2.55 | 2.54 | 2.55 | 2.55 | 2.55 |
| **3.03** | **0.97** | 2.25 | 2.25 | 2.25 | 2.24 | 2.25 | 2.25 | 2.25 | 2.25 | 2.25 | 2.25 |
| **3.03** | **0.86** | 3.85 | 3.81 | 3.79 | 3.89 | 3.84 | 3.87 | 3.77 | 3.82 | 3.75 | 3.75 |
| **2.77** | **2.28** | 3.91 |  |  |  |  |  | 4.3 |  | 4.32 | 4.17 |
| **2.77** | **1.81** | 3.48 | 3.47 | 3.49 | 3.5 | 3.5 | 3.49 | 3.48 | 3.48 | 3.49 | 3.48 |
| **2.77** | **1.66** | 2.85 | 2.85 | 2.85 | 2.85 | 2.86 | 2.85 | 2.86 | 2.85 | 2.86 | 2.85 |
| **2.77** | **1.14** | 3.34 | 3.34 | 3.36 | 3.36 | 3.36 | 3.35 | 3.31 | 3.35 | 3.36 | 3.36 |
| **2.77** | **1.05** | 3.54 | 3.54 | 3.53 | 3.53 | 3.54 | 3.54 | 3.53 | 3.54 | 3.53 | 3.54 |
| **2.67** | **2.22** | 1.86 | 1.86 | 1.86 | 1.86 | 1.86 | 1.86 | 1.86 | 1.86 | 1.86 | 1.86 |
| **2.67** | **2.17** | 2.39 | 2.38 | 2.38 | 2.38 | 2.39 | 2.38 | 2.37 | 2.38 | 2.38 | 2.38 |
| **2.67** | **1.91** | 2.58 | 2.58 | 2.58 | 2.58 | 2.58 | 2.58 | 2.58 | 2.59 | 2.58 | 2.58 |
| **2.67** | **1.48** | 2.77 | 2.77 | 2.77 | 2.77 | 2.76 | 2.77 | 2.77 | 2.77 | 2.77 | 2.76 |
| **2.67** | **0.97** | 3.05 | 3.05 | 3.03 | 3.04 | 3.02 | 3.1 | 3.03 | 3.07 | 3.02 | 3.03 |
| **2.67** | **0.86** |  | **4.37** |  |  |  |  |  |  |  |  |
| **2.42** | **2.35** | 2.8 | 2.98 | 3 | 2.82 | 2.82 | 2.82 | 2.72 | 2.73 | 2.94 | 2.74 |
| **2.42** | **2.28** | 2.2 | 2.2 | 2.2 | 2.2 | 2.2 | 2.2 | 2.2 | 2.2 | 2.2 | 2.2 |
| **2.42** | **1.14** | 3.82 | 3.81 | 3.82 | 3.87 | 3.8 | 3.81 | 3.81 | 3.85 | 3.79 | 3.81 |
| **2.28** | **1.91** | 4.17 | 4.06 |  | 4.16 | 4.24 | 4.05 | 4.45 |  |  |  |
| **2.28** | **0.97** | 3.35 | 3.3 | 3.34 | 3.34 | 3.3 | 3.3 | 3.31 | 3.27 | 3.27 | 3.33 |
| **2.22** | **2.17** | 2.28 | 2.28 | 2.29 | 2.29 | 2.29 | 2.29 | 2.29 | 2.29 | 2.28 | 2.29 |
| **2.22** | **2.13** | 3.41 |  |  |  |  |  |  |  |  |  |
| **2.22** | **2.03** | 3.67 | 3.74 | 3.77 | 3.76 | 3.73 | 3.68 | 3.81 | 3.75 | 3.75 | 3.76 |
| **2.22** | **1.91** | 2.25 | 2.25 | 2.26 | 2.26 | 2.26 | 2.26 | 2.26 | 2.26 | 2.26 | 2.26 |
| **2.17** | **1.91** | 2.79 | 2.81 | 2.78 | 2.78 | 2.78 | 2.78 | 2.77 | 2.81 | 2.8 | 2.8 |
| **2.22** | **1.48** | 2.81 | 2.82 | 2.82 | 2.81 | 2.81 | 2.82 | 2.83 | 2.82 | 2.82 | 2.82 |
| **2.17** | **1.48** | 2.72 | 2.72 | 2.71 | 2.72 | 2.74 | 2.7 | 2.7 | 2.71 | 2.7 | 2.7 |
| **2.22** | **0.97** | 3.11 | 3.13 | 3.12 | 3.04 | 3.11 | 3.12 | 3.11 | 3.09 | 3.09 | 3.1 |
| **2.17** | **0.97** | 2.46 | 2.46 | 2.46 | 2.47 | 2.47 | 2.46 | 2.45 | 2.45 | 2.45 | 2.46 |
| **2.22** | **0.86** | 3.59 | 3.54 | 3.58 | 3.62 | 3.67 | 3.61 | 3.58 | 3.6 | 3.59 | 3.58 |
| **2.13** | **2.03** | 3.35 | 3.32 | 3.3 | 3.31 | 3.31 | 3.3 | 3.31 | 3.31 | 3.32 | 3.31 |
| **2.13** | **1.48** | 4.82 | 4.46 | 4.41 | 4.45 | 4.35 | 4.5 | 4.33 | 4.43 | 4.45 | 4.5 |
| **2.13** | **1.23** |  |  | **5.32** |  |  | **5.28** |  |  |  |  |
| **2.09** | **1.23** | 2.9 | 2.89 | 2.9 | 2.9 | 2.9 | 2.89 | 2.9 | 2.9 | 2.9 | 2.9 |
| **2.13** | **0.97** | 4.38 | 4.1 | 4.22 | 4.06 | 4.14 | 4 | 4.06 | 4.08 | 4.16 | 4.03 |
| **2.03** | **1.74** | 3.98 | 3.91 | 3.9 | 3.92 | 3.92 | 3.93 | 3.99 | 3.94 | 3.91 | 3.91 |
| **2.03** | **1.3** | 3.89 | 3.91 | 3.89 | 3.85 | 3.9 | 3.9 | 3.92 | 3.88 | 3.85 | 3.93 |
| **1.91** | **1.48** | 2.39 | 2.39 | 2.39 | 2.39 | 2.39 | 2.39 | 2.39 | 2.39 | 2.39 | 2.39 |
| **1.91** | **1.4** | 4.15 | 4.03 | 4.12 | 4.08 | 4.12 | 4.12 | 4.13 | 4.24 | 4.13 | 4.13 |
| **1.91** | **0.97** | 2.62 | 2.62 | 2.62 | 2.62 | 2.62 | 2.62 | 2.62 | 2.62 | 2.62 | 2.62 |
| **1.91** | **0.86** | 2.71 | 2.71 | 2.71 | 2.71 | 2.71 | 2.71 | 2.71 | 2.71 | 2.71 | 2.71 |
| **1.81** | **1.66** | 2 | 2 | 2 | 2 | 2 | 2 | 2 | 2 | 2 | 2 |
| **1.81** | **1.14** | 3.56 | 3.59 | 3.58 | 3.6 | 3.57 | 3.58 | 3.57 | 3.57 | 3.56 | 3.57 |
| **1.81** | **1.05** | 2.9 | 2.9 | 2.9 | 2.9 | 2.9 | 2.9 | 2.89 | 2.9 | 2.9 | 2.9 |
| **1.74** | **1.4** | 2.65 | 2.64 | 2.64 | 2.64 | 2.64 | 2.64 | 2.64 | 2.64 | 2.64 | 2.64 |
| **1.74** | **1.3** | 2.78 | 2.89 | 2.88 | 2.89 | 2.88 | 2.89 | 2.77 | 2.89 | 2.89 | 2.89 |
| **1.66** | **1.05** | 3.09 | 3.09 | 3.09 | 3.09 | 3.09 | 3.09 | 3.08 | 3.09 | 3.08 | 3.09 |
| **1.48** | **0.97** | 1.78 | 1.78 | 1.78 | 1.78 | 1.78 | 1.78 | 1.78 | 1.78 | 1.78 | 1.78 |
| **1.48** | **0.86** | 2.66 | 2.65 | 2.66 | 2.66 | 2.65 | 2.66 | 2.66 | 2.66 | 2.66 | 2.65 |
| **1.4** | **1.3** | 2.6 | 2.6 | 2.61 | 2.61 | 2.61 | 2.61 | 2.6 | 2.61 | 2.61 | 2.61 |
| **1.3** | **1.23** | 3.55 | 3.43 | 3.41 | 3.54 | 3.44 | 3.44 | 3.4 | 3.43 | 3.43 | 3.45 |
| **1.09** | **1.05** | 3.23 | 3.19 | 3.2 | 3.19 | 3.2 | 3.2 | 3.18 | 3.21 | 3.2 | 3.19 |
| **0.97** | **0.86** | 2.76 | 2.78 | 2.78 | 2.78 | 2.78 | 2.78 | 2.78 | 2.77 | 2.78 | 2.78 |

Table S11: Interproton distances calculated from US-NUS hybrid schemes 512-50-40 to 512-50-45 sampling schemes as compared to the uniformly sampled reference spectra. Red distances signify a greater than 7% deviation from the uniformly sampled data. Blue distances signify an interproton distance that was not valid in the uniformly sampled data but were valid in the NUS dataset (R^2^ > 0.90, n > 4). Assignments are given as chemical shifts (ppm).

| f2 δ  (ppm) | f1 δ  (ppm) | Ref.  r (Å) | 512-50-40-0  r (Å) | 512-50-40-1  r (Å) | 512-50-40-2  r (Å) | 512-50-42.5-0  r (Å) | 512-50-42.5-1  r (Å) | 512-50-42.5-2  r (Å) | 512-50-45-0  r (Å) | 512-50-45-1  r (Å) | 512-50-45-2  r (Å) |
| --- | --- | --- | --- | --- | --- | --- | --- | --- | --- | --- | --- |
| **9.7** | **6.21** | 4.56 | 4.59 | 4.57 | 4.56 | 4.61 | 4.59 | 4.6 | 4.63 | 4.57 | 4.6 |
| **9.7** | **4.5** | 4.21 | 4.22 | 4.24 | 4.24 | 4.23 | 4.21 | 4.22 | 4.2 | 4.22 | 4.21 |
| **9.7** | **4.38** | 4.21 | 4.2 | 4.21 | 4.22 | 4.2 | 4.18 | 4.21 | 4.18 | 4.19 | 4.18 |
| **9.7** | **4.42** |  |  | **5.86** |  |  |  |  |  |  |  |
| **9.7** | **4.02** | 4.16 | 4.16 | 4.08 | 4.16 | 4.16 | 4.23 | 4.17 | 4.16 | 4.24 | 4.17 |
| **9.7** | **3.84** | 3.01 | 3.02 | 3.02 | 3.01 | 3.02 | 3.02 | 3.01 | 3.02 | 3.01 | 3.02 |
| **9.7** | **3.65** | 4.38 | 4.47 | 4.48 | 4.4 | 4.49 | 4.36 | 4.39 | 4.38 | 4.55 | 4.41 |
| **9.7** | **3.35** | 3.86 | 3.96 | 3.96 | 3.99 | 3.97 | 3.93 | 3.92 | 3.97 | 3.9 | 3.98 |
| **9.7** | **3.14** |  |  |  |  |  |  |  |  |  |  |
| **9.7** | **3.08** |  |  |  |  |  |  |  |  | **4.69** |  |
| **9.7** | **2.67** | 3.23 | 3.24 | 3.24 | 3.24 | 3.24 | 3.24 | 3.24 | 3.24 | 3.25 | 3.24 |
| **9.7** | **2.22** | 3.19 | 3.21 | 3.21 | 3.2 | 3.2 | 3.2 | 3.21 | 3.2 | 3.2 | 3.2 |
| **9.7** | **2.17** | 3 | 3.04 | 3.05 | 3.04 | 3.02 | 3.03 | 3.03 | 3.03 | 3.03 | 3.05 |
| **9.7** | **1.91** | 3.75 | 3.79 | 3.78 | 3.8 | 3.78 | 3.78 | 3.8 | 3.76 | 3.81 | 3.82 |
| **9.7** | **1.48** | 3.61 | 3.63 | 3.63 | 3.63 | 3.64 | 3.62 | 3.63 | 3.63 | 3.65 | 3.66 |
| **9.7** | **1.14** | 3.89 | 3.93 | 3.93 | 3.91 | 3.9 | 3.91 | 3.92 | 3.9 | 3.9 | 3.92 |
| **6.21** | **6.05** | 2.97 | 2.96 | 2.97 | 2.97 | 2.97 | 2.97 | 2.97 | 2.96 | 2.97 | 2.97 |
| **6.21** | **5.57** | 2.53 | 2.53 | 2.53 | 2.53 | 2.53 | 2.53 | 2.53 | 2.53 | 2.53 | 2.53 |
| **6.21** | **4.5** | 3.67 | 3.66 | 3.65 | 3.66 | 3.66 | 3.68 | 3.67 | 3.67 | 3.66 | 3.66 |
| **6.21** | **4.42** | 4.21 | 4.2 | 4.14 | 4.15 | 4.17 | 4.26 | 4.23 | 4.17 | 4.16 | 4.18 |
| **6.21** | **4.02** | 2.53 | 2.53 | 2.53 | 2.53 | 2.53 | 2.53 | 2.53 | 2.54 | 2.53 | 2.53 |
| **6.21** | **3.91** |  |  |  |  |  |  | **4.89** | **4.87** | **4.8** |  |
| **6.21** | **3.84** | 3.85 | 3.84 | 3.83 | 3.83 | 3.83 | 3.87 | 3.86 | 3.85 | 3.84 | 3.84 |
| **6.21** | **3.65** | 2.84 | 2.84 | 2.84 | 2.84 | 2.84 | 2.85 | 2.84 | 2.84 | 2.84 | 2.84 |
| **6.21** | **3.03** | 3.85 | 3.93 | 3.93 | 3.93 | 3.94 | 3.87 | 3.86 | 3.95 | 3.93 | 3.94 |
| **6.21** | **2.67** | 4.17 | 3.86 | 3.86 | 3.86 | 3.87 | 4.19 | 4.18 | 3.9 | 3.88 | 3.89 |
| **6.21** | **2.22** | 3.74 | 3.75 | 3.75 | 3.75 | 3.74 | 3.76 | 3.76 | 3.75 | 3.75 | 3.73 |
| **6.21** | **2.17** | 2.38 | 2.38 | 2.38 | 2.38 | 2.38 | 2.38 | 2.38 | 2.37 | 2.38 | 2.37 |
| **6.21** | **2.09** | 3.73 | 3.7 | 3.73 | 3.72 | 3.72 | 3.72 | 3.75 | 3.72 | 3.75 | 3.73 |
| **6.21** | **1.91** | 3.47 | 3.47 | 3.46 | 3.46 | 3.46 | 3.48 | 3.47 | 3.47 | 3.46 | 3.46 |
| **6.21** | **1.48** | 4.15 | 3.95 | 3.96 | 3.91 | 3.98 | 4.02 | 4.02 | 3.98 | 3.97 | 3.99 |
| **6.21** | **0.97** | 3.38 | 3.37 | 3.36 | 3.36 | 3.36 | 3.37 | 3.36 | 3.37 | 3.37 | 3.37 |
| **6.21** | **0.86** | 4.05 |  |  | 4.08 | 4.04 | 4.24 | 4.19 | 4.06 | 4.04 | 4.1 |
| **6.05** | **5.57** | 2.49 | 2.49 | 2.49 | 2.49 | 2.49 | 2.49 | 2.49 | 2.5 | 2.49 | 2.49 |
| **6.05** | **4.96** | 4.02 | 4.01 | 4.04 | 4.02 | 4.03 | 4.04 | 4.04 | 4.03 | 4.03 | 4.02 |
| **6.05** | **4.02** | 3.91 | 3.91 | 3.93 | 3.9 | 3.9 | 3.9 | 3.89 | 3.92 | 3.91 | 3.89 |
| **6.05** | **3.65** | 3.43 | 3.43 | 3.43 | 3.43 | 3.43 | 3.44 | 3.43 | 3.43 | 3.43 | 3.43 |
| **6.05** | **2.28** | 3.98 | 3.99 | 3.99 | 3.99 | 3.99 | 3.99 | 3.99 | 3.99 | 4 | 4.01 |
| **6.05** | **2.09** | 2.3 | 2.3 | 2.31 | 2.3 | 2.31 | 2.31 | 2.3 | 2.3 | 2.31 | 2.3 |
| **6.05** | **1.91** |  |  |  |  | **4.91** |  |  |  | **5.05** |  |
| **6.05** | **1.23** | 4.55 | 4.56 | 4.51 | 4.56 | 4.52 | 4.56 | 4.55 | 4.56 | 4.52 | 4.54 |
| **6.05** | **0.97** | 3.52 | 3.53 | 3.54 | 3.52 | 3.52 | 3.52 | 3.51 | 3.52 | 3.52 | 3.52 |
| **6.05** | **0.86** | 4.03 | 4.04 | 4.04 | 4.01 | 3.99 | 4.01 | 3.99 | 4.03 | 4.04 | 4.03 |
| **5.57** | **4.96** | 2.99 | 2.99 | 2.99 | 2.99 | 2.99 | 2.99 | 2.99 | 2.99 | 2.99 | 2.99 |
| **5.57** | **4.5** | 3.86 | 3.85 | 3.86 | 3.85 | 3.85 | 3.86 | 3.84 | 3.84 | 3.84 | 3.85 |
| **5.57** | **4.42** |  | **4.53** |  |  |  |  |  |  |  |  |
| **5.57** | **4.02** | 3.31 | 3.3 | 3.3 | 3.3 | 3.3 | 3.3 | 3.31 | 3.3 | 3.3 | 3.3 |
| **5.57** | **3.65** | 3.25 | 3.25 | 3.25 | 3.25 | 3.25 | 3.25 | 3.25 | 3.25 | 3.24 | 3.25 |
| **5.57** | **3.03** | 3.76 | 3.75 | 3.76 | 3.75 | 3.75 | 3.76 | 3.75 | 3.74 | 3.74 | 3.76 |
| **5.57** | **2.28** | 4.41 | 4.37 | 4.38 | 4.32 | 4.37 | 4.38 | 4.33 | 4.29 | 4.49 | 4.34 |
| **5.57** | **2.17** | 3.54 | 3.43 | 3.46 | 3.5 | 3.5 | 3.49 | 3.43 |  |  | 3.43 |
| **5.57** | **2.09** | 2.71 | 2.8 | 2.81 | 2.8 | 2.8 | 2.8 | 2.8 | 2.79 | 2.8 | 2.8 |
| **5.57** | **1.91** | 3.61 | 3.61 | 3.61 | 3.61 | 3.61 | 3.62 | 3.61 | 3.6 | 3.61 | 3.6 |
| **5.57** | **1.48** | 3.22 | 3.23 | 3.23 | 3.23 | 3.22 | 3.23 | 3.23 | 3.22 | 3.23 | 3.22 |
| **5.57** | **1.23** | 4.18 | 4.22 | 4.22 | 4.21 | 4.2 | 4.23 | 4.24 | 4.22 | 4.23 | 4.21 |
| **5.57** | **0.97** | 2.77 | 2.77 | 2.77 | 2.77 | 2.77 | 2.77 | 2.77 | 2.77 | 2.77 | 2.77 |
| **5.57** | **0.86** | 2.87 | 2.87 | 2.87 | 2.87 | 2.87 | 2.87 | 2.87 | 2.87 | 2.87 | 2.87 |
| **4.96** | **4.5** |  |  |  | **4.66** |  |  | **4.73** |  | **4.66** |  |
| **4.96** | **4.02** |  |  | **4.33** |  | **4.3** |  | **4.28** | **4.36** |  |  |
| **4.96** | **3.91** | 4.14 | 3.79 | 4.1 | 4.17 | 3.78 | 3.8 | 4.17 | 4.12 | 3.82 | 3.84 |
| **4.96** | **3.65** |  |  |  |  |  |  | **4.33** |  |  |  |
| **4.96** | **3.35** | 4.38 |  |  |  |  |  |  |  |  |  |
| **4.96** | **3.14** | 3.59 | 3.6 | 3.61 | 3.6 | 3.61 | 3.61 | 3.6 | 3.61 | 3.62 | 3.62 |
| **4.96** | **3.08** | 2.72 | 2.73 | 2.73 | 2.72 | 2.72 | 2.73 | 2.72 | 2.72 | 2.73 | 2.72 |
| **4.96** | **2.77** | 4.05 | 4.11 | 4.09 | 4.07 | 4.1 | 4.08 | 4.06 | 4.11 | 4.01 | 4.07 |
| **4.96** | **2.35** | 3.76 | 3.77 | 3.79 | 3.77 | 3.77 | 3.76 | 3.78 | 3.79 | 3.8 | 3.77 |
| **4.96** | **2.09** | 2.76 | 2.77 | 2.77 | 2.77 | 2.77 | 2.77 | 2.77 | 2.77 | 2.77 | 2.77 |
| **4.96** | **1.81** | 2.84 | 2.85 | 2.85 | 2.84 | 2.84 | 2.84 | 2.84 | 2.85 | 2.85 | 2.84 |
| **4.96** | **1.66** | 2.48 | 2.48 | 2.48 | 2.48 | 2.48 | 2.48 | 2.48 | 2.48 | 2.48 | 2.48 |
| **4.96** | **1.23** | 2.97 | 2.97 | 2.97 | 2.97 | 2.97 | 2.97 | 2.97 | 2.97 | 2.97 | 2.97 |
| **4.96** | **1.14** | 3.01 | 3.01 | 3.01 | 3.01 | 3.01 | 3.01 | 3.01 | 3.01 | 3.01 | 3.01 |
| **4.9** | **4.5** | 3.53 | 3.53 | 3.53 | 3.53 | 3.54 | 3.54 | 3.54 | 3.54 | 3.54 | 3.53 |
| **4.9** | **4.38** | 3.38 | 3.37 | 3.37 | 3.37 | 3.38 | 3.38 | 3.38 | 3.38 | 3.37 | 3.38 |
| **4.9** | **4.08** | 3.28 | 3.27 | 3.28 | 3.27 | 3.28 | 3.29 | 3.29 | 3.28 | 3.28 | 3.26 |
| **4.9** | **3.91** | 2.38 | 2.38 | 2.38 | 2.38 | 2.38 | 2.38 | 2.38 | 2.38 | 2.38 | 2.38 |
| **4.9** | **3.84** | 3.91 | 3.96 | 3.95 | 3.89 | 3.95 | 3.95 | 3.95 | 3.94 | 3.91 | 3.93 |
| **4.9** | **3.39** | 3.17 | 3.18 | 3.19 | 3.18 | 3.18 | 3.18 | 3.19 | 3.18 | 3.17 | 3.18 |
| **4.9** | **3.35** | 2.53 | 2.54 | 2.54 | 2.54 | 2.54 | 2.54 | 2.54 | 2.54 | 2.54 | 2.54 |
| **4.9** | **3.14** |  | **4.27** | **4.06** |  | **4.31** |  | **4.1** |  | **4.02** |  |
| **4.9** | **3.08** | 3.8 | 3.82 | 3.81 | 3.82 | 3.81 | 3.8 | 3.81 | 3.85 | 3.83 | 3.8 |
| **4.9** | **3.03** | 3.96 | 3.98 | 3.98 | 3.96 | 4.08 | 3.98 | 4.08 | 4.01 | 3.96 | 4.01 |
| **4.9** | **2.42** | 3.25 | 3.25 | 3.25 | 3.25 | 3.26 | 3.24 | 3.25 | 3.24 | 3.25 | 3.24 |
| **4.9** | **2.35** | 3.21 | 3.09 | 3.1 | 3.16 | 3.09 | 3.08 | 3.09 | 3.08 | 3.16 | 3.17 |
| **4.9** | **1.81** | 4.76 |  |  |  |  |  |  |  |  |  |
| **4.9** | **1.48** |  | **4.08** | **4.04** | **4.08** | **4.12** | **4.03** | **4.1** |  | **4.1** | **4.09** |
| **4.5** | **4.38** | 4.46 | 4.79 | 4.4 |  | 4.14 |  | 4.54 |  |  |  |
| **4.5** | **4.08** | 3.27 | 3.26 | 3.27 | 3.27 | 3.29 | 3.26 | 3.28 | 3.26 | 3.27 | 3.27 |
| **4.5** | **3.91** | 2.82 | 2.82 | 2.82 | 2.83 | 2.83 | 2.82 | 2.83 | 2.82 | 2.83 | 2.83 |
| **4.5** | **3.84** | 3.03 | 3.04 | 3.04 | 3.04 | 3.04 | 3.04 | 3.04 | 3.04 | 3.04 | 3.04 |
| **4.5** | **3.65** | 2.66 | 2.66 | 2.67 | 2.66 | 2.67 | 2.67 | 2.66 | 2.67 | 2.66 | 2.67 |
| **4.5** | **3.03** | 3.65 | 3.59 | 3.47 | 3.58 | 3.48 | 3.61 | 3.59 | 3.58 | 3.58 | 3.6 |
| **4.5** | **2.28** | 3.39 | 3.38 | 3.39 | 3.38 | 3.41 | 3.39 | 3.38 | 3.39 | 3.39 | 3.39 |
| **4.5** | **2.17** | 2.8 | 2.78 | 2.78 | 2.8 | 2.8 |  | 2.78 | 2.77 | 2.78 | 2.78 |
| **4.5** | **1.91** |  |  |  |  | **4.74** |  |  |  |  |  |
| **4.5** | **0.97** |  |  |  |  |  |  |  |  |  |  |
| **4.42** | **4.02** | 3.34 | 3.35 | 3.34 | 3.34 | 3.34 | 3.34 | 3.35 | 3.34 | 3.34 | 3.34 |
| **4.42** | **3.91** | 4.06 | 3.98 | 4.02 | 4.1 | 4.02 | 4.07 | 4.08 | 4 | 3.99 | 4.06 |
| **4.38** | **3.91** | 4.26 | 4.4 | 4.26 | 4.32 | 4.23 | 4.27 | 4.29 | 4.41 | 4.27 | 4.26 |
| **4.38** | **3.84** | 2.28 | 2.28 | 2.28 | 2.28 | 2.28 | 2.28 | 2.28 | 2.28 | 2.28 | 2.28 |
| **4.38** | **3.65** | 4.05 |  |  | 4.01 | 4.02 |  | 4.08 | 4.07 |  | 4.03 |
| **4.42** | **3.43** | 3.63 | 3.66 | 3.65 | 3.64 | 3.65 | 3.66 | 3.65 | 3.64 | 3.64 | 3.65 |
| **4.38** | **3.39** | 2.99 | 2.99 | 3 | 2.99 | 3 | 2.99 | 3 | 3 | 2.99 | 3 |
| **4.38** | **3.35** | 3.32 | 3.33 | 3.33 | 3.33 | 3.34 | 3.33 | 3.36 | 3.35 | 3.33 | 3.33 |
| **4.38** | **3.14** | 2.25 | 2.25 | 2.25 | 2.25 | 2.25 | 2.25 | 2.25 | 2.25 | 2.25 | 2.25 |
| **4.38** | **3.08** | 3.16 | 3.17 | 3.16 | 3.16 | 3.16 | 3.16 | 3.16 | 3.18 | 3.16 | 3.16 |
| **4.38** | **3.03** | 3.33 | 3.33 | 3.32 | 3.31 | 3.32 | 3.32 | 3.34 | 3.33 | 3.31 | 3.33 |
| **4.42** | **2.67** | 3.71 | 3.67 | 3.72 | 3.73 | 3.73 | 3.72 | 3.73 |  | 3.71 | 3.68 |
| **4.38** | **2.67** | 3.78 | 3.78 | 3.76 | 3.77 | 3.84 | 3.76 | 3.79 | 3.9 | 3.74 | 3.77 |
| **4.38** | **2.42** |  | **3.71** | **3.72** | **3.8** | **3.76** | **3.68** |  | **3.77** | **3.77** | **3.78** |
| **4.38** | **2.35** | 2.41 | 2.41 | 2.41 | 2.41 | 2.41 | 2.41 | 2.41 | 2.41 | 2.41 | 2.41 |
| **4.42** | **2.22** | 3.38 | 3.39 | 3.4 | 3.41 | 3.4 | 3.41 | 3.4 | 3.38 | 3.38 | 3.37 |
| **4.38** | **2.22** | 3.69 | 3.93 | 3.62 | 3.64 | 3.65 | 3.66 | 3.66 | 3.66 | 3.64 | 3.61 |
| **4.42** | **1.91** | 2.59 | 2.59 | 2.59 | 2.59 | 2.59 | 2.59 | 2.59 | 2.59 | 2.59 | 2.59 |
| **4.42** | **1.74** | 3.42 | 3.44 | 3.44 | 3.45 | 3.44 | 3.41 | 3.41 | 3.41 | 3.42 | 3.42 |
| **4.38** | **1.48** | 4.52 |  | 4.48 |  |  | 4.56 | 4.41 |  |  |  |
| **4.42** | **1.4** | 2.73 | 2.73 | 2.73 | 2.73 | 2.73 | 2.73 | 2.73 | 2.73 | 2.73 | 2.73 |
| **4.38** | **1.14** | 4.08 | 4.11 | 4.1 | 4.11 | 4.11 | 4.13 | 4.12 | 4.13 | 4.1 | 4.1 |
| **4.42** | **0.97** |  |  |  |  |  |  |  |  |  |  |
| **4.42** | **0.86** | 3.6 | 3.6 | 3.61 | 3.6 | 3.61 | 3.61 | 3.61 | 3.6 | 3.61 | 3.61 |
| **4.08** | **3.91** | 2.28 | 2.28 | 2.28 | 2.28 | 2.28 | 2.28 | 2.29 | 2.28 | 2.28 | 2.28 |
| **4.08** | **1.91** | 4.65 | 4.64 | 4.73 | 4.74 | 4.75 | 4.89 | 4.96 | 4.85 | 4.73 |  |
| **4.08** | **1.14** | 3.69 | 3.69 | 3.69 | 3.7 | 3.71 | 3.72 | 3.72 | 3.72 | 3.7 | 3.72 |
| **4.02** | **3.91** | 2.89 | 2.87 | 2.88 | 2.87 | 2.87 | 2.89 | 2.87 | 2.88 | 2.87 | 2.87 |
| **4.02** | **3.84** | 3.86 | 3.83 | 3.81 | 3.81 | 3.8 | 3.79 | 3.78 | 3.82 | 3.81 | 3.81 |
| **4.02** | **3.65** | 3.6 | 3.61 | 3.57 | 3.57 | 3.59 | 3.58 | 3.59 | 3.57 | 3.56 | 3.56 |
| **4.02** | **3.08** | 3.16 | 3.16 | 3.15 | 3.15 | 3.16 | 3.16 | 3.15 | 3.17 | 3.16 | 3.16 |
| **4.02** | **2.77** | 3.11 | 3.11 | 3.1 | 3.1 | 3.11 | 3.09 | 3.1 | 3.11 | 3.1 | 3.1 |
| **4.02** | **2.67** | 2.92 | 2.92 | 2.91 | 2.92 | 2.91 | 2.91 | 2.92 | 2.92 | 2.91 | 2.91 |
| **4.02** | **2.42** | 3.54 | 3.54 | 3.54 | 3.55 | 3.54 | 3.54 | 3.53 | 3.54 | 3.53 | 3.51 |
| **4.02** | **2.22** | 2.53 | 2.53 | 2.53 | 2.52 | 2.53 | 2.53 | 2.53 | 2.52 | 2.53 | 2.52 |
| **4.02** | **2.17** | 2.41 | 2.4 | 2.4 | 2.41 | 2.4 | 2.4 | 2.4 | 2.41 | 2.4 | 2.41 |
| **4.02** | **1.91** | 2.61 | 2.61 | 2.61 | 2.61 | 2.61 | 2.61 | 2.61 | 2.61 | 2.61 | 2.61 |
| **4.02** | **1.48** | 3.37 | 3.36 | 3.34 | 3.33 | 3.35 | 3.33 | 3.34 | 3.35 | 3.34 | 3.34 |
| **4.02** | **1.3** |  |  | **4.82** |  |  | **4.87** |  |  |  |  |
| **4.02** | **1.14** | 3.36 | 3.35 | 3.35 | 3.35 | 3.36 | 3.36 | 3.35 | 3.35 | 3.35 | 3.35 |
| **4.02** | **1.05** |  |  |  |  |  |  |  |  |  |  |
| **4.02** | **0.97** | 3.28 | 3.28 | 3.25 | 3.25 | 3.26 | 3.26 | 3.25 | 3.26 | 3.25 | 3.25 |
| **4.02** | **0.86** | 3.75 | 3.75 | 3.73 | 3.73 | 3.74 | 3.74 | 3.74 | 3.74 | 3.74 | 3.74 |
| **3.91** | **3.35** |  |  |  |  |  |  |  |  |  |  |
| **3.91** | **3.14** | 3.91 | 3.81 | 3.83 | 3.87 | 3.86 | 3.88 |  | 3.83 | 3.83 |  |
| **3.91** | **3.08** | 3.89 | 3.82 | 3.81 | 3.84 | 3.82 | 3.85 | 3.82 | 3.8 | 3.88 | 3.9 |
| **3.91** | **2.77** | 3.84 | 3.81 | 3.81 | 3.82 | 3.83 | 3.79 | 3.83 | 3.81 | 3.81 | 3.83 |
| **3.91** | **2.42** |  |  |  |  |  |  |  |  |  |  |
| **3.91** | **2.35** | 3.18 | 3.18 | 3.18 | 3.17 | 3.19 | 3.18 | 3.19 | 3.19 | 3.18 | 3.19 |
| **3.91** | **1.81** | 3.27 | 3.27 | 3.27 | 3.26 | 3.26 | 3.28 | 3.27 | 3.27 | 3.28 | 3.28 |
| **3.91** | **1.66** | 3.75 | 3.88 | 3.69 | 3.72 | 3.72 | 3.73 |  | 3.91 | 3.92 |  |
| **3.91** | **1.05** | 3.65 | 3.62 | 3.63 | 3.64 | 3.63 | 3.63 | 3.64 | 3.6 | 3.61 | 3.64 |
| **3.84** | **3.65** | 2.9 | 2.9 | 2.9 | 2.9 | 2.9 | 2.9 | 2.9 | 2.9 | 2.9 | 2.91 |
| **3.84** | **3.39** | 3.51 | 3.49 | 3.51 | 3.5 | 3.5 | 3.5 | 3.51 | 3.49 | 3.48 | 3.5 |
| **3.84** | **3.14** | 3.1 | 3.12 | 3.11 | 3.12 | 3.12 | 3.12 | 3.11 | 3.12 | 3.12 | 3.12 |
| **3.84** | **3.08** | 4.22 | 4.33 | 4.24 | 4.27 | 4.27 | 4.27 | 4.29 | 4.3 | 4.28 | 4.31 |
| **3.84** | **3.03** | 2.91 | 2.92 | 2.92 | 2.92 | 2.92 | 2.92 | 2.91 | 2.92 | 2.92 | 2.92 |
| **3.84** | **2.67** | 2.79 | 2.79 | 2.79 | 2.79 | 2.79 | 2.79 | 2.79 | 2.79 | 2.79 | 2.79 |
| **3.84** | **2.28** | 3.73 | 3.95 | 3.94 | 3.95 | 3.95 | 3.75 | 3.78 | 3.94 | 3.93 | 3.97 |
| **3.84** | **2.22** | 2.78 | 2.78 | 2.78 | 2.78 | 2.78 | 2.78 | 2.78 | 2.78 | 2.78 | 2.77 |
| **3.84** | **2.17** | 2.21 | 2.21 | 2.21 | 2.21 | 2.21 | 2.21 | 2.21 | 2.21 | 2.21 | 2.21 |
| **3.84** | **1.91** | 3.49 | 3.53 | 3.5 | 3.5 | 3.54 | 3.5 | 3.51 | 3.52 | 3.5 | 3.51 |
| **3.84** | **1.48** | 3.41 | 3.45 | 3.42 | 3.31 | 3.32 | 3.3 | 3.31 | 3.33 | 3.32 | 3.31 |
| **3.84** | **0.97** | 3.11 | 3.1 | 3.11 | 3.1 | 3.11 | 3.1 | 3.14 | 3.12 | 3.13 | 3.11 |
| **3.65** | **3.39** | 3.4 | 3.35 | 3.35 | 3.29 | 3.35 | 3.36 | 3.38 | 3.35 | 3.33 | 3.35 |
| **3.65** | **3.03** | 2.35 | 2.35 | 2.35 | 2.35 | 2.35 | 2.35 | 2.36 | 2.35 | 2.35 | 2.35 |
| **3.65** | **2.67** | 3.7 |  |  |  |  |  |  |  |  |  |
| **3.65** | **2.42** | 3.23 | 3.23 | 3.22 | 3.24 | 3.22 | 3.22 | 3.23 | 3.22 | 3.22 | 3.24 |
| **3.65** | **2.28** | 2.55 | 2.54 | 2.55 | 2.55 | 2.54 | 2.55 | 2.55 | 2.54 | 2.55 | 2.55 |
| **3.65** | **2.22** | 3.54 | 3.54 | 3.55 | 3.53 | 3.53 | 3.54 | 3.53 | 3.53 | 3.55 | 3.54 |
| **3.65** | **2.17** | 2.13 | 2.13 | 2.13 | 2.13 | 2.13 | 2.13 | 2.13 | 2.13 | 2.13 | 2.13 |
| **3.65** | **1.91** | 3.66 | 3.76 | 3.63 | 3.74 | 3.75 | 3.77 | 3.65 | 3.74 | 3.59 | 3.68 |
| **3.65** | **1.48** | 2.97 | 2.97 | 2.96 | 2.92 | 2.96 | 2.97 | 2.97 | 2.96 | 2.97 | 2.97 |
| **3.65** | **0.97** | 2.6 | 2.59 | 2.6 | 2.6 | 2.59 | 2.6 | 2.6 | 2.6 | 2.59 | 2.6 |
| **3.39** | **3.14** |  | **4.26** | **4.08** | **4.37** | **4.25** |  |  | **4.17** | **4.15** | **4.1** |
| **3.39** | **3.03** | 2.48 | 2.48 | 2.48 | 2.48 | 2.48 | 2.48 | 2.48 | 2.48 | 2.47 | 2.48 |
| **3.39** | **2.42** | 3.27 | 3.27 | 3.26 | 3.26 | 3.26 | 3.25 | 3.26 | 3.26 | 3.26 | 3.27 |
| **3.39** | **2.35** | 4.01 |  | 4.12 |  |  |  | 4.13 | 4.06 |  |  |
| **3.39** | **2.28** | 2.7 | 2.7 | 2.7 | 2.7 | 2.7 | 2.7 | 2.7 | 2.71 | 2.7 | 2.71 |
| **3.43** | **2.13** | 3.54 | 3.55 | 3.52 | 3.53 | 3.54 | 3.53 | 3.53 | 3.53 | 3.54 | 3.54 |
| **3.43** | **2.03** |  |  | **4.59** |  |  |  |  |  |  |  |
| **3.43** | **1.4** | 3.69 | 3.67 | 3.65 | 3.67 | 3.66 | 3.68 | 3.68 | 3.69 | 3.71 | 3.67 |
| **3.39** | **0.97** | 3.65 | 3.6 | 3.64 | 3.63 | 3.61 | 3.62 | 3.64 | 3.63 | 3.63 | 3.61 |
| **3.35** | **3.14** | 3.57 | 3.63 | 3.65 | 3.65 | 3.62 | 3.62 | 3.63 | 3.65 | 3.66 | 3.71 |
| **3.35** | **3.08** | 2.65 | 2.65 | 2.65 | 2.65 | 2.65 | 2.65 | 2.65 | 2.65 | 2.65 | 2.65 |
| **3.35** | **2.42** | 2.73 | 2.73 | 2.73 | 2.73 | 2.73 | 2.73 | 2.73 | 2.73 | 2.73 | 2.73 |
| **3.35** | **2.35** | 2.99 | 3 | 3.01 | 2.98 | 2.99 | 2.99 | 3.03 | 2.99 | 3.01 | 3 |
| **3.14** | **3.08** | 2.59 | 2.6 | 2.59 | 2.6 | 2.6 | 2.6 | 2.6 | 2.6 | 2.6 | 2.6 |
| **3.08** | **2.67** | 4.08 |  | 4 | 4.17 | 4.04 |  |  | 3.99 | 4.05 | 4.08 |
| **3.14** | **2.35** | 2.47 | 2.47 | 2.47 | 2.47 | 2.47 | 2.47 | 2.47 | 2.47 | 2.47 | 2.47 |
| **3.08** | **2.42** | 3.12 | 3.12 | 3.11 | 3.12 | 3.1 | 3.11 | 3.11 | 3.11 | 3.11 | 3.11 |
| **3.14** | **1.81** | 4.03 | 4.02 | 3.89 |  | 4.26 | 4 | 3.93 | 4.23 | 4.01 | 3.99 |
| **3.08** | **1.81** | 4.12 | 4.17 | 4.12 | 4.12 | 4.07 | 4.15 | 4.17 | 4.08 | 4.17 | 4.21 |
| **3.08** | **1.66** | 3.76 | 3.76 | 3.74 | 3.55 | 3.53 | 3.66 | 3.53 | 3.77 | 3.73 | 3.68 |
| **3.14** | **1.14** | 3.1 | 3.11 | 3.1 | 3.11 | 3.11 | 3.11 | 3.11 | 3.11 | 3.11 | 3.11 |
| **3.08** | **1.14** | 3.15 | 3.15 | 3.15 | 3.15 | 3.16 | 3.16 | 3.15 | 3.16 | 3.16 | 3.16 |
| **3.03** | **2.67** | 4.06 |  |  |  |  |  |  |  |  |  |
| **3.03** | **2.42** | 3.39 | 3.39 | 3.39 | 3.4 | 3.38 | 3.4 | 3.4 | 3.41 | 3.38 | 3.41 |
| **3.03** | **2.28** | 2.51 | 2.51 | 2.51 | 2.51 | 2.51 | 2.51 | 2.51 | 2.51 | 2.51 | 2.51 |
| **3.03** | **2.22** | 3.62 | 3.59 | 3.6 | 3.58 | 3.58 | 3.62 | 3.6 | 3.57 | 3.55 | 3.74 |
| **3.03** | **2.17** | 2.79 | 2.79 | 2.79 | 2.79 | 2.79 | 2.79 | 2.79 | 2.77 | 2.78 | 2.79 |
| **3.03** | **1.91** | 3.77 | 3.95 |  | 3.76 |  |  |  | 3.8 |  |  |
| **3.03** | **1.48** | 2.54 | 2.54 | 2.54 | 2.54 | 2.55 | 2.55 | 2.55 | 2.55 | 2.55 | 2.55 |
| **3.03** | **0.97** | 2.25 | 2.25 | 2.25 | 2.25 | 2.25 | 2.25 | 2.25 | 2.25 | 2.25 | 2.25 |
| **3.03** | **0.86** | 3.85 | 3.86 | 3.86 | 3.83 | 3.86 | 3.77 | 3.86 | 3.85 | 3.76 | 3.9 |
| **2.77** | **2.28** | 3.91 | 4.08 | 4.14 | 4.27 |  |  | 4.29 |  | 4.37 | 4.08 |
| **2.77** | **1.81** | 3.48 | 3.49 | 3.49 | 3.47 | 3.5 | 3.47 | 3.49 | 3.48 | 3.48 | 3.47 |
| **2.77** | **1.66** | 2.85 | 2.85 | 2.85 | 2.85 | 2.86 | 2.85 | 2.85 | 2.86 | 2.85 | 2.85 |
| **2.77** | **1.14** | 3.34 | 3.34 | 3.34 | 3.35 | 3.35 | 3.36 | 3.35 | 3.36 | 3.35 | 3.36 |
| **2.77** | **1.05** | 3.54 | 3.54 | 3.54 | 3.54 | 3.55 | 3.53 | 3.54 | 3.53 | 3.54 | 3.52 |
| **2.67** | **2.22** | 1.86 | 1.86 | 1.86 | 1.86 | 1.86 | 1.86 | 1.86 | 1.86 | 1.86 | 1.86 |
| **2.67** | **2.17** | 2.39 | 2.38 | 2.37 | 2.38 | 2.39 | 2.38 | 2.38 | 2.37 | 2.38 | 2.38 |
| **2.67** | **1.91** | 2.58 | 2.58 | 2.58 | 2.58 | 2.58 | 2.58 | 2.58 | 2.58 | 2.58 | 2.58 |
| **2.67** | **1.48** | 2.77 | 2.77 | 2.76 | 2.76 | 2.76 | 2.76 | 2.76 | 2.76 | 2.77 | 2.77 |
| **2.67** | **0.97** | 3.05 | 3.02 | 3.08 | 3.04 | 3.07 | 3.02 | 3.09 | 3.08 | 3.01 | 3.03 |
| **2.67** | **0.86** |  |  |  |  |  |  |  |  |  |  |
| **2.42** | **2.35** | 2.8 | 2.74 | 2.97 | 2.73 | 2.73 | 2.73 | 3 | 2.73 | 2.82 | 2.75 |
| **2.42** | **2.28** | 2.2 | 2.2 | 2.2 | 2.2 | 2.2 | 2.2 | 2.2 | 2.2 | 2.2 | 2.2 |
| **2.42** | **1.14** | 3.82 | 3.81 | 3.82 | 3.81 | 3.79 | 3.83 | 3.92 | 3.94 | 3.91 | 3.87 |
| **2.28** | **1.91** | 4.17 | 4.41 |  |  |  | 4.28 |  |  |  | 4.84 |
| **2.28** | **0.97** | 3.35 | 3.29 | 3.32 | 3.34 | 3.29 | 3.18 | 3.29 | 3.29 | 3.33 | 3.32 |
| **2.22** | **2.17** | 2.28 | 2.28 | 2.28 | 2.29 | 2.29 | 2.28 | 2.28 | 2.28 | 2.28 | 2.28 |
| **2.22** | **2.13** | 3.41 |  |  |  |  |  |  |  |  |  |
| **2.22** | **2.03** | 3.67 | 3.81 | 3.78 | 3.8 | 3.78 | 3.78 | 3.83 | 3.84 | 3.84 | 3.65 |
| **2.22** | **1.91** | 2.25 | 2.26 | 2.26 | 2.26 | 2.26 | 2.26 | 2.26 | 2.26 | 2.26 | 2.25 |
| **2.17** | **1.91** | 2.79 | 2.77 | 2.77 | 2.78 | 2.79 | 2.81 | 2.78 | 2.76 | 2.81 | 2.78 |
| **2.22** | **1.48** | 2.81 | 2.83 | 2.82 | 2.82 | 2.82 | 2.81 | 2.82 | 2.82 | 2.83 | 2.82 |
| **2.17** | **1.48** | 2.72 | 2.71 | 2.7 | 2.7 | 2.72 | 2.7 | 2.71 | 2.68 | 2.71 | 2.72 |
| **2.22** | **0.97** | 3.11 | 3.09 | 3.1 | 3.11 | 3.09 | 3.18 | 3.09 | 3.1 | 3.09 | 3.08 |
| **2.17** | **0.97** | 2.46 | 2.46 | 2.46 | 2.46 | 2.46 | 2.46 | 2.45 | 2.45 | 2.46 | 2.46 |
| **2.22** | **0.86** | 3.59 |  | 3.6 | 3.6 | 3.62 | 3.57 |  | 3.57 | 3.59 | 3.66 |
| **2.13** | **2.03** | 3.35 | 3.31 | 3.31 | 3.3 | 3.3 | 3.3 | 3.3 | 3.3 | 3.31 | 3.3 |
| **2.13** | **1.48** | 4.82 | 4.58 | 4.48 | 4.46 | 4.45 | 4.46 | 4.48 | 4.46 | 4.53 | 4.49 |
| **2.13** | **1.23** |  |  |  | **5.17** |  |  |  |  |  |  |
| **2.09** | **1.23** | 2.9 | 2.9 | 2.91 | 2.9 | 2.9 | 2.9 | 2.9 | 2.9 | 2.91 | 2.9 |
| **2.13** | **0.97** | 4.38 | 4.18 | 4.11 | 4.29 | 4.18 | 4.17 | 4.09 | 4.24 | 4.19 | 4.21 |
| **2.03** | **1.74** | 3.98 | 3.97 | 3.94 | 3.96 | 3.95 | 3.94 | 3.87 | 3.86 | 3.99 | 3.91 |
| **2.03** | **1.3** | 3.89 | 3.93 | 3.81 | 3.91 | 3.9 | 3.89 | 3.9 | 3.92 | 4 | 3.93 |
| **1.91** | **1.48** | 2.39 | 2.39 | 2.39 | 2.39 | 2.39 | 2.39 | 2.39 | 2.39 | 2.39 | 2.39 |
| **1.91** | **1.4** | 4.15 | 4.24 | 4.09 | 4.16 | 4.09 | 4.11 | 4.14 | 4.17 | 4.08 | 4.16 |
| **1.91** | **0.97** | 2.62 | 2.62 | 2.62 | 2.62 | 2.62 | 2.62 | 2.62 | 2.62 | 2.62 | 2.62 |
| **1.91** | **0.86** | 2.71 | 2.71 | 2.71 | 2.71 | 2.71 | 2.71 | 2.71 | 2.71 | 2.71 | 2.71 |
| **1.81** | **1.66** | 2 | 2 | 2 | 2 | 2 | 2 | 2 | 2 | 2 | 2 |
| **1.81** | **1.14** | 3.56 | 3.57 | 3.55 | 3.57 | 3.57 | 3.58 | 3.58 | 3.58 | 3.57 | 3.58 |
| **1.81** | **1.05** | 2.9 | 2.9 | 2.9 | 2.89 | 2.9 | 2.9 | 2.9 | 2.89 | 2.9 | 2.89 |
| **1.74** | **1.4** | 2.65 | 2.65 | 2.64 | 2.65 | 2.64 | 2.65 | 2.64 | 2.65 | 2.65 | 2.64 |
| **1.74** | **1.3** | 2.78 | 2.77 | 2.88 | 2.89 | 2.89 | 2.89 | 2.89 | 2.89 | 2.89 | 2.77 |
| **1.66** | **1.05** | 3.09 | 3.09 | 3.09 | 3.09 | 3.09 | 3.09 | 3.09 | 3.08 | 3.09 | 3.08 |
| **1.48** | **0.97** | 1.78 | 1.78 | 1.78 | 1.78 | 1.78 | 1.78 | 1.78 | 1.78 | 1.78 | 1.78 |
| **1.48** | **0.86** | 2.66 | 2.66 | 2.66 | 2.65 | 2.66 | 2.65 | 2.66 | 2.65 | 2.66 | 2.66 |
| **1.4** | **1.3** | 2.6 | 2.61 | 2.61 | 2.61 | 2.61 | 2.61 | 2.61 | 2.61 | 2.61 | 2.61 |
| **1.3** | **1.23** | 3.55 | 3.43 | 3.42 | 3.42 | 3.42 | 3.44 | 3.44 | 3.43 | 3.45 | 3.45 |
| **1.09** | **1.05** | 3.23 | 3.2 | 3.2 | 3.19 | 3.19 | 3.2 | 3.19 | 3.19 | 3.2 | 3.19 |
| **0.97** | **0.86** | 2.76 | 2.78 | 2.79 | 2.78 | 2.78 | 2.78 | 2.78 | 2.78 | 2.78 | 2.78 |

Table S12: Interproton distances calculated from the quantile-based sampling schemes as compared to the uniformly sampled reference spectra. Red distances signify a greater than 7% deviation from the uniformly sampled data. Blue distances signify an interproton distance that was not valid in the uniformly sampled data but were valid in the NUS dataset (R^2^ > 0.90, n > 4). Assignments are given as chemical shifts (ppm).

| f2 δ  (ppm) | f1 δ  (ppm) | Ref.  r (Å) | Quantile |
| --- | --- | --- | --- |
| **9.7** | **6.21** | 4.56 | 4.61 |
| **9.7** | **4.5** | 4.21 | 4.19 |
| **9.7** | **4.38** | 4.21 | 4.21 |
| **9.7** | **4.02** | 4.16 | 4.16 |
| **9.7** | **3.84** | 3.01 | 3.02 |
| **9.7** | **3.65** | 4.38 | 4.38 |
| **9.7** | **3.35** | 3.86 | 3.99 |
| **9.7** | **2.67** | 3.23 | 3.24 |
| **9.7** | **2.22** | 3.19 | 3.2 |
| **9.7** | **2.17** | 3 | 3.02 |
| **9.7** | **1.91** | 3.75 | 3.79 |
| **9.7** | **1.48** | 3.61 | 3.63 |
| **9.7** | **1.14** | 3.89 | 3.92 |
| **6.21** | **6.05** | 2.97 | 2.97 |
| **6.21** | **5.57** | 2.53 | 2.53 |
| **6.21** | **4.5** | 3.67 | 3.67 |
| **6.21** | **4.42** | 4.21 | 4.21 |
| **6.21** | **4.02** | 2.53 | 2.53 |
| **6.21** | **3.84** | 3.85 | 3.84 |
| **6.21** | **3.65** | 2.84 | 2.84 |
| **6.21** | **3.03** | 3.85 | 3.94 |
| **6.21** | **2.67** | 4.17 | 3.9 |
| **6.21** | **2.22** | 3.74 | 3.75 |
| **6.21** | **2.17** | 2.38 | 2.38 |
| **6.21** | **2.09** | 3.73 | 3.73 |
| **6.21** | **1.91** | 3.47 | 3.47 |
| **6.21** | **1.48** | 4.15 | 3.98 |
| **6.21** | **0.97** | 3.38 | 3.37 |
| **6.21** | **0.86** | 4.05 | 4.02 |
| **6.05** | **5.57** | 2.49 | 2.49 |
| **6.05** | **4.96** | 4.02 | 4.02 |
| **6.05** | **4.02** | 3.91 | 3.92 |
| **6.05** | **3.65** | 3.43 | 3.43 |
| **6.05** | **2.28** | 3.98 | 3.98 |
| **6.05** | **2.09** | 2.3 | 2.31 |
| **6.05** | **1.91** |  | **4.94** |
| **6.05** | **1.23** | 4.55 | 4.56 |
| **6.05** | **0.97** | 3.52 | 3.51 |
| **6.05** | **0.86** | 4.03 | 4.02 |
| **5.57** | **4.96** | 2.99 | 2.99 |
| **5.57** | **4.5** | 3.86 | 3.85 |
| **5.57** | **4.02** | 3.31 | 3.3 |
| **5.57** | **3.65** | 3.25 | 3.25 |
| **5.57** | **3.03** | 3.76 | 3.75 |
| **5.57** | **2.28** | 4.41 | 4.3 |
| **5.57** | **2.17** | 3.54 | 3.48 |
| **5.57** | **2.09** | 2.71 | 2.8 |
| **5.57** | **1.91** | 3.61 | 3.61 |
| **5.57** | **1.48** | 3.22 | 3.23 |
| **5.57** | **1.23** | 4.18 | 4.23 |
| **5.57** | **0.97** | 2.77 | 2.77 |
| **5.57** | **0.86** | 2.87 | 2.87 |
| **4.96** | **4.02** |  | **4.38** |
| **4.96** | **3.91** | 4.14 | 3.76 |
| **4.96** | **3.35** | 4.38 |  |
| **4.96** | **3.14** | 3.59 | 3.6 |
| **4.96** | **3.08** | 2.72 | 2.72 |
| **4.96** | **2.77** | 4.05 | 4.07 |
| **4.96** | **2.35** | 3.76 | 3.78 |
| **4.96** | **2.09** | 2.76 | 2.77 |
| **4.96** | **1.81** | 2.84 | 2.84 |
| **4.96** | **1.66** | 2.48 | 2.48 |
| **4.96** | **1.23** | 2.97 | 2.97 |
| **4.96** | **1.14** | 3.01 | 3.01 |
| **4.9** | **4.5** | 3.53 | 3.53 |
| **4.9** | **4.38** | 3.38 | 3.38 |
| **4.9** | **4.08** | 3.28 | 3.27 |
| **4.9** | **3.91** | 2.38 | 2.38 |
| **4.9** | **3.84** | 3.91 | 3.93 |
| **4.9** | **3.39** | 3.17 | 3.18 |
| **4.9** | **3.35** | 2.53 | 2.54 |
| **4.9** | **3.08** | 3.8 | 3.8 |
| **4.9** | **3.03** | 3.96 | 3.95 |
| **4.9** | **2.42** | 3.25 | 3.25 |
| **4.9** | **2.35** | 3.21 | 3.16 |
| **4.9** | **1.81** | 4.76 |  |
| **4.9** | **1.48** |  | **4.04** |
| **4.5** | **4.38** | 4.46 |  |
| **4.5** | **4.08** | 3.27 | 3.27 |
| **4.5** | **3.91** | 2.82 | 2.82 |
| **4.5** | **3.84** | 3.03 | 3.04 |
| **4.5** | **3.65** | 2.66 | 2.66 |
| **4.5** | **3.03** | 3.65 | 3.59 |
| **4.5** | **2.28** | 3.39 | 3.37 |
| **4.5** | **2.17** | 2.8 | 2.78 |
| **4.42** | **4.02** | 3.34 | 3.34 |
| **4.42** | **3.91** | 4.06 | 4.02 |
| **4.38** | **3.91** | 4.26 | 4.18 |
| **4.38** | **3.84** | 2.28 | 2.28 |
| **4.38** | **3.65** | 4.05 |  |
| **4.42** | **3.43** | 3.63 | 3.64 |
| **4.38** | **3.39** | 2.99 | 3 |
| **4.38** | **3.35** | 3.32 | 3.33 |
| **4.38** | **3.14** | 2.25 | 2.25 |
| **4.38** | **3.08** | 3.16 | 3.16 |
| **4.38** | **3.03** | 3.33 | 3.32 |
| **4.42** | **2.67** | 3.71 | 3.71 |
| **4.38** | **2.67** | 3.78 | 3.81 |
| **4.38** | **2.42** |  | **3.69** |
| **4.38** | **2.35** | 2.41 | 2.41 |
| **4.42** | **2.22** | 3.38 | 3.38 |
| **4.38** | **2.22** | 3.69 | 3.68 |
| **4.42** | **1.91** | 2.59 | 2.59 |
| **4.42** | **1.74** | 3.42 | 3.44 |
| **4.38** | **1.48** | 4.52 |  |
| **4.42** | **1.4** | 2.73 | 2.73 |
| **4.38** | **1.14** | 4.08 | 4.1 |
| **4.42** | **0.86** | 3.6 | 3.6 |
| **4.08** | **3.91** | 2.28 | 2.28 |
| **4.08** | **1.91** | 4.65 |  |
| **4.08** | **1.14** | 3.69 | 3.72 |
| **4.02** | **3.91** | 2.89 | 2.88 |
| **4.02** | **3.84** | 3.86 | 3.81 |
| **4.02** | **3.65** | 3.6 | 3.58 |
| **4.02** | **3.08** | 3.16 | 3.16 |
| **4.02** | **2.77** | 3.11 | 3.11 |
| **4.02** | **2.67** | 2.92 | 2.92 |
| **4.02** | **2.42** | 3.54 | 3.54 |
| **4.02** | **2.22** | 2.53 | 2.53 |
| **4.02** | **2.17** | 2.41 | 2.41 |
| **4.02** | **1.91** | 2.61 | 2.61 |
| **4.02** | **1.48** | 3.37 | 3.36 |
| **4.02** | **1.3** |  | **4.83** |
| **4.02** | **1.14** | 3.36 | 3.36 |
| **4.02** | **0.97** | 3.28 | 3.27 |
| **4.02** | **0.86** | 3.75 | 3.73 |
| **3.91** | **3.14** | 3.91 | 3.84 |
| **3.91** | **3.08** | 3.89 | 3.83 |
| **3.91** | **2.77** | 3.84 | 3.8 |
| **3.91** | **2.35** | 3.18 | 3.17 |
| **3.91** | **1.81** | 3.27 | 3.26 |
| **3.91** | **1.66** | 3.75 | 3.72 |
| **3.91** | **1.05** | 3.65 | 3.61 |
| **3.84** | **3.65** | 2.9 | 2.9 |
| **3.84** | **3.39** | 3.51 | 3.5 |
| **3.84** | **3.14** | 3.1 | 3.12 |
| **3.84** | **3.08** | 4.22 | 4.29 |
| **3.84** | **3.03** | 2.91 | 2.91 |
| **3.84** | **2.67** | 2.79 | 2.79 |
| **3.84** | **2.28** | 3.73 | 3.93 |
| **3.84** | **2.22** | 2.78 | 2.78 |
| **3.84** | **2.17** | 2.21 | 2.21 |
| **3.84** | **1.91** | 3.49 | 3.51 |
| **3.84** | **1.48** | 3.41 | 3.3 |
| **3.84** | **0.97** | 3.11 | 3.12 |
| **3.65** | **3.39** | 3.4 | 3.36 |
| **3.65** | **3.03** | 2.35 | 2.35 |
| **3.65** | **2.67** | 3.7 |  |
| **3.65** | **2.42** | 3.23 | 3.23 |
| **3.65** | **2.28** | 2.55 | 2.54 |
| **3.65** | **2.22** | 3.54 | 3.53 |
| **3.65** | **2.17** | 2.13 | 2.13 |
| **3.65** | **1.91** | 3.66 | 3.6 |
| **3.65** | **1.48** | 2.97 | 2.92 |
| **3.65** | **0.97** | 2.6 | 2.6 |
| **3.39** | **3.14** |  | **4.24** |
| **3.39** | **3.03** | 2.48 | 2.48 |
| **3.39** | **2.42** | 3.27 | 3.27 |
| **3.39** | **2.35** | 4.01 | 3.96 |
| **3.39** | **2.28** | 2.7 | 2.7 |
| **3.43** | **2.13** | 3.54 | 3.54 |
| **3.43** | **1.4** | 3.69 | 3.68 |
| **3.39** | **0.97** | 3.65 | 3.64 |
| **3.35** | **3.14** | 3.57 | 3.65 |
| **3.35** | **3.08** | 2.65 | 2.65 |
| **3.35** | **2.42** | 2.73 | 2.73 |
| **3.35** | **2.35** | 2.99 | 2.99 |
| **3.14** | **3.08** | 2.59 | 2.59 |
| **3.08** | **2.67** | 4.08 | 4.1 |
| **3.14** | **2.35** | 2.47 | 2.47 |
| **3.08** | **2.42** | 3.12 | 3.11 |
| **3.14** | **1.81** | 4.03 | 4.03 |
| **3.08** | **1.81** | 4.12 | 4.12 |
| **3.08** | **1.66** | 3.76 | 3.66 |
| **3.14** | **1.14** | 3.1 | 3.11 |
| **3.08** | **1.14** | 3.15 | 3.15 |
| **3.03** | **2.67** | 4.06 | 4.09 |
| **3.03** | **2.42** | 3.39 | 3.41 |
| **3.03** | **2.28** | 2.51 | 2.51 |
| **3.03** | **2.22** | 3.62 | 3.59 |
| **3.03** | **2.17** | 2.79 | 2.8 |
| **3.03** | **1.91** | 3.77 |  |
| **3.03** | **1.48** | 2.54 | 2.55 |
| **3.03** | **0.97** | 2.25 | 2.25 |
| **3.03** | **0.86** | 3.85 | 3.81 |
| **2.77** | **2.28** | 3.91 | 4.2 |
| **2.77** | **1.81** | 3.48 | 3.48 |
| **2.77** | **1.66** | 2.85 | 2.85 |
| **2.77** | **1.14** | 3.34 | 3.34 |
| **2.77** | **1.05** | 3.54 | 3.52 |
| **2.67** | **2.22** | 1.86 | 1.86 |
| **2.67** | **2.17** | 2.39 | 2.38 |
| **2.67** | **1.91** | 2.58 | 2.58 |
| **2.67** | **1.48** | 2.77 | 2.76 |
| **2.67** | **0.97** | 3.05 | 3.04 |
| **2.42** | **2.35** | 2.8 | 2.74 |
| **2.42** | **2.28** | 2.2 | 2.2 |
| **2.42** | **1.14** | 3.82 | 3.83 |
| **2.28** | **1.91** | 4.17 |  |
| **2.28** | **0.97** | 3.35 | 3.31 |
| **2.22** | **2.17** | 2.28 | 2.28 |
| **2.22** | **2.13** | 3.41 |  |
| **2.22** | **2.03** | 3.67 | 3.73 |
| **2.22** | **1.91** | 2.25 | 2.25 |
| **2.17** | **1.91** | 2.79 | 2.77 |
| **2.22** | **1.48** | 2.81 | 2.81 |
| **2.17** | **1.48** | 2.72 | 2.7 |
| **2.22** | **0.97** | 3.11 | 3.1 |
| **2.17** | **0.97** | 2.46 | 2.45 |
| **2.22** | **0.86** | 3.59 | 3.59 |
| **2.13** | **2.03** | 3.35 | 3.32 |
| **2.13** | **1.48** | 4.82 | 4.51 |
| **2.09** | **1.23** | 2.9 | 2.9 |
| **2.13** | **0.97** | 4.38 | 4.26 |
| **2.03** | **1.74** | 3.98 | 3.93 |
| **2.03** | **1.3** | 3.89 | 3.9 |
| **1.91** | **1.48** | 2.39 | 2.39 |
| **1.91** | **1.4** | 4.15 | 4.17 |
| **1.91** | **0.97** | 2.62 | 2.62 |
| **1.91** | **0.86** | 2.71 | 2.71 |
| **1.81** | **1.66** | 2 | 2 |
| **1.81** | **1.14** | 3.56 | 3.55 |
| **1.81** | **1.05** | 2.9 | 2.89 |
| **1.74** | **1.4** | 2.65 | 2.65 |
| **1.74** | **1.3** | 2.78 | 2.89 |
| **1.66** | **1.05** | 3.09 | 3.08 |
| **1.48** | **0.97** | 1.78 | 1.78 |
| **1.48** | **0.86** | 2.66 | 2.65 |
| **1.4** | **1.3** | 2.6 | 2.61 |
| **1.3** | **1.23** | 3.55 | 3.43 |
| **1.09** | **1.05** | 3.23 | 3.19 |
| **0.97** | **0.86** | 2.76 | 2.78 |
